# Regioswitchable Bingel Bis-Functionalization of Fullerene C_70_ via Supramolecular Masks

**DOI:** 10.1021/jacs.3c10808

**Published:** 2024-02-05

**Authors:** Valentina Iannace, Clara Sabrià, Youzhi Xu, Max von Delius, Inhar Imaz, Daniel Maspoch, Ferran Feixas, Xavi Ribas

**Affiliations:** † Institut de Química Computacional i Catàlisi (IQCC) and Departament de Química, Universitat de Girona, Campus Montilivi, 17003 Girona, Catalonia, Spain; ‡ Institute of Organic Chemistry, University of Ulm, Albert-Einstein-Allee 11, 89081 Ulm, Germany; § Catalan Institute of Nanoscience and Nanotechnology (ICN2), CSIC and The Barcelona Institute of Science and Technology, Campus UAB, 08193 Bellaterra, Catalonia, Spain; ⊥ ICREA, Passeig de Lluís Companys 23, 08010 Barcelona, Catalonia, Spain

## Abstract

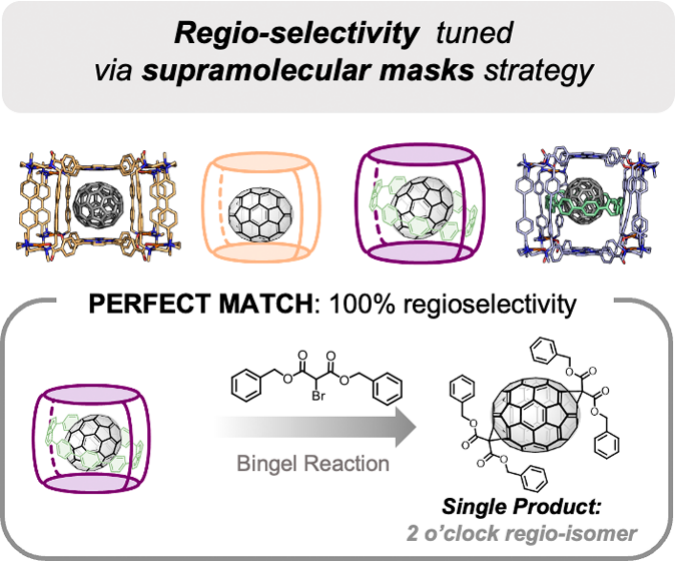

Isomer-pure functionalized fullerenes are required to boost the development of fullerene chemistry in any field, but their multiple functionalization renders a mixture of regioisomers that are very difficult to purify by chromatography. For the specific case of C_70_, its nonspherical geometry makes its regioselective functionalization more challenging than that of spherical C_60_. In this work, the supramolecular mask approach is applied for the first time to C_70_, which is encapsulated in two different nanocapsules to achieve the Bingel bis-cyclopropanation at α-bonds of opposite poles. Based on the tetragonal prismatic geometry imposed by the smaller supramolecular mask tested, the obtained major bis-adduct is completely reversed (major 5 o’clock) compared to bare C_70_ functionalization (major 2 o’clock). Moreover, by further restricting the accessibility of C_70_ using a three-shell Matryoshka mask and dibenzyl-bromomalonate, a single regiospecific 2 o’clock bis-isomer is obtained, owing to the perfect complementarity of the mask and the addend steric properties. The outcome of the reactions is fully explained at the molecular level by means of a thorough molecular dynamics (MD) study of the accessibility of the α-bonds to produce the different bis-adducts.

## Introduction

**Figure 1:**
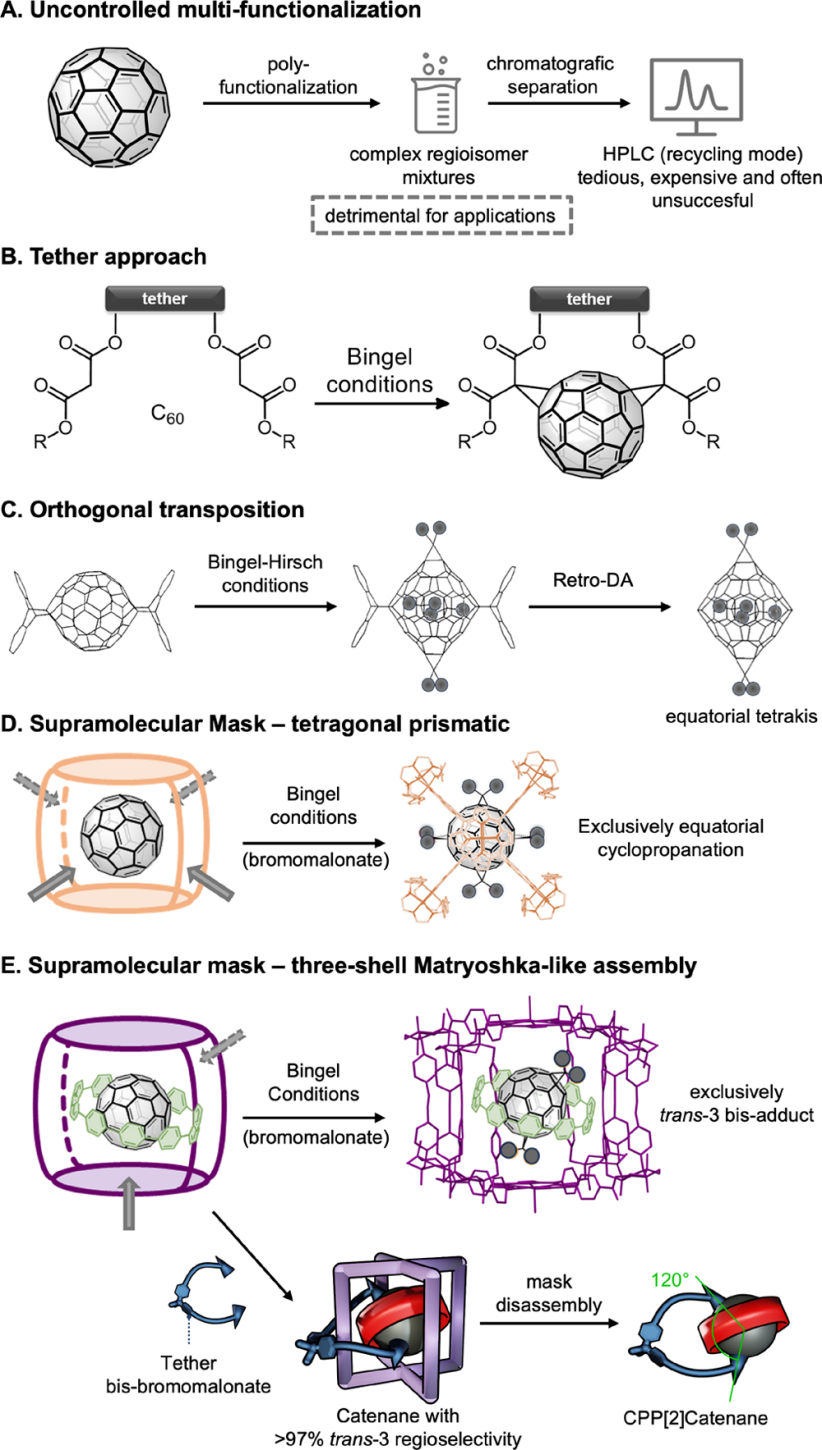
Current regio-functionalization strategies of fullerene C_60_.

Fullerenes are privileged electron transporting materials (ETM) widely used in organic solar cells (OSCs) and perovskite solar cells (PSCs).^1^ The high electron mobilities and 3D spheroidal character of fullerenes result in robust charge delocalization and high power conversion efficiency (PCE). However, only easily accessible and solution-processable monofunctionalized PCBM ([6,6]-phenyl-butyric acid methyl ester) derivatives of C_60_ and C_70_ are typically considered as viable ETM contenders.^2−6^ As a consequence, there is a serious lack of diversity in fullerene-based ETM for PSCs, a clear drawback in the development of new generation cells. In particular, pure polyfunctionalized fullerenes are highly desirable to boost the development of fullerene-based materials,^3,6,7^ but they are practically inaccessible due to the formation of intractable mixtures of tens of regioisomers when there are two or more addends on the fullerene (Figure 1A).^8−11^ Very few strategies, reported in the 1990s, exist to overcome this resounding limitation: the “tether-directed remote functionalization” strategy for the synthesis of polycyclopropanated adducts (Bingel-Hirsch) under regiocontrol (Figure 1B)^12−15^ and the “orthogonal transposition” for the production of equatorial tetrakis-cyclopropanated-C_60_ adducts (Figure 1C).^16,17^ Recently, supramolecular masks have emerged as a valid way to have practical access to isomerically pure polyfunctionalized C_60_.^18^ Pioneered by our group, this approach consists of designing supramolecular hosts with a high affinity for fullerenes and then subjecting the resulting host–guest adduct to fullerene functionalization conditions, allowing the chemo- and regioselective derivatization only at the exposed surface of the fullerene left by the supramolecular mask. In this manner, we reported the sequential synthesis of bis-, tris-, or tetrakis-equatorial adducts in a selective manner using Bingel cyclopropanated addends (Figure 1D)^19^ or acene cycloadded addends.^20^ Also recently, a supramolecular three-shell Matryoshka-like complex featuring C_60_ encapsulation was reported and used as a mask to deliver *trans*-3 bis-adducts^21^ and [2]catenanes incorporating both C_60_ and [10]CPP nanohoop (Figure 1E).^22^

**Figure 2:**
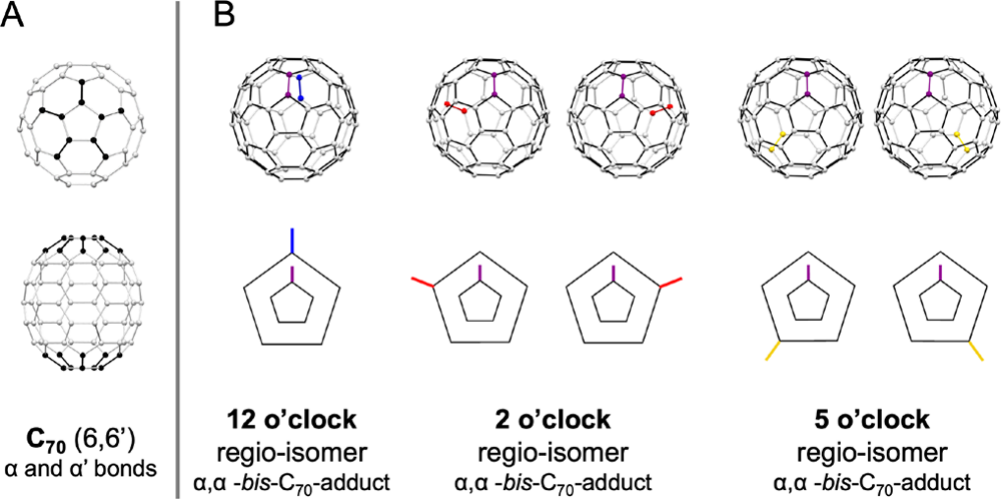
(A) α and α′ (6,6′) bonds of C_70_. (B) The three possible regioisomers of a bis-α,α-C_70_-adduct (Schlegel diagram using the Newman-type projection, and with the C_70_ core viewed along the C_5_ axis passing through the proximal (small) and distal (large) pentagons at the opposite poles of the fullerene).

Less is known about the control of the mono- and polyfunctionalization of C_70_.^23^ In contrast to a single possible isomer for monosubstituted-C_60_ due to its spherical geometry, there can exist eight different monosubstituted isomers of C_70_ due to its oval-shaped ellipsoidal geometry.^24,25^ Four of these bonds are located at the junction between two six-membered rings (6,6′ bonds), and the other four are at the junction between a six- and a five-membered ring (6,5′ bonds).^26,27^ Additionally, four types of (6,6′) bonds (α, β, γ, and δ) can render mixtures, as in the case of monosubstituted-C_70_ with PCBM ([6,6]-phenyl butyric acid methyl ester), where α and β addends can be obtained.^28,29^ Consequently, regioselective bis-functionalization of C_70_ entails much higher complexity than C_60_. Bingel regiofunctionalization is chosen as a way to minimize the number of isomers formed during the first addition step since it shows a strong preference for the (6,6′) α-type bonds due to their highly pyramidalized C_sp^2^_ atoms (Figure 2A).^26,27,30^ Hence, after the first addend addition on an α-bond on one pole of C_70_, the bis-adduct formation would preferentially occur at the five reactive (6,6′) α-bonds of the second pole.^26,27^ If the malonate addend is symmetric, then bis addition to α-type bonds on opposite poles yields three constitutionally isomeric bis-fullerene Bingel adducts, named 12 o’clock, 2 o’clock, or 5 o’clock (Figure 2B). These regioisomers present characteristic UV–vis spectra that do not depend on the type of adduct but on the regioisomer nature.^26,27^

Performing Bingel functionalization using standard conditions (bromomalonate, DBU, toluene, 25 °C) yields a mixture of Bingel bis-C_70_ adducts in an approximate ratio of 2.8:6.2:1 for 12 o’clock:2 o’clock:5 o’clock.^26^ Tether-directed strategies have also been used on C_70_ to obtain controlled regioselectivity of bis regioisomers.^31,32^ Regarding its polyfunctionalization, a single *C*_2_-symmetrical isomer of the tetrakis-Bingel-C_70_, featuring a functionalized α and β bond at each pole, has been reported using bromomalonate/DBU/CCl_4_/25 °C.^27^ Moreover, polychlorination under very harsh conditions also allows for a controlled regioisomeric outcome to obtain C_70_Cl_26_ and subsequent rearrangements, as described by Troyanov and co-workers.^33^

No example of the use of supramolecular masks for controlling the regiofunctionalization of C_70_ has been reported, in spite of a significant number of reports describing the enhanced affinity of a specific host for C_70_ over C_60_.^34−43^ In this work, we challenge the regio-control ability of supramolecular masks by performing the Bingel bis-cyclopropanation reaction on C_70_ upon encapsulation in a tetragonal prismatic nanocapsule,^44^ i.e., (C_70_**⊂4**·(BArF)_8_), and in a three-shell Matryoshka-like complex (C_70_**⊂**[10]CPP**⊂6**·(BArF)_8_).^21^ We explored a series of four symmetrical bromomalonates differing in steric bulk to determine the limits of the mask strategy. Using nanocapsule **4**·(BArF)_8_, we obtain the bis-C_70_-adduct in a reversed ratio (5 o’clock major) compared to bulk functionalization (2 o’clock major), although overfunctionalization is not prevented. On the other hand, in the case of the three-shell C_70_-Matryoshka-like complex, overfunctionalization is completely avoided, and a perfect match between the sterics of the nanocapsule structure and addend was found using dibenzyl-bromomalonate (**Bn**), exclusively obtaining the 2 o’clock regioisomer with ideal regioselectivity. In addition, MD simulations are successfully used to rationalize the experimental regioselectivity. Altogether, the supramolecular mask strategy is confirmed as a valuable tool for the synthesis of pure regioisomer-functionalized C_70_-fullerene for future dedicated applications.

## Results and Discussion

### Functionalization of Bare C_70_

**Figure 3:**
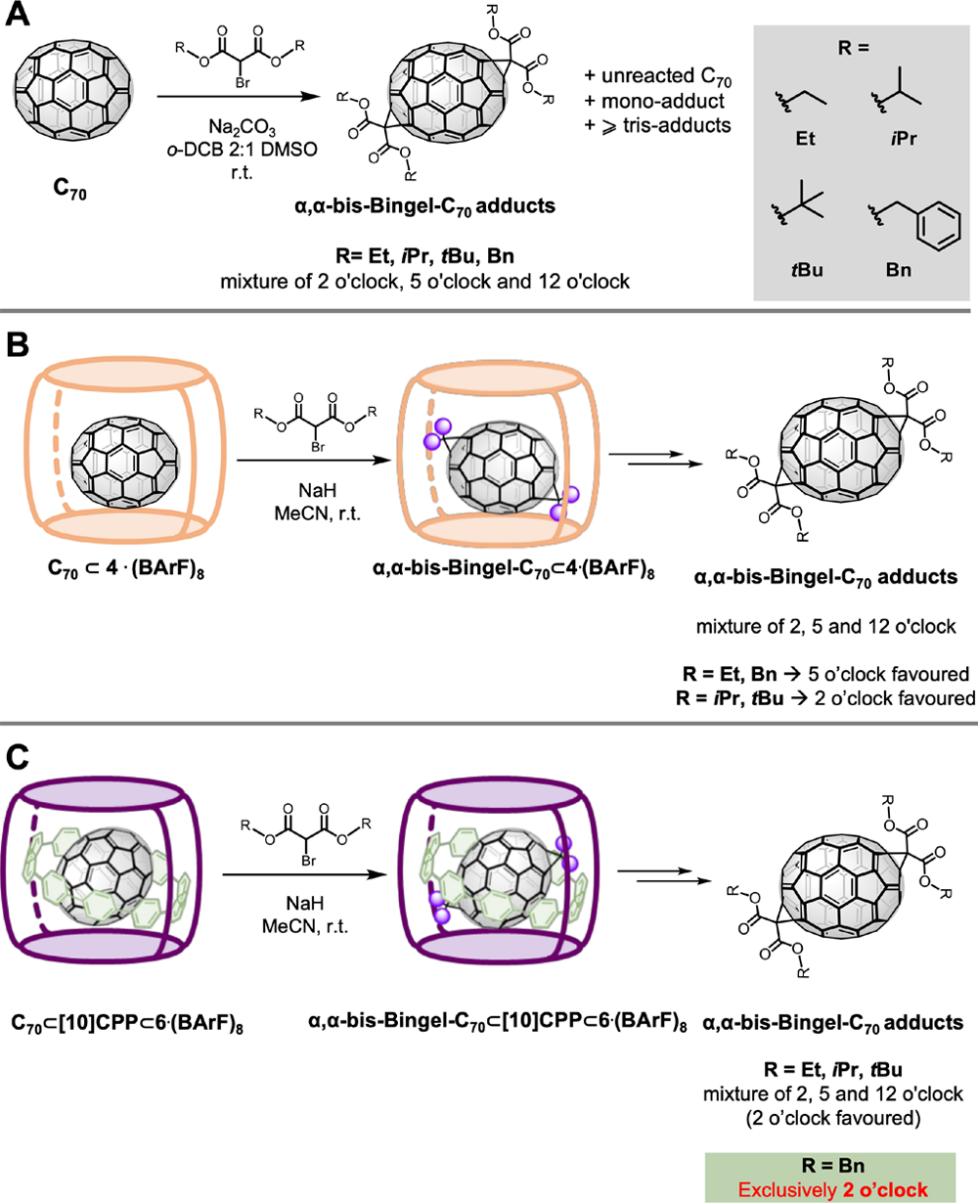
General procedures for the functionalization of C_70_. (A) Using bare C_70_; (B) using C_70_⊂**4**·(BArF)_8_; and (C) using C_70_⊂[10]CPP⊂**6**·(BArF)_8_.

We started this study by reproducing the reported functionalization of bare C_70_ as a reference experiment in which no restrictions are imposed on regioselectivity.^26^ The reaction was performed at room temperature in *o*-dichlorobenzene (*o*-DCB):DMSO (2:1) using Na_2_CO_3_ as a base), and using bromomalonates of different steric bulk: diethyl-bromomalonate (**Et**), di-isopropyl-bromomalonate (*i***Pr**), di-tert-butyl-bromomalonate (*t***Bu**), and dibenzyl-bromomalonate (**Bn**). The reaction was stopped when the bis-adduct formation was maximized (20–60 min), although bare C_70_, monoadduct, and polyadducts (>tris) were also found in the final mixture (TLC monitoring in DCM) (Figure 3A). Column chromatography was performed to collect the bis-adducts from a mixture also containing mono-, tris-, and higher adducts, which accounted for a low ∼5% yield of bis-adducts. Subsequent preparative TLC allowed isolating each bis-adduct, which was identified by the characteristic UV–vis spectra. In all cases, we obtained the 2 o’clock regioisomer as the major product (relative ratio 68–69%) and the 12 and 5 o’clock regioisomers in smaller amounts (relative ratios 10–21%).

Since it was reported that [10]CPP can impart some regioselectivity to C_60_,^45,46^ we prepared the supramolecular assembly C_70_⊂[10]CPP and exposed it to Bingel reaction conditions using the dibenzyl-bromomalonate. The obtained regioisomer ratios (2.0:6.8:1.2 ratio of 12:2:5 o’clock) were similar to those found for bare C_70_, thus indicating that the [10]CPP nanohoop does not impart any significant degree of control to the regioselectivity of C_70_ functionalization.

The above preliminary experiments clearly showed the intrinsic challenges in controlling the regioselectivity and the urgent need to a) prevent the polyfunctionalization and b) improve the regioselectivity of the reaction.

### Supramolecular Mask Strategy

**Figure 4:**
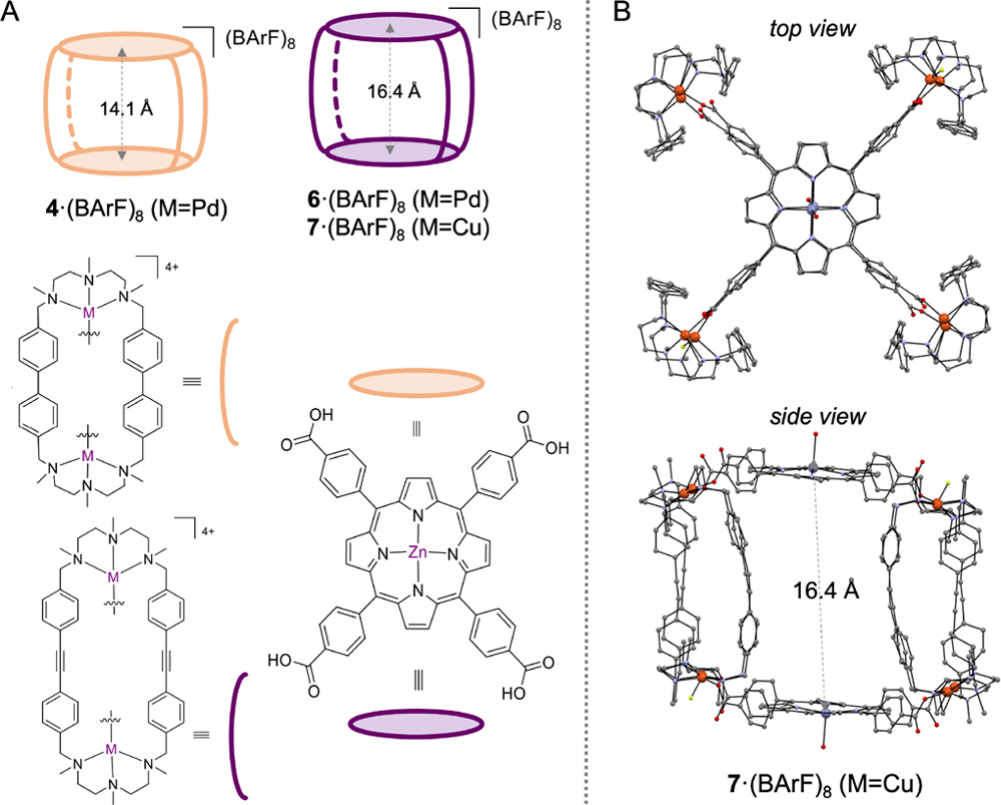
(A) Composition of Pd-based nanocapsules **4**·(BArF)_8_ and **6**·(BArF)_8_ and the Cu-based **7**·(BArF)_8_ nanocapsule (isostructural to **6**). (B) Crystal structure of **7**·(BArF)_8_ obtained by synchrotron radiation, top and side views.

C_70_ was encapsulated in a tetragonal prismatic Pd-based nanocapsule **4**·(BArF)_8_ (Figure 4), which presented a high affinity for fullerene C_70_ (*K*_a_ > 10^8^ M^–1^) as previously reported by our group.^44^ The encapsulation was performed analogously to the reported one (MeCN 1:9 toluene, 24 h, r.t.), and the formation of C_70_⊂**4**·(BArF)_8_ was confirmed by HR-MS (ESI-QTOF) (Figure S62).

**Figure 5:**
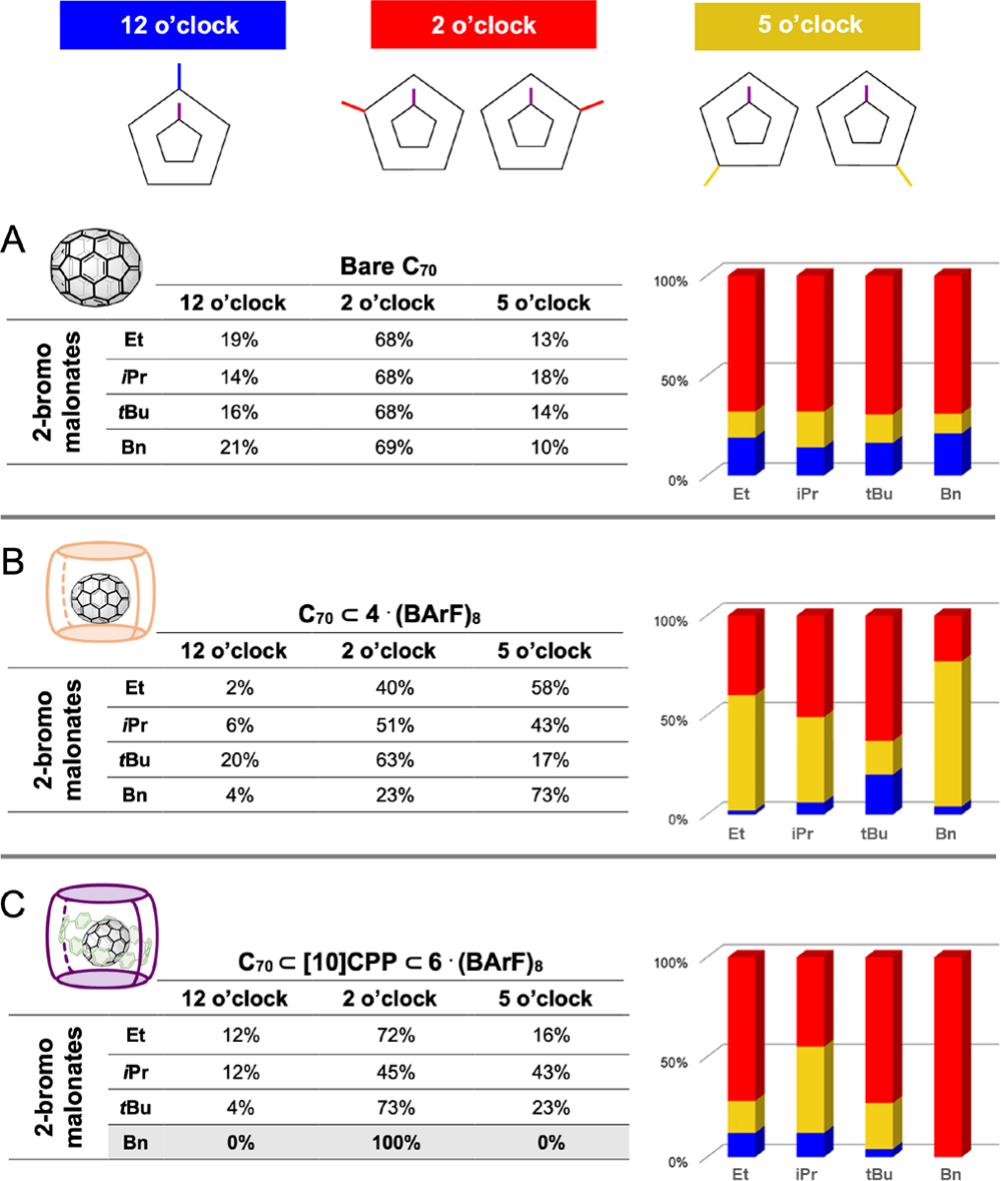
Experimental results obtained with the four bromomalonates (**Et**, *i***Pr**, *t***Bu**, and **Bn**) for (A) bare C_70_, (B) using nanocapsule **4**·(BArF)_8_, and (C) **C**_**70-**_**Matryoshka**.

With the host–guest complex C_70_⊂**4**·(BArF)_8_ in our hands, it was exposed to the Bingel reaction conditions using MeCN as the solvent and NaH as the base. By HR-MS monitoring (Figures S54–S57), we found that the functionalization process was slowed down compared to bare C_70_, although still a 1:1 mixture of tris- and tetrakis-adducts was obtained in 24 h. Since the focus of interest was the bis-adduct regioselectivity, we optimized the reaction conditions to obtain the bis-adducts as major products (yields in the range of 13–40%, depending on the malonate; 3 to 4 h) when a maximum of 4 equiv of the bromomalonates was used. The general procedure to obtain the targeted product required the disassembly of the nanocapsule with triflic acid to release the adducts (see the workflow in Figure S18). Then, the three regioisomers were separated by TLC and quantified by HPLC (see the ratios in Figure 5B). In most cases, the ratio of the 12 o’clock regioisomer was reduced (the special case of the bis-*t*Bu-C_70_-adduct is explained by MD simulations, see below), whereas the ratio of the 5 o’clock regioisomer was clearly improved. Indeed, for the diethyl- (**Et**) and dibenzyl- (**Bn**) bromomalonate, the favored product switched from 2 o’clock to 5 o’clock regioisomers up to a ratio of 42:58 for **Et** and a remarkable ratio of 23:73 for **Bn** (Figure 5B).

To restrict the regioselectivity even further and to gain control over the number of additions (“itero-selectivity”),^47^**C**_**70**_**-Matryoshka**, i.e., C_70_⊂[10]CPP⊂**6**·(BArF)_8_, was synthesized. We initially prepared the C_70_⊂[10]CPP assembly, and then it was accommodated in the larger nanocapsule **6**·(BArF)_8_, taking inspiration from our previous work with C_60_.^21^ The structure of the Cu-based **7**·(BArF)_8_ empty nanocapsule (isostructural to the Pd-based **6**·(BArF)_8_) was elucidated by synchrotron radiation (Figure 4B). As expected, it featured a tetragonal prismatic capsule with a suitable large cavity (*d*_ZnPorph···ZnPorph_ = 16.46 Å) to host the C_70_⊂[10]CPP guest. The three-shell **C**_**70**_**-Matryoshka** was thus obtained by mixing both C_70_⊂[10]CPP and **6**·(BArF)_8_ nanocapsules in a mixture of MeCN:DCM (1:1) at room temperature overnight. Its full formation was confirmed by HRMS (ESI-QTOF) (Figure S67). The single crystal structure of C_70_⊂[10]CPP⊂**6**·(BArF)_8_ was repeatedly attempted at the synchrotron but was unsuccessful, presumably due to the intrinsic dynamics of the three-shell **C**_**70**_**-Matryoshka** (see MD simulations below).

Remarkably, when the three-shell **C**_**70**_**-Matryoshka** was exposed to the Bingel reaction conditions in MeCN, we observed only the appearance of bis-adduct peaks by HRMS monitoring (see Figures S58–S61). Identical results were seen when an excess of bromomalonate (8–10 equiv) and base NaH (8–10 equiv) were used for 24 h at room temperature. This observation clearly indicated that our three-shell supramolecular mask prevents the polyfunctionalization over bis-adducts. The removal of the encapsulated bis-C_70_-adduct was achieved by the addition of an excess of fullerene C_60_ or C_70_, which have a higher affinity than bis-functionalized-C_70_. In this way, the bis-adducts were released in chloroform and a reusable **C_60_-** or **C_70_-Matryoshka** was created (see the workflow in Figure S24). For all the bromomalonates used (**Et**, *i***Pr**, *t***Bu**, and **Bn**), bis-adducts were solely obtained in a yield ranging from 29 to 43%. The bis-α,α-regioisomer were separated by TLC and quantified by HPLC. Comparing the ratios of bis-α,α-regioisomers obtained using the **C**_**70**_**-Matryoshka** mask (Figure 5C) with the ones synthesized using the supramolecular mask **4**·(BArF)_8_ (Figure 5B), the ratio for 12 o’clock was again reduced. On the contrary, the three-shell mask was not favoring the 5 o’clock regioisomer in any case, and 2 o’clock was the major regioisomer. Remarkably, the steric bulk of the malonate is key to achieving a proper match, and in the case of the dibenzyl bromomalonate (**Bn**), the 2 o’clock regioisomer was obtained exclusively with an ideal regioselectivity. To our knowledge, this is the first reported pure-isomer bis-Bingel functionalization of C_70_.

Attempts to perform the reaction using a catalytic amount of C_70_⊂[10]CPP⊂[**6**·(BArF)_8_] and excess of C_70_ were made in pure MeCN, but very low yields of 2-o’clock bis-adduct were obtained. Evident nanocapsule decomposition due to a large excess of base hampered any turnover, albeit only the 2-o’clock bis-adduct was formed because the reaction occurred exclusively in the confined space of the Matryoshka.

### Molecular Dynamics (MD) Simulations to Unravel Fullerene Accessibility

MD simulations for all cases were performed to rationalize the molecular basis of the observed experimental ratios between the bis-adduct regioisomers using both supramolecular masks. Our previous work showed that unfunctionalized C_70_ horizontally freely rotates inside the **4**·(BArF)_8_, sampling multiple orientations.^48^ This indicates that fullerene rotation is not restricted by the nanocapsule and that the α-bonds of both poles are equally exposed. Here, we hypothesized that both the supramolecular mask and the interactions established by the monoadducts with the nanocapsule will precisely control the dynamics of the fullerene and the accessibility of the α-bonds to achieve the regioselective formation of bis-adducts.

**Figure 6:**
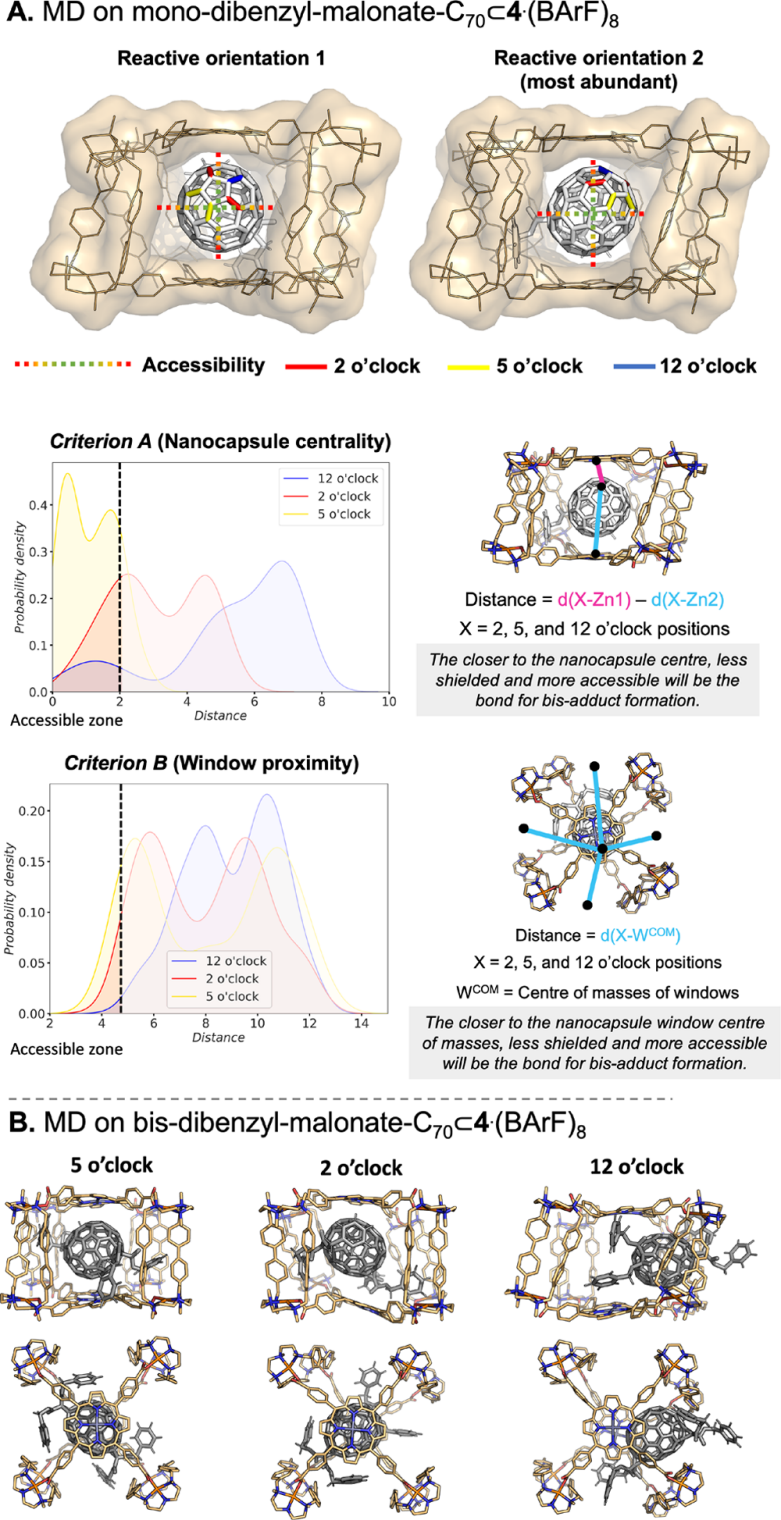
(A) MD studies on mono-di-benzyl-malonate-C_70_⊂**4**·(BArF)_8_ and accessibility analysis using criteria A and B. Space-filling models of two representative structures are used to show the accessibility of the α-bonds. Probability density plots of criteria A and B obtained from five replicas of 500 ns of MD simulations (all distances are in Å) where X = 2, 5, and 12 o’clock centers of mass; Zn1 and Zn2 = the position of the zinc ions of the porphyrin rings; and W^COM^ = Centre of masses of nanocapsule windows. The thresholds of the accessible zones are determined based on nonmasked regions in the space filling models. (B) Representative structures of bis-dibenzyl-malonate-C_70_⊂**4**·(BArF)_8_ obtained from MD simulations.

All MD simulations were performed starting from monoadduct-C_70_ placed inside the nanocapsule using either **4**·(BArF)_8_, i.e., mono-C_70_⊂**4**·(BArF)_8_, or the **C**_**70**_**-Matryoshka** complex, i.e., mono-C_70_⊂[10]CPP⊂**6**·(BArF)_8_, performing five replicates of 0.5 μs for each system. Since the bis-adduct formation must take place through one of the four windows of the nanocapsule, the accessibility of the three different bonds of the mono-C_70_ that will deliver the 2 o’clock, 5 o’clock, and 12 o’clock bis-adducts has been assessed using different criteria: criterion A) monitoring the distance between each α-bond and the porphyrins (nanocapsule centrality); criterion B) focusing on the proximity of each α-bond to the center of the window of the nanocapsule (window proximity); and Criterion C) in the case of the **C**_**70**_**-Matryoshka** complex, focusing on their distance from the [10]CPP ring (blocked by the nanohoop) (further details in Figures 6 and 8 and the SI). Altogether, we considered that the bond that is placed farther from both porphyrins, closer to the center of the window, and is less shielded by the [10]CPP ring will be the most accessible. The position with more accessibility (less shielded by the supramolecular mask) will probably be the one that reacts more, so the yield of the corresponding bis-adduct would be higher. Additionally, each bis-adduct has been simulated to study its conformation inside the corresponding mask and to estimate its binding free energy (Δ*G*_bind_). We have conducted a comprehensive MD study with the two masks and the four bromomalonates (complete analysis in section 6 in the SI), although we focus our discussion on the dibenzyl-malonate addend since it shows the best steric complementarity among host and guest and therefore the effect of each supramolecular mask is maximized.

In the case of the (6,6′) α-bond mono-di-benzylmalonate-C_70_ adduct encapsulated in nanocapsule **4**·(BArF)_8_, we have specifically explored the accessibility to the α-bonds of the second pole through the gates of the mask. By collectively analyzing all MD replicates, we observed that the 5 o’clock position followed by 2 o’clock are the most accessible considering criteria A and B (Figure 6). The visual inspection of MD simulations revealed that the encapsulated mono-di-benzylmalonate-C_70_ adduct scans two major orientations inside the nanocapsule, showing reduced rotation compared to unfunctionalized C_70_ (Figure 6 and Videos S1 and S2).^48^ In the most populated one, the dibenzylmalonate moiety is stabilized between two clips, fixing the orientation of C_70_ and exposing the opposite pole in the opposite window. In the second orientation, the malonate is stabilized by only one clip, with each phenyl moiety placed in the center of a different window, which makes the α-bonds accessible from the adjacent windows (Figure S73). In agreement with the distance criteria, in both orientations the 5 o’clock is the position located closer to the center of the nanocapsule window. The other two positions are less accessible in both orientations, which indicates that they are pointing more toward one porphyrin or shielded by the window clips. These results are in agreement with the experimental higher relative ratio of 5 o’clock (0.4:2.3:7.3 ratio for 12:2:5 o’clock).

The final 12, 2, and 5 o’clock bis-adducts were also simulated upon encapsulation, showing that the 5 o’clock bis-adduct indeed adopts the most stable complex with the cage with a binding energy of −77.5 kcal/mol (see Figure 6). For the 2 o’clock bis-adduct, a stable complex is also observed, with a relative binding energy of +0.9 kcal/mol with respect to the 5 o’clock. Finally, for the 12 o’clock adduct, it adopts a less stable off-centered stabilization at one of the gates of the nanocapsule, with a relative binding energy of +5.4 kcal/mol. Along with the accessibility studies, the less thermodynamically stable 12 o’clock bis-adduct is translated in a residual yield obtained experimentally, and the most stable 5 o’clock bis-adduct is the major regioisomer obtained.

**Figure 7:**
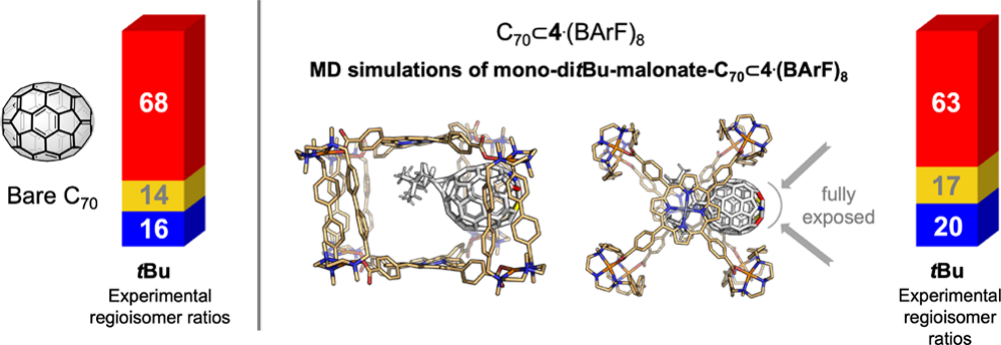
Comparative experimental regioisomer ratios found for bare C_70_ (left) and C_70_⊂**4**·(BArF)_8_ (right, along with the MD simulations of mono-di-^*t*^Bu-malonate-C_70_⊂**4**·(BArF)_8_ predicting full exposure of the fullerene. Color code: blue (12 o’clock), red (2 o’clock), and yellow (5 o’clock).

An analogous predictive analysis is performed for the diethyl-malonate and diisopropyl-malonate addends, with results in agreement with the relative ratios experimentally observed (see Figures S74 and S75). On the contrary, the experimentally observed trend of enhanced formation of the 5 o’clock addend is absent for di-tert-butyl-malonate addends, since the major bis-adduct obtained is the 2 o’clock and the ratio distribution is very similar to the one obtained with bare C_70_ (Figure 5). MD simulations of mono-di-^*t*^Bu-malonate-C_70_⊂**4**·(BArF)_8_ shed light on this result, since in three out of five replicates the mono-di-^*t*^Bu-malonate-C_70_ adduct is displaced toward one of the entries of the nanocapsule (Figures 7 and S76). This conformation is equivalent to the functionalization of bare C_70_ since the nanocapsule cannot exhibit its mask action due to the almost complete exposure of the unfunctionalized pole of C_70_.

**Figure 8:**
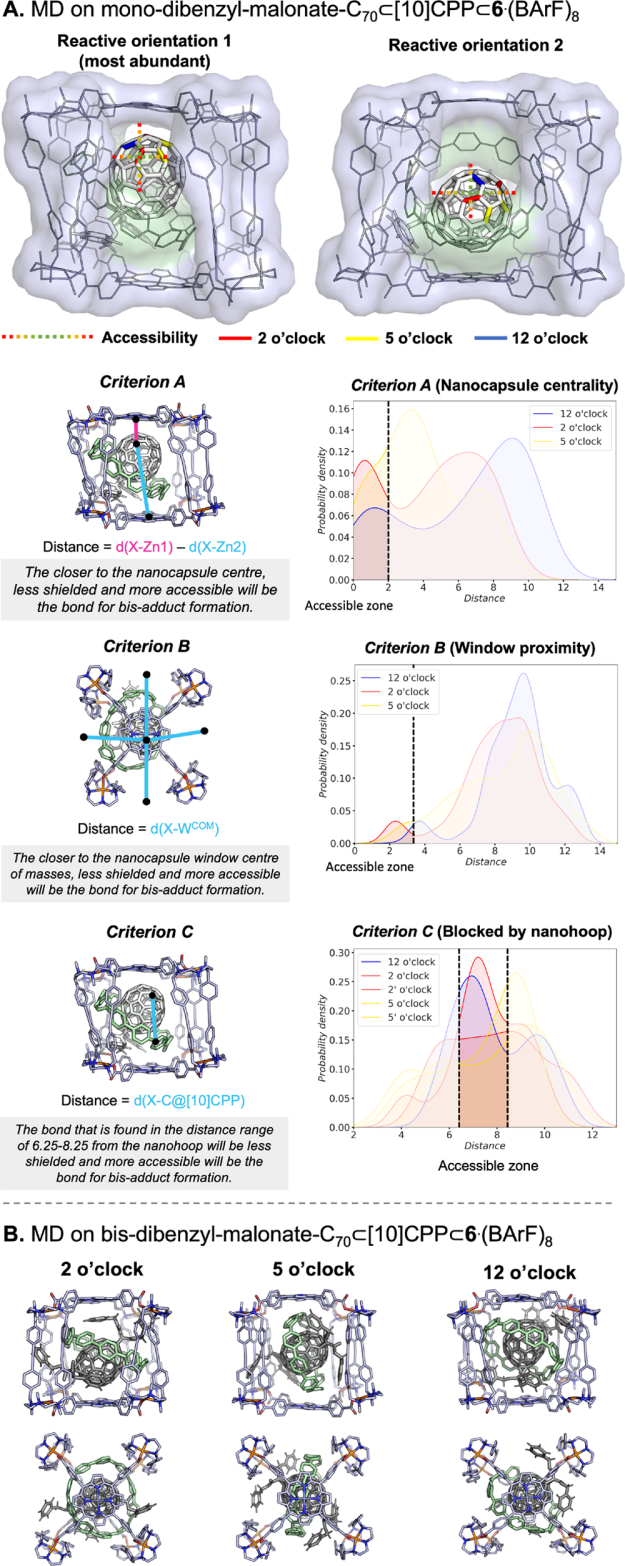
(A) MD studies on mono-di-benzyl-malonate-C_70_ ⊂[10]CPP⊂**6**·(BArF)_8_ and accessibility analysis using criteria A, B, and C. Space-filling models of two representative structures are used to show the accessibility of the α-bonds. Probability density plots of criteria A and B obtained from five replicas of 500 ns of MD simulations (all distances are in Å) where X = 2, 5, and 12 o’clock centers of mass; Zn1 and Zn2 are the positions of the zinc ions of the porphyrin rings; W^COM^ represents the centers of mass of nanocapsule windows; and C@[10]CPP is a carbon atom in the [10]CPP nanohoop. The thresholds of the accessible zones are determined based on nonmasked regions of the space-filling models. (B) Representative structures of bis-dibenzyl-malonate-C_70_⊂[10]CPP⊂**6**·(BArF)_8_ obtained from MD simulations.

We then analyzed the case of the mono-di-benzylmalonate-C_70_ adduct encapsulated in the Matryoshka assembly, i.e., mono-di-benzylmalonate-C_70_⊂[10]CPP⊂**6**·(BArF)_8_. Using criteria A and B, the most accessible position is 2 o’clock, which is the only bis-adduct obtained experimentally (Figure 8 and Videos S3 and S4). Since the [10]CPP ring is now present and may block the accessibility to C_70_, we established criterion C, which accounts for the ability of [10]CPP to block 2, 5, and 12 o’clock positions (Figure 8). With criterion C, the accessible positions, not blocked by [10]CPP, are 12 o’clock and one of the 2 o’clocks. The visual inspection of MD simulations reveals that the supramolecular mask reduces the dynamicity of the mono-di-benzylmalonate-C_70_ adduct, which is anchored through a network of interactions between the dibenzylmalonate addend and the nanocapsule clips. In these orientations, the 12 o’clock regioisomer is always placed backward and the 2 o’clock is facing the gate of the nanocapsule **6**·(BArF)_8_ (Figure 8A), thus the simulations clearly suggest that 2 o’clock is the most favored. Interestingly, both 5 o’clock positions are generally shielded by the nanohoop in [10]CPP⊂**6**·(BArF)_8_, which may be key to reverting the regiofunctionalization trends observed in **4**·(BArF)_8_.

Also analyzing the bis-dibenzyl-C_70_ adducts inside the three-shell Matryoshka complex, we observe that, in the case of the 5 o’clock regioisomer, the [10]CPP ring rotates and it is placed vertically with respect to the porphyrin planes. This state tenses the cage to higher Zn–Zn distances among nanocapsule **6**·(BArF)_8_ (*d*_ZnPorph···ZnPorph_ > 18 Å), which renders this conformation highly unstable and residual. In contrast, for the 2 o’clock regioisomer, we observe a stable conformation in which one of the phenyl substituents of the addend establishes an interaction with the porphyrin and the other addend interacts with the phenyls of the clips (Figure 8B and Video S5). In addition, the average Zn···Zn distances are the same as those for the initial Matryoshka assembly, so the nanocapsule **6**·(BArF)_8_ is likely not strained. This perfect match between the 2 o’clock bis-dibenzyl-C_70_ adduct and the [10]CPP⊂nanocapsule allows for a favored accommodation and perfect 100% regioselectivity for the 2 o’clock regioisomer.

The MD analysis of most accessible bis-adducts with diethyl-malonate and diisopropyl-malonate addends utilizing criteria A, B, and C afforded the bis-adduct 2 o’clock as mainly favored, albeit 12 o’clock also appears to be favored in some of them (criterion C for diethyl-malonate and criterion A for diisopropyl-malonate) (Figures S78 and S79). The latter might be reflected in the experimental finding of 12% ratios of 12 o’clock in both cases (Figure 5C). On the other hand, the MD study of mono-di-^*t*^Bu-malonate-C_70_⊂[10]CPP⊂**6**·(BArF)_8_ showed that in four out of five replicates the [10]CPP was placed vertically aligned inside the cavity of the nanocapsule, thus in a destabilizing mode caused by the bulkier ^*t*^Bu groups (Figure S80). Moreover, the MD analysis of the corresponding bis-adducts also points toward destabilizing conformations. Experimentally, we do observe very similar ratios among the three possible bis-adducts as if no supramolecular mask was present. We hypothesize that the reaction could take place at the gates or outside the cavity of the nanocapsule; therefore, the mask effect is lost. The latter is indeed supported by the observation of empty nanocapsules by HR-MS at the same voltage as where the other malonates are fully encapsulated.

## Conclusions

We have undertaken a comprehensive study of the use of supramolecular masks for taming the regioselectivity in the bis-Bingel functionalization of C_70_. Compared to bare C_70_, where ∼68% of the 2 o’clock bis-adduct for all bromomalonates explored is generally obtained, the use of nanocapsule **4**·(BArF)_8_ as a mask affords a remarkable switch of regioselectivity toward 5 o’clock over 2 o’clock. In the specific case of dibenzyl-bromomalonate (**Bn**), a 23:73 ratio of 2 o’clock:5 o’clock is achieved. To further restrict the exposed C_70_, the three-shell mask C_70_⊂[10]CPP⊂ **6**·(BArF)_8_ was synthesized. The **C**_**70**_**-Matryoshka** gave us perfect control of the polyfunctionalization, obtaining just the bis-adduct and the 2 o’clock as favored regioisomers. Again, in the case of dibenzyl-bromomalonate (**Bn**), a great match between the structure of the nanocapsule and the blockage of the [10]CPP and the addend strikingly led to the exclusive formation of the 2 o’clock bis-adduct as a pure regioisomer. MD simulations supported all experimental findings, providing us with a deep understanding of the dynamic behavior of mono- and bis-adducts inside the mask. Both the supramolecular mask and the interactions established by the monoadducts with the nanocapsule precisely control the dynamics and accessibility of the fullerene for bis-functionalization. This work demonstrates that the supramolecular mask strategy can also be a powerful tool to obtain chemo-, itero-, and regioselectivity in the less-explored functionalization of C_70_. By changing the type of mask and/or the type of addend, the regioselectivity can be tuned. This strategy could be useful for the challenging synthesis of isomer pure C_70_ adducts for future dedicated applications such as molecular electronics and solar cell design.

## Supporting Information Available

Materials, instrumentation, experimental procedures, spectroscopic and photophysical characterization of all compounds, and refs 22–36
  (PDF)
Mono-di-benzyl-C_70_⊂**4**·(BArF)_8_ (reactive orientation 1) (MPG)
Mono-di-benzyl-C_70_⊂**4**·(BArF)_8_ (reactive orientation 2 - most abundant) (MPG)
Mono-di-benzyl-C_70_⊂[10]CPP⊂**6**·(BArF)_8_ (reactive orientation 1 - most abundant) (MPG)
Mono-di-benzyl-C_70_⊂[10]CPP⊂**6**·(BArF)_8_ (reactive orientation 2) (MPG)
MD simulation of bis-dibenzyl-C_70_⊂[10]CPP⊂**6**·(BArF)_8_ (2 o’clock regio-isomer) (MOV)

## Supplementary Material

ja3c10808_si_001.pdf

ja3c10808_si_002.mpg

ja3c10808_si_003.mpg

ja3c10808_si_004.mpg

ja3c10808_si_005.mpg

ja3c10808_si_006.mov
