# Supplementary material for: Regioswitchable Bingel Bis-Functionalization of Fullerene C70 via Supramolecular Masks
Source: J Am Chem Soc. 2024 Feb 5;146(8):5186–94. doi: 10.1021/jacs.3c10808 (PMC10910506; doi:10.1021/jacs.3c10808)
Supplement: Supplementary file 1 — ja3c10808_si_001.pdf [file ja3c10808_si_001.pdf]

Supporting Information for:

Regio-switchable Bingel bis-functionalization of fullerene C_{70} via supramolecular masks

Valentina Iannace, Clara Sabrià, Youzhi Xu, Max von Delius, Inhar Imaz, Daniel Maspoch, Ferran Feixas and Xavi Ribas

Table of contents:

1. Materials and Instrumentations S3

2. Functionalization of Bare C_{70} S4

2.1 General procedure for statistical Bingel reaction (bare C_{70}) S4

2.2 Characterization of α,α-*bis*-C_{70}-adducts S5

2.2.1 α,α-*bis*-diethylmalonate-C_{70} S5

2.2.2 α,α-*bis*-diisopropylmalonate-C_{70} S7

2.2.3 α,α-*bis*-ditertbutylmalonate-C_{70} S9

2.2.4 α,α-*bis*-dibenzylmalonate-C_{70} S11

3. Supramolecular Mask Strategy S13

3.1 C_{70}⊂4·(BArF)$_8$ S13

3.1.1 Preparation of C_{70}⊂4·(BArF)$_8$ S13

3.1.2 General procedure for Bingel reaction (C_{70}⊂4·(BArF)$_8$ system) S13

3.1.3 Isolation of α,α-*bis*-malonate-C_{70}-adducts S14

3.1.4 Characterization of α,α-*bis*-malonate-C_{70}⊂4·(BArF)$_8$ S15

3.2 C_{70}⊂[10]CPP⊂6·(BArF)$_8$ S17

3.2.1 Preparation of C_{70}⊂[10]CPP⊂6·(BArF)$_8$ S17

3.2.2 General procedure for Bingel reaction S18

3.2.3 Isolation of α,α-*bis*-malonate- C_{70}⊂[10]CPP⊂6·(BArF)$_8$ S19

3.2.4 Characterization α,α-*bis*-malonate-C_{70}⊂[10]CPP⊂6·(BArF)$_8$ S20

3.3 C_{70}⊂[10]CPP⊂7·(BArF)$_8$ S22

3.3.2 Characterization of 7·(BArF)$_8$ and C_{70}⊂[10]CPP⊂[7·(BArF)$_8$] S22

3.4 Linear calibration for quantitative HPLC analysis S23

4. Supplementary Figures S26

4.1 LDI Mass Spectra S26

4.2 UV-Vis Spectra ... S28
4.3 NMR ... S28
4.4 HRMS Monitoring ... S40
4.4.1 C_{70}⊂4·(BArF)$_8$ ... S40
4.4.2 C_{70}⊂[10]CPP⊂6·(BArF)$_8$ ... S44
4.5 HRMS Spectra ... S48
5. X-Ray Diffraction ... S52
6. MD Simulations ... S53
6.1. System preparation and MD simulations protocols ... S53
6.2. MD simulations of mono- and bis-C_{70}⊂4·(BArF)$_8$ with dibenzyl-bromomalonate ... S54
6.3. MD simulations of mono- and bis-C_{70}⊂4·(BArF)$_8$ with diethyl-bromomalonate ... S55
6.4. MD simulations of mono- and bis-C_{70}⊂4·(BArF)$_8$ with diisopropyl-bromomalonate ... S56
6.5. MD simulations of mono- and bis-C_{70}⊂4·(BArF)$_8$ with ditertbutyl-bromomalonate ... S57
6.6. MD simulations of mono- and bis-C_{70}⊂[10]CPP⊂6·(BArF)$_8$ with dibenzyl-bromomalonate ... S58
6.7. MD simulations of mono- and bis-C_{70}⊂[10]CPP⊂6·(BArF)$_8$ with diethyl-bromomalonate ... S60
6.8. MD simulations of mono- and bis-C_{70}⊂[10]CPP⊂6·(BArF)$_8$ with diisopropyl-bromomalonate ... S62
6.9. MD simulations of mono- and bis-C_{70}⊂[10]CPP⊂6·(BArF)$_8$ with ditertbutyl-bromomalonate ... S64
7. Supporting Videos ... S65
8. References ... S66

1. Materials and Instrumentations

Reagents and solvents used were commercially available reagent quality unless indicated otherwise. The synthesis of the nanocapsules has been performed following the reported procedures for 4·(BArF)$_8$ 6·(BArF)$_8$ 7·(BArF)$_8$.[1-2]

C_{70}⊂[10]CPP complex was prepared following a similar procedure published for C_{60} ⊂ [10]CPP.[2] Bromomalonates were prepared according to a published procedure.[3] Characterization data was consistent with the previous report.

NMR data concerning product characterization were collected on Bruker 400 MHz AVANCE spectrometers in $CDCl_3$ or CD_3CN, and calibrated relative to the residual protons of the solvent. ESI-MS experiments were collected and analysed on Bruker MicroTOF-Q-II using CH_3CN as a mobile phase. MALDI-TOF-MS analysis were collected on an Autoflex maX Bruker Daltonics with a Bruker Smartbeam II (355 nm wavelength) without matrix. HPLC data concerning fullerene adducts identity were collected on Agilent Technologies LC 1200 series instrument equipped with a Cosmosil Buckyprep-M column (4.6 mm I.D. x 250 mm, particle size: 5 µm, Nacalai Tesque, Inc.) and using toluene as solvent (1 ml/min flow), monitored with a diode array UV-Vis detector at 320 nm to observe the UV-Vis spectra characteristics of each bis-C_{70}-adduct regioisomer. X-ray diffraction data were collected on both Bruker D8 QUEST ECO and ALBA synchrotron.

Molecular Dynamic (MD) Simulations were carried out using the AMBER 16 package.[4] Each guest molecule were parametrized using the standard protocols within the GAFF force-field.[5] The restricted electrostatic potential atomic charges are obtained from B3LYP/6-31G* calculations. Each host-guest system was immersed in a preequilibrated truncated octahedron box with a 10 Å buffer of acetonitrile molecules using the leap module. Then, each host-guest system was submitted to a two-stage geometry optimization approach. The first stage corresponds to a minimization of the positions of solvent molecules and ions imposing positional restraints on the solute by harmonic potential with a force constant of 500 kcal mol-1 Å-2. In the second stage, an unrestrained minimization of all the atoms in the simulation cell was performed. Then, the system was heated using six 50 ps steps, where the temperature is incremented 50 K each step (0-300 K) under constant volume and periodic boundary conditions. The time step was maintained at 2 fs during all the heating stages, allowing potential in homogeneities to self-adjust. Next, each system was equilibrated without restrains for 2 ns with a 2 fs time step at a constant pressure of 1 atm and a temperature of 300 K. Once the equilibration stage is completed the system is ready for long production runs. With these parameters, MD simulations have been performed of each system, starting with the fullerene inside the cavity of each respective cage. To obtain more accurate results usually more than one replicate is needed. In this case, 5 replicates of 0,5 microseconds (µs) for each system have been simulated.

2. Functionalization of Bare C_{70}

2.1 General procedure for statistical Bingel reaction (bare C_{70})

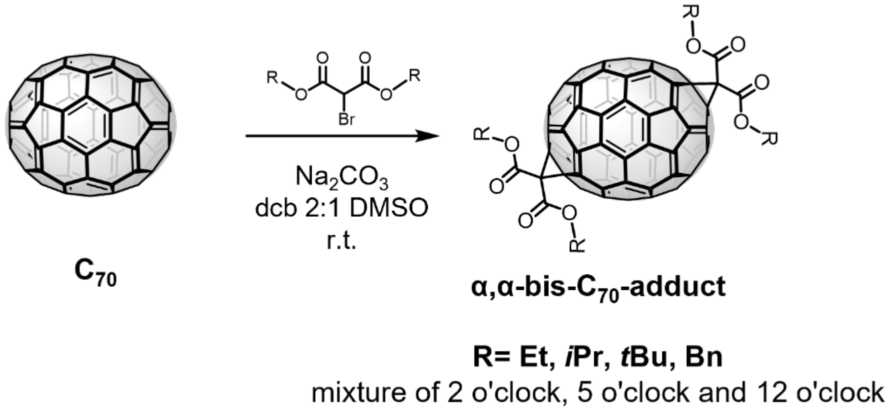

Figure S1. Procedure for the Bingel reaction on Bare C_{70}.

<u>Preparation of α,α-*bis*-x-C_{70}-adducts (x = **Et** (-diethyl), ***i*Pr** (-diisopropyl), ***t*Bu** (-tertbutyl), **Bn** (-dibenzyl):</u>

8.4 mg of C_{70} (1.0 mmol) were dissolved in 500 µl of o-DCB, then 1.5 µl of bromomalonate (0.75 mmols) was added. At the same time, 4.8 mg of Na_2CO_3 (4.0 mmols) were dissolved in 250 µl of DMSO and added to the first solution. A DMSO:o-DCB ratio 1:2 was used. The reaction took place in a few minutes (20-60 min) leading to a mixture of multiple adducts of C_{70}, bis adducts were isolated firstly by chromatographic column (silica gel, Toluene 100%), then the regioisomers have been separated by preparative TLC (silica, Toluene 100%). The isolated yield for bis-adducts was around 5%.

The products were characterized by means of MALDI-TOF MS, HPLC, UV-Vis, and ^{1}H NMR and in some cases ^{13}C NMR.

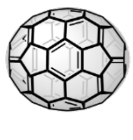

		12 o'clock	2 o'clock	5 o'clock
2-bromo malonates	Et	19%	68%	13%
	*i*Pr	14%	68%	18%
	*t*Bu	16%	68%	14%
	Bn	21%	69%	10%

Figure S2. Regioisomers ratio obtained for the Bingel reaction on Bare C_{70} with the four different 2-bromo-malonates. (**Et** (-diethyl), ***i*Pr** (-diisopropyl), ***t*Bu** (-tertbutyl), **Bn** (-dibenzyl))

2.2 Characterization of α,α-*bis*-C_{70}-adducts

2.2.1 α,α-*bis*-diethylmalonate-C_{70}

α,α-*bis*-diethylmalonate-C_{70} (2 o'clock regioisomer)

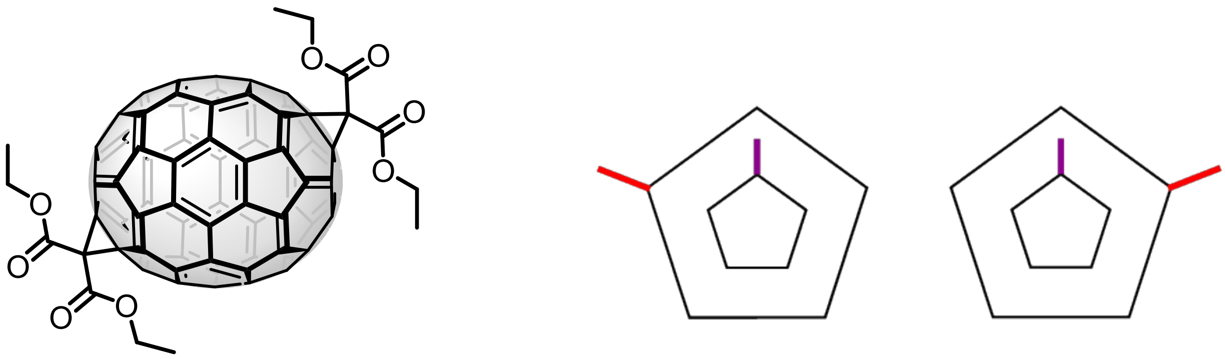

Figure S3. α,α-*bis*-diethylmalonate-C_{70} (2 o'clock regioisomer), Schlegel Diagram representing one functionalized bond in red and the other one in purple.

<u>LDI Mass Spectra</u>

Calculated: 1156.1153 and found 1156.650. (See **Figure S32**)

<u>UV-VIS Spectra</u>

UV-Vis Spectra shows maximum in absorption at 397 nm, 433 nm, 465 nm, shoulder at 527 nm. (See **Figure S36a**)

<u>NMR Spectra</u>

^{1}H NMR (400 MHz, $CDCl_3$) δ p.p.m.: δ 4.52 (td, J = 7.1, 1.8 Hz, 8H), 1.48 (q, J = 7.1 Hz, 12H). (See **Figure S38**)

^{13}C NMR (100 MHz, $CDCl_3$) δ p.p.m.: δ 163.73 (C=O), 163.63 (C=O), 155.72 - 131.27 (C_{70}), 63.61 (CH_2), 63.58 (CH_2), 14.40 (CH_3). (See **Figure S39**)

α,α-*bis*-diethylmalonate-C_{70} (5 o'clock regioisomer)

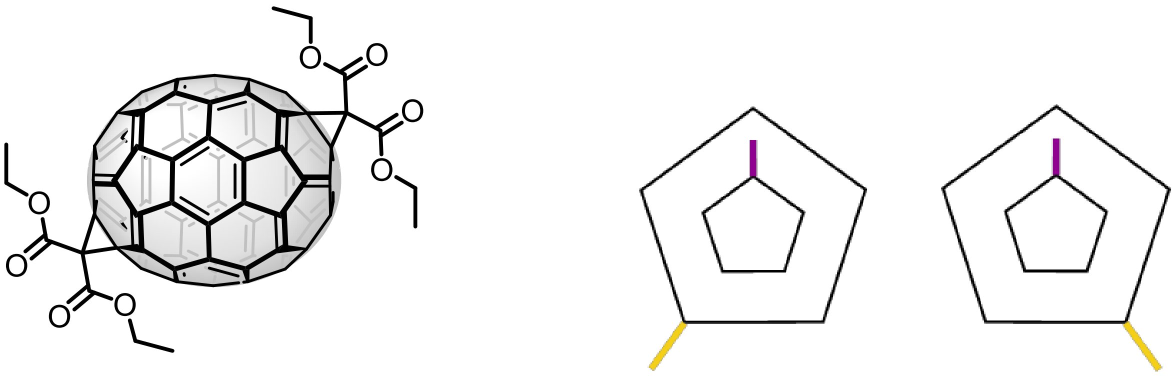

Figure S4. α,α-*bis*-diethylmalonate-C_{70} (5 o'clock regioisomer), Schlegel Diagram representing one functionalized bond in yellow and the other one in purple.

<u>LDI Mass Spectra</u>

Calculated: 1156.1153 and found 1156.650. (See **Figure S32**)

UV-VIS Spectra

UV-Vis Spectra shows maximum in absorption at 475 nm. (See **Figure S36b**)

NMR Spectra

^{1}H NMR (400 MHz, $CDCl_3$) δ p.p.m.: δ 4.54 – 4.47 (m, 8H), 1.47 (td, J = 7.1, 4.2 Hz, 12H). (See **Figure S40**)

^{13}C NMR (100 MHz, $CDCl_3$) δ p.p.m.: δ 163.55 (C=O); 155.42 – 133.00 (C_{70}); 63.54 (CH_2), 14.37 (CH_3). (See **Figure S41**)

α,α-*bis*-diethylmalonate-C_{70} (12 o'clock regioisomer)

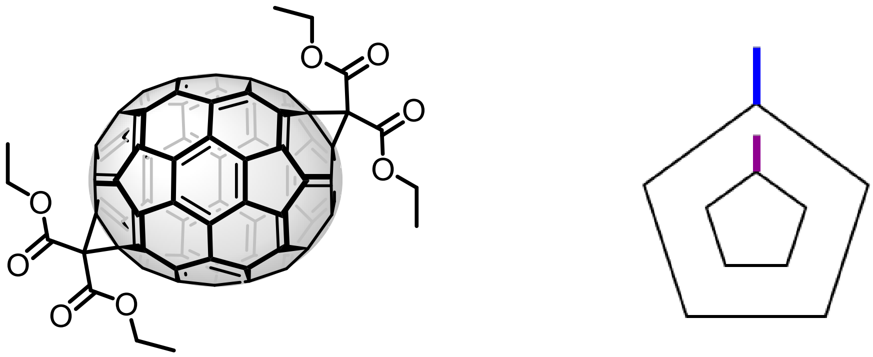

Figure S5. α,α-*bis*-diethylmalonate-C_{70} (12 o'clock regioisomer), Schlegel Diagram representing one functionalized bond in blue and the other one in purple.

LDI Mass Spectra

Calculated: 1156.1153 and found 1156.650. (See **Figure S32**)

UV-VIS Spectra

UV-Vis Spectra shows maximum in absorption at 403 nm and 481 nm. (See **Figure S36c**)

NMR Spectra

^{1}H NMR (400 MHz, $CDCl_3$) δ p.p.m.: δ 4.53 (qd, J = 7.1, 1.6 Hz, 8H), 1.49 (t, J = 7.1 Hz, 12H). (See **Figure S42**)

^{13}C NMR 100 MHz, $CDCl_3$) δ p.p.m.: δ 163.7 (C=O) 154.76-130.97 (C_{70}), 63.55 (CH_2), 14.40 (CH_3). (See **Figure S43**)

2.2.2 α,α-*bis*-diisopropylmalonate-C_{70}

α,α-*bis*-diisopropylmalonate-C_{70} (2 o'clock regioisomer)

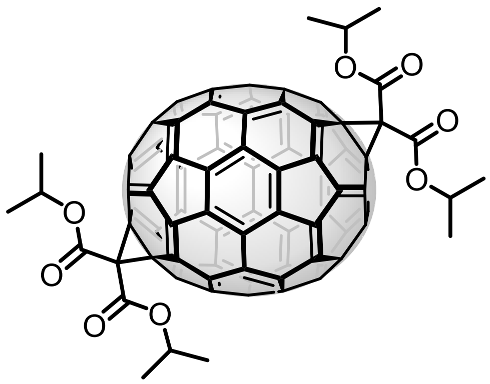

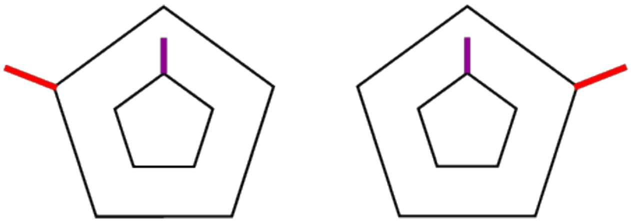

Figure S6. α,α-*bis*-diisopropylmalonate-C_{70} (2 o'clock regioisomer), Schlegel Diagram representing one functionalized bond in red and the other one in purple.

<u>LDI Mass Spectra</u>

Calculated: 1212.1770 and found 1212.682. (See **Figure S33**)

<u>UV-Vis Spectra</u>

UV-Vis Spectra shows maximum in absorption at 397 nm, 433 nm, 465 nm, shoulder at 527 nm. (See **Figure S36a**)

<u>NMR Spectra</u>

^{1}H NMR (400 MHz, $CDCl_3$) δ p.p.m.: δ 5.35 (dq, J = 12.8, 6.4 Hz, 1H), 1.52 – 1.40 (m, 6H). (See **Figure S44**)

α,α-*bis*-diisopropylmalonate-C_{70} (5 o'clock regioisomer)

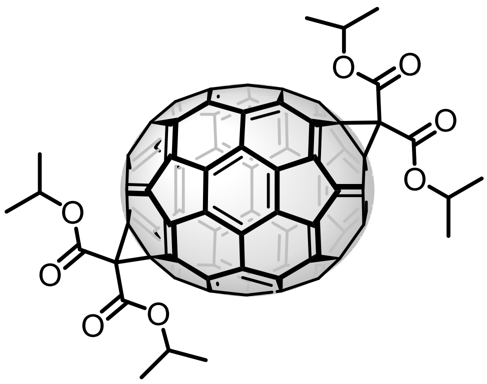

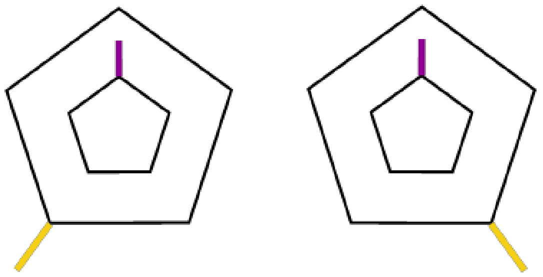

Figure S7. α,α-*bis*-diisopropylmalonate-C_{70} (5 o'clock regioisomer), Schlegel Diagram representing one functionalized bond in yellow and the other one in purple.

<u>LDI Mass Spectra</u>

Calculated: 1212.1770 and found 1212.682. (See **Figure S33**)

UV-Vis Spectra

UV-Vis Spectra shows maximum in absorption at 475 nm. (See **Figure S36b**)

NMR Spectra

^{1}H NMR (400 MHz, $CDCl_3$) δ p.p.m.:) δ 5.34 (hept, *J* = 6.2 Hz, 1H), 1.45 (ddd, *J* = 21.5, 6.3, 4.3 Hz, 6H). (See **Figure S45**)

α,α-*bis*-diisopropylmalonate-C_{70} (12 o'clock regioisomer)

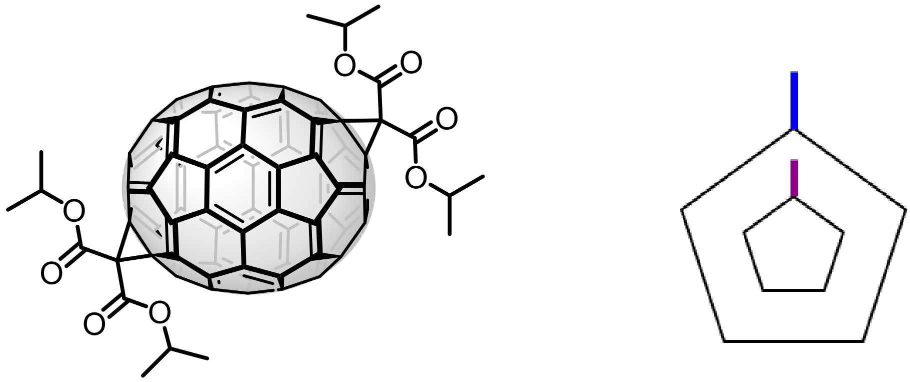

Figure S8. α,α-*bis*-diisopropylmalonate-C_{70} (12 o'clock regioisomer), Schlegel Diagram representing one functionalized bond in blue and the other one in purple.

LDI Mass Spectra

Calculated: 1212.1770 and found 1212.682. (See **Figure S33**)

UV-Vis Spectra

UV-Vis Spectra shows maximum in absorption at 403 nm and 481 nm. (See **Figure S36c**)

NMR Spectra

^{1}H NMR (400 MHz, $CDCl_3$) δ p.p.m.: δ 5.36 (hept, *J* = 6.2 Hz, 1H), 1.47 (dd, *J* = 16.1, 6.3 Hz, 6H). (See **Figure S46**)

2.2.3 α,α-*bis*-ditertbutylmalonate-C_{70}

α,α-*bis*-ditertbutylmalonate-C_{70} (2 o'clock regioisomer)

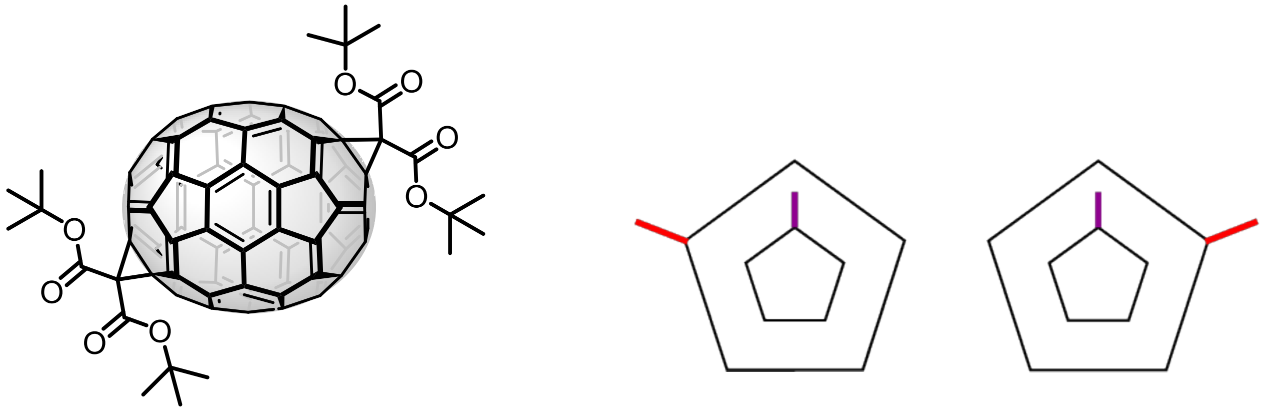

Figure S9. α,α-*bis*-ditertbutylmalonate-C_{70} (2 o'clock regioisomer), Schlegel Diagram representing one functionalized bond in red and the other one in purple.

<u>LDI Mass Spectra</u>

Calculated: 1268.2405 and found 1268.842. (See **Figure S34**)

<u>UV-Vis Spectra</u>

UV-Vis Spectra shows maximum in absorption at 397 nm, 433 nm, 465 nm, shoulder at 527 nm. (See **Figure S36a**)

<u>NMR Spectra</u>

^{1}H NMR (400 MHz, $CDCl_3$) δ p.p.m.: δ 1.69 (s, 18H), 1.67 (s, 18H). (See **Figure S47**)

α,α-*bis*-ditertbutylmalonate-C_{70} (5 o'clock regioisomer)

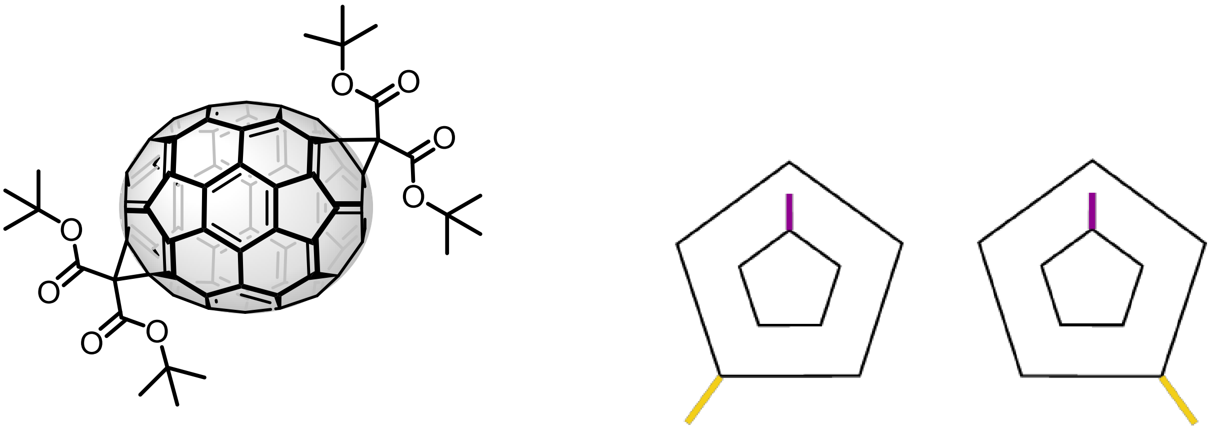

Figure S10. α,α-*bis*-ditertbutylmalonate-C_{70} (5 o'clock regioisomer), Schlegel Diagram representing one functionalized bond in yellow and the other one in purple.

<u>LDI Mass Spectra</u>

Calculated: 1268.2405 and found 1268.842. (See **Figure S34**)

UV-Vis Spectra

UV-Vis Spectra shows maximum in absorption at 475 nm. (See **Figure S36b**)

NMR Spectra

^{1}H NMR (400 MHz, $CDCl_3$) δ p.p.m.: δ 1.69 (s, 18H), 1.68 (s, 18H). (See **Figure S48**)

α,α-*bis*-ditertbutylmalonate-C_{70} (5 o'clock regioisomer)

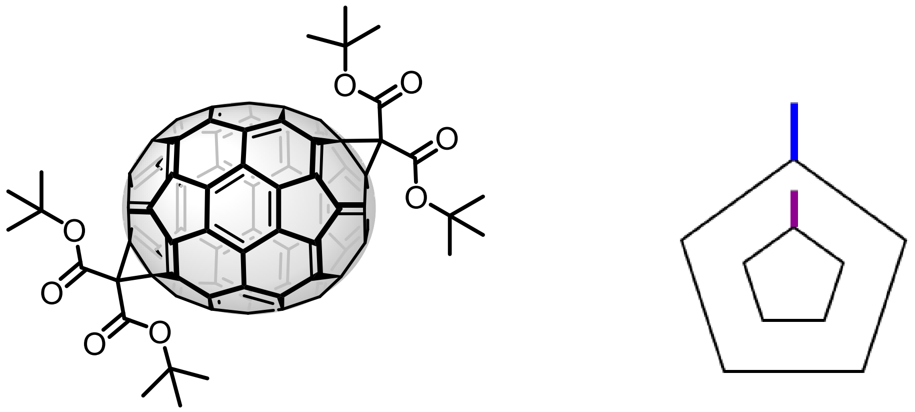

Figure S11. α,α-*bis*-ditertbutylmalonate-C_{70} (12 o'clock regioisomer), Schlegel Diagram representing one functionalized bond in blue and the other one in purple.

LDI Mass Spectra

Calculated: 1268.2405 and found 1268.842. (See **Figure S34**)

UV-Vis Spectra

UV-Vis Spectra shows maximum in absorption at 403 nm and 481 nm. (See **Figure S36c**)

NMR Spectra

^{1}H NMR (400 MHz, $CDCl_3$) δ p.p.m.: δ 1.68 (s, 36H). (See **Figure S49**)

2.2.4 α,α-*bis*-dibenzylmalonate-C_{70}

α,α-*bis*-dibenzylmalonate-C_{70} (2 o'clock regioisomer)

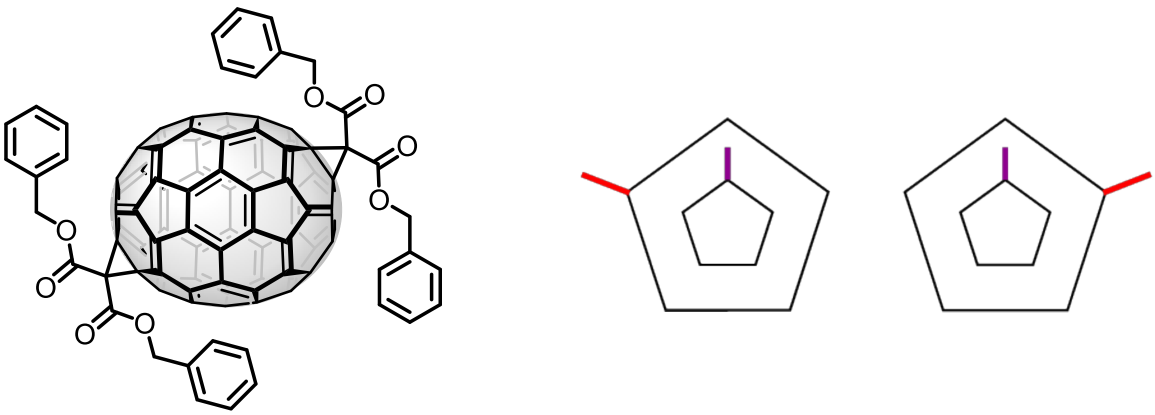

Figure S12. α,α-*bis*-dibenzylmalonate-C_{70} (2 o'clock regioisomer), Schlegel Diagram representing one functionalized bond in red and the other one in purple.

<u>LDI Mass Spectra</u>

Calculated: 1404.1779 and found 1404.861. (See **Figure S35**)

<u>UV-Vis Spectra</u>

UV-Vis Spectra shows maximum in absorption at 397 nm, 433 nm, 465 nm, shoulder at 527 nm. (See **Figure S36a**)

<u>NMR Spectra</u>

^{1}H NMR (400 MHz, $CDCl_3$) δ p.p.m.: δ 7.45 – 7.34 (m, 20H), 5.42 (d, J = 8.1 Hz, 8H). (See **Figure S50**)

^{13}C NMR (100 MHz, $CDCl_3$) δ p.p.m.: δ 163.46 (C=O), 163.37 (C=O), 155.62 – 131.51 (C_{70}), 129.06 – 128.87 (C-Ar), 69.13 (CH_2). (See **Figure S51**)

α,α-*bis*-dibenzylmalonate-C_{70} (5 o'clock regioisomer)

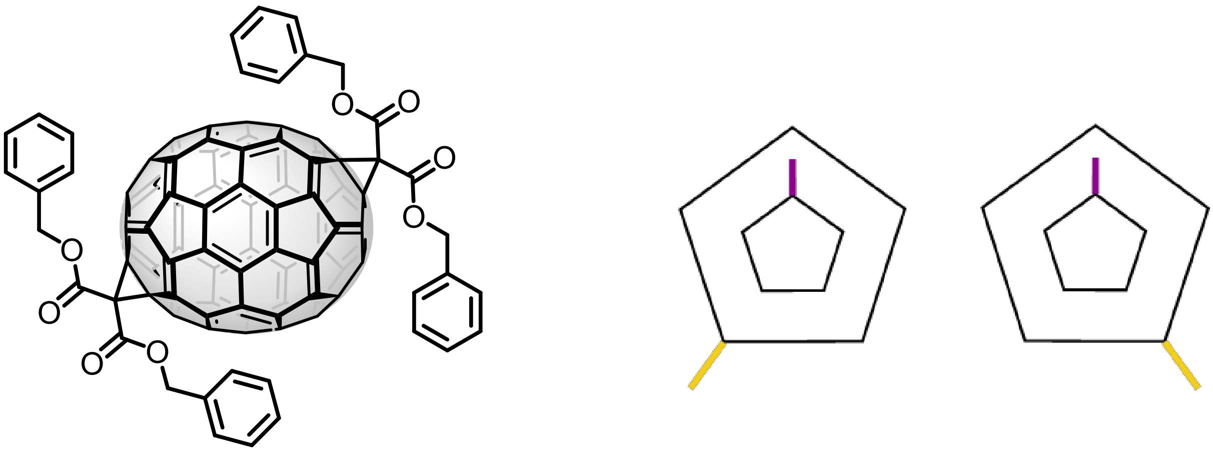

Figure S13. α,α-*bis*-dibenzylmalonate-C_{70} (5 o'clock regioisomer), Schlegel Diagram representing one functionalized bond in yellow and the other one in purple.

LDI Mass Spectra

Calculated: 1404.1779 and found 1404.861. (See **Figure S35**)

UV-Vis Spectra

UV-Vis Spectra shows maximum in absorption at 475 nm. (See **Figure S36b**)

NMR Spectra

^{1}H NMR (400 MHz, $CDCl_3$) δ p.p.m.: δ 7.42 – 7.35 (m, 20H), 5.39 (d, *J* = 6.6 Hz, 8H). (See **Figure S52**)

α,α-*bis*-dibenzylmalonate-C_{70} (12 o'clock regioisomer)

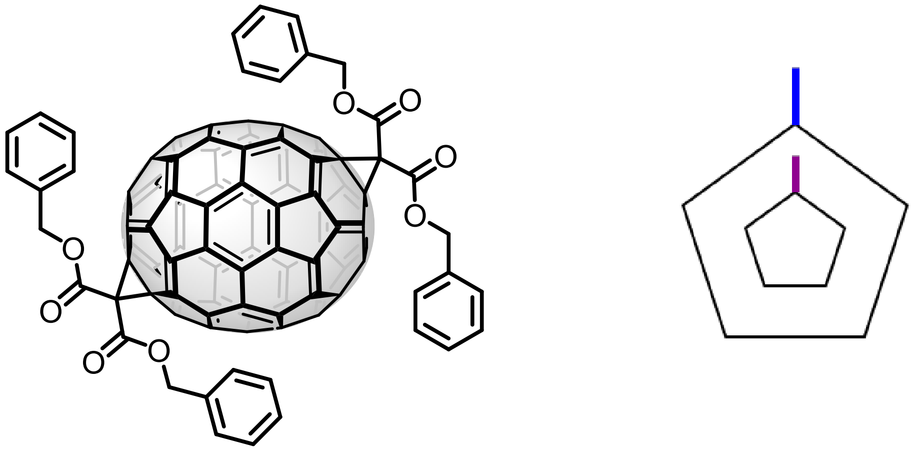

Figure S14. α,α-*bis*-dibenzylmalonate-C_{70} (12 o'clock regioisomer), Schlegel Diagram representing one functionalized bond in blue and the other one in purple.

LDI Mass Spectra

Calculated: 1404.1779 and found 1404.861. (See **Figure S35**)

UV-Vis Spectra

UV-Vis Spectra shows maximum in absorption at 403 nm and 481 nm. (See **Figure S36c**)

NMR Spectra

^{1}H NMR (400 MHz, $CDCl_3$) δ p.p.m.: ^{1}H NMR (400 MHz, $CDCl_3$) δ 7.44 – 7.34 (m, 20H), 5.42 (s, broad 8H). (See **Figure S53**)

3. Supramolecular Mask Strategy

3.1 C_{70}⊂4·(BArF)$_8$

3.1.1 Preparation of C_{70}⊂4·(BArF)$_8$

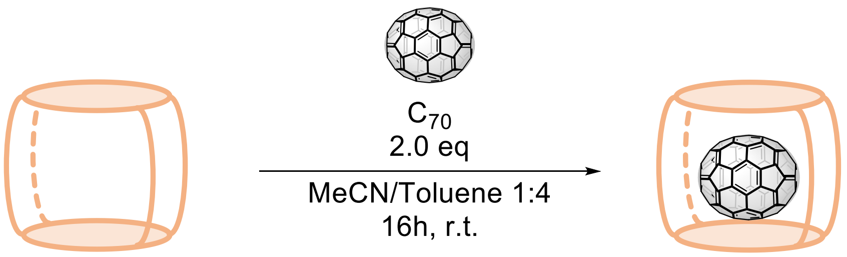

4·(BArF)$_8$
1.0 eq

C_{70} 4·(BArF)$_8$

Figure S15. Synthesis of C_{70}⊂**4**·(BArF)$_8$ host-guest complex.

15 mg of 4·(BArF)$_8$ nanocapsule (1 eq) are dissolved with CH_3CN (1 mL). Then 2 eq of C_{70} (2.0 mg) dissolved in Toluene (4 mL) are added to the nanocapsule solution and stirred at room temperature for 16 h. After this time the solvent is removed under reduced pressure, MeCN is added to solubilize the product filtering to eliminate the excess of C_{70} (that can be recovered adding $CHCl_3$.).

<u>C_{70}⊂4·(BArF)$_8$ HRMS (m/z)</u> (See **Figure S62**):

calculated 747.4130 and found 747.4108 (C_{70}⊂[**4**·(BArF)$_0$]$^{+8}$);

calculated 977.6245 and found 977.6202 (C_{70} ⊂[**4**·(BArF)$_1$]$^{+7}$);

calculated 1284.4064 and found 1284.3987 (C_{70}⊂[**4**·(BArF)$_2$]$^{+6}$);

calculated 1713.9012 and found 1713.8888 (C_{70}⊂[**4**·(BArF)$_3$]$^{+5}$);

calculated 2358.1433 and found 2358.1193 (C_{70}⊂[**4**·(BArF)$_4$]$^{+4}$).

3.1.2 General procedure for Bingel reaction (C_{70}⊂4·(BArF)$_8$ system)

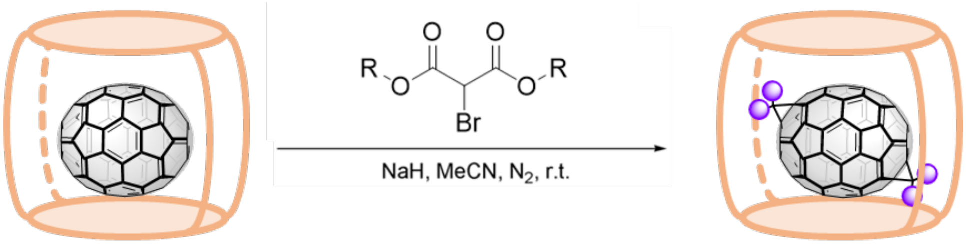

Figure S16. General procedure for Bingel reaction via supramolecular mask strategy on C_{70}⊂**4**·(BArF)$_8$ (R= -ethyl, -isopropyl, -tertbutyl, -benzyl.)

15 mg of C_{70}⊂4·(BArF)$_8$ were totally dissolved in 0.5 ml of CH_3CN. Then, x equiv. (x= 3-4 see below) of the corresponding bromomalonate (diethyl; diisopropyl; ditertbutyl; dibenzyl) and x equiv. (x= 3-4 see below) of NaH as a base were added sequentially and the reaction was stirred under N_2 at room temperature. Once the bis-adduct was formed (monitored by HRMS), the reaction was stopped by

filtering and removing the solvent under reduced pressure. The equivalents of NaH and 2-bromo malonate used are in a range of x= 3-4 with addition of 1 equivalent per hour in 3-4 hours.

To see the HRMS monitoring of each reaction go to **Figure S54** (**Et**), **Figure S55** (***i*Pr**), **Figure S56** (***t*Bu**), **Figure S57** (**Bn**).

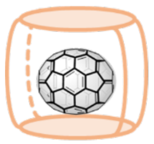

2-bromo malonates	12 o'clock	2 o'clock	5 o'clock
**Et**	2%	40%	58%
***i*Pr**	6%	51%	43%
***t*Bu**	20%	63%	17%
**Bn**	**4%**	**23%**	**73%**

Figure S17. Regioisomers ratio obtained for the Bingel reaction on C_{70}⊂**4**·$(BArF)_8$ with the four different 2-bromo-malonates. (**Et** (-diethyl), ***i*Pr** (-diisopropyl), ***t*Bu** (-tertbutyl), **Bn** (-dibenzyl))

3.1.3 Isolation of α,α-*bis*-malonate-C_{70}-adducts

The crude α,α-bis-dibenzylmalonate-C_{70}⊂**4**·$(BArF)_8$ reaction mixture was washed with $CHCl_3$ affording a precipitate. The precipitate was dissolved in CH_3CN and an excess of TfOH (20 equiv.) was added to afford the disassembly of the nano capsule. The mixture has been stirred at room temperature during 30 min, then, the solution was dried with N_2 gas flow and the α,α-bis-C_{70}-adducts were obtained after $CHCl_3$ addition and filtration of the solid. The three regioisomers were separated by preparative TLC (silica) in Toluene 100%. Each regioisomer has been characterized by HPLC and UV-Vis to confirm the identity, which was also cross-matched with an independently synthesized sample of the regioisomer. The yield of the reaction was calculated by HPLC using C_{60} as internal standard (taking into account the calibration factor calculated in section 3.4). The yield obtained is in the range of 13-40% depending on the malonate used (diethyl= 13%; diisopropyl= 40%; ditertbutyl= 26% ; dibenzyl= 31%).

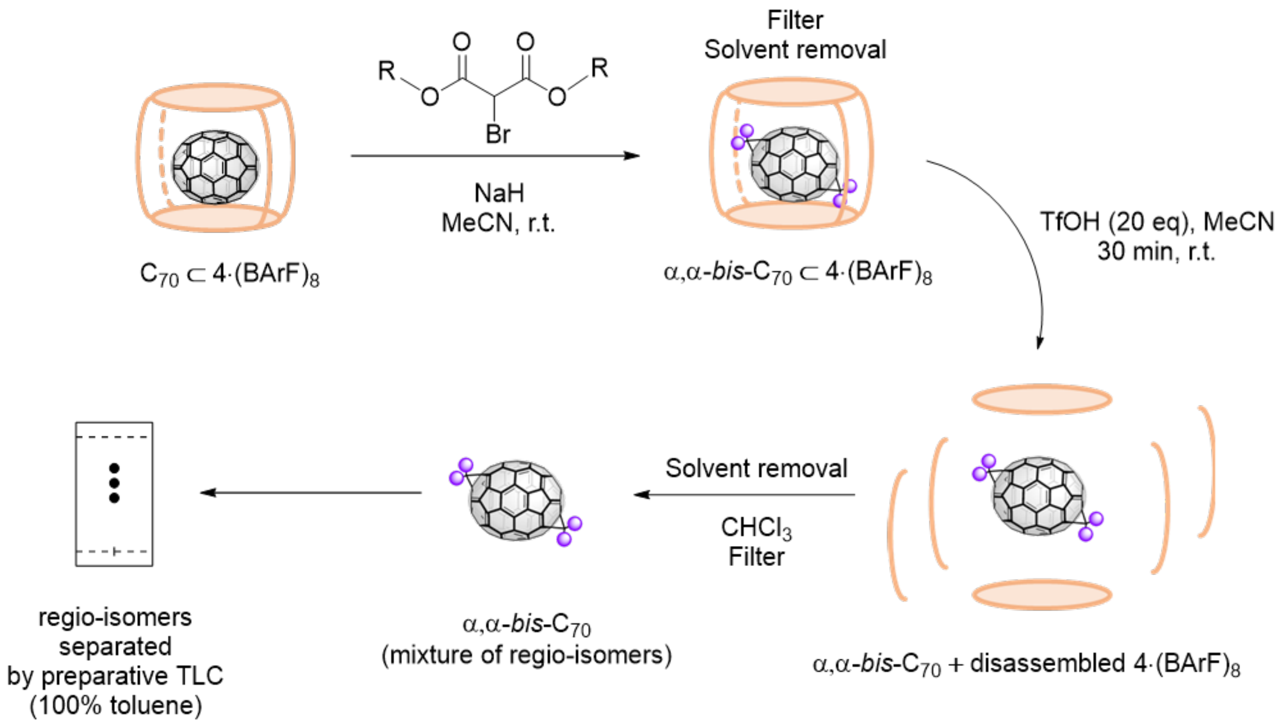

Figure S18. Detailed work-up flow for the isolation of α,α-*bis*-C_{70} -adducts obtained from Bingel reaction with C_{70}⊂**4**·$(BArF)_8$ system.

3.1.4 Characterization of α,α-*bis*-malonate-C_{70}⊂4·$(BArF)_8$

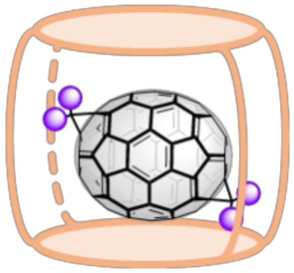

Figure S19. α,α-*bis*-malonate-C_{70}⊂**4**·$(BArF)_8$

The formation of the α,α-*bis*-malonate-C_{70} -adducts inside the nanocapsule **4**·$(BArF)_8$ is confirmed by HRMS, taking into account that the three regioisomers obtained with one 2-bromo malonate (2 o'clock, 5 o'clock and 12 o'clock) present the same mass.

α,α-*bis*-diethylmalonate-C_{70}⊂4·$(BArF)_8$ HRMS (m/z) (See **Figure S63**):

calculated 787.0526 and found 787.0526 (α,α-*bis*-diethylmalonate-C_{70}⊂[**4**·$(BArF)_0]^{+8}$);

calculated 1022.7840 and found 1022.7829 (α,α-*bis*-diethylmalonate-C_{70} ⊂[**4**·$(BArF)_1]^{+7}$);

calculated 1337.0925 and found 1337.0907 (α,α-*bis*-diethylmalonate-C_{70}⊂[**4**·$(BArF)_2]^{+6}$);

calculated 1777.1244 and found 1777.0709 (α,α-*bis*-diethylmalonate-C_{70}⊂[**4**·$(BArF)_3]^{+5}$);

calculated 2437.1724 and found 2437.2241 (α,α-*bis*-diethylmalonate-C_{70}⊂[**4**·$(BArF)_4]^{+4}$).

<u>α,α-*bis*-diisopropylmalonate-C_{70}⊂4·$(BArF)_8$</u> HRMS (m/z) (See **Figure S64**):

calculated 794.0604 and found 794.0426 (α,α-*bis*-diisopropylmalonate-C_{70}⊂[**4**·$(BArF)_0]^{+8}$);

calculated 1030.7929 and found 1030.7929 (α,α-*bis*-diisopropylmalonate-C_{70} ⊂[**4**·$(BArF)_1]^{+7}$);

calculated 1346.4363 and found 1346.4199 (α,α-*bis*-diisopropylmalonate-C_{70}⊂[**4**·$(BArF)_2]^{+6}$);

calculated 1788.3370 and found 1788.2841 (α,α-*bis*-diisopropylmalonate-C_{70}⊂[**4**·$(BArF)_3]^{+5}$);

calculated 2451.1880 and found 2450.8776 (α,α-*bis*-diisopropylmalonate-C_{70}⊂[**4**·$(BArF)_4]^{+4}$).

<u>α,α-*bis*-ditertbutylmalonate-C_{70}⊂4·$(BArF)_8$</u> HRMS (m/z) (See **Figure S65**):

calculated 801.0682 and found 801.0415 (α,α-*bis*-ditertbutylmalonate-C_{70}⊂[**4**·$(BArF)_0]^{+8}$);

calculated 1038.8019 and found 1038.7696 (α,α-*bis*-ditertbutylmalonate-C_{70} ⊂[**4**·$(BArF)_1]^{+7}$);

calculated 1355.7801 and found 1355.7341 (α,α-*bis*-ditertbutylmalonate-C_{70}⊂[**4**·$(BArF)_2]^{+6}$);

calculated 1799.5495 and found 1799.4843 (α,α-*bis*-ditertbutylmalonate-C_{70}⊂[**4**·$(BArF)_3]^{+5}$);

calculated 2465.2037 and found 2465.2887 (α,α-*bis*-ditertbutylmalonate-C_{70}⊂[**4**·$(BArF)_4]^{+4}$).

<u>α,α-*bis*-dibenzylmalonate-C_{70}⊂4·$(BArF)_8$</u> HRMS (m/z) (See **Figure S66**):

calculated 818.0605 and found 818.0605 (α,α-*bis*-dibenzylmalonate-C_{70}⊂[**4**·$(BArF)_0]^{+8}$);

calculated 1058.2216 and found 1058.2030 (α,α-*bis*-dibenzylmalonate-C_{70} ⊂[**4**·$(BArF)_1]^{+7}$);

calculated 1378.4364 and found 1378.4246 (α,α-*bis*-dibenzylmalonate-C_{70}⊂[**4**·$(BArF)_2]^{+6}$);

calculated 1826.7371 and found 1826.6959 (α,α-*bis*-dibenzylmalonate-C_{70}⊂[**4**·$(BArF)_3]^{+5}$);

calculated 2499.1882 and found 2499.1882 (α,α-*bis*-dibenzylmalonate-C_{70}⊂[**4**·$(BArF)_4]^{+4}$).

3.2 $C_{70} \subset [10]CPP \subset 6 \cdot (BArF)_8$

3.2.1 Preparation of $C_{70} \subset [10]CPP \subset 6 \cdot (BArF)_8$

Synthesis of $C_{70} \subset [10]CPP$

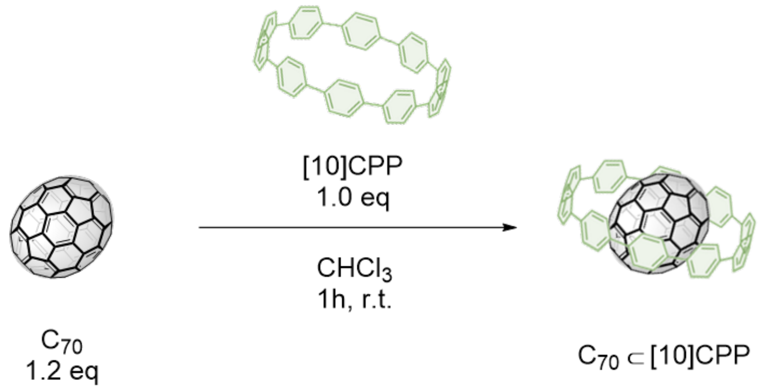

Figure S20. Encapsulation of C_{70} in [10]CPP ring.

A glass vial was charged with C_{70} (5mg, 1.2 eq), [10]CPP (6.6 mg, 1 eq) and 25 ml of $CHCl_3$. The reaction mixture was stirred at room temperature for 1 hour. After this time, solvent was removed under reduced pressure and toluene was added and the excess of C_{70} was removed by centrifugation. This procedure was repeated several times until the toluene was not coloured anymore.

Characterization:

^{1}H-NMR (400 MHz, $CDCl_3$) δ p.p.m.: 7.47 (s, 40H, arom). (See **Figure S37**)

Synthesis of $C_{70} \subset [10]CPP \subset 6 \cdot (BArF)_8$

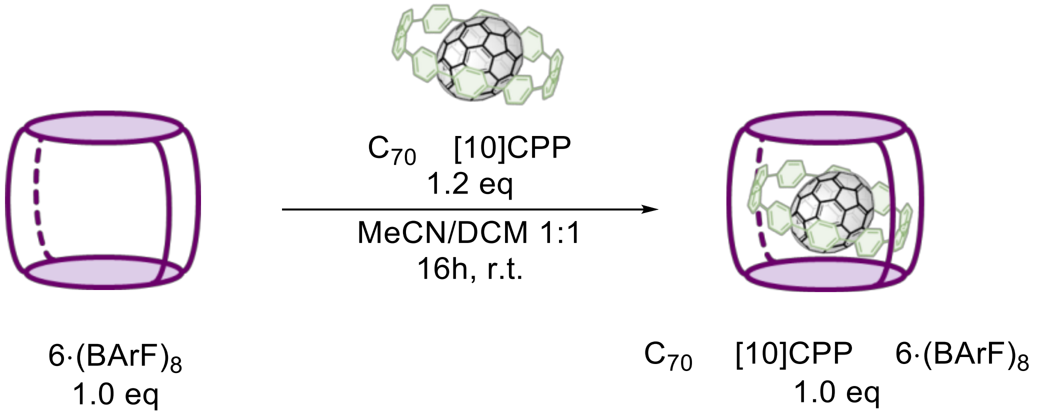

Figure S21. Synthesis of the Matryoshka-like complex $C_{70} \subset [10]CPP \subset$ **6**$\cdot (BArF)_8$.

15 mg of **6**$\cdot (BArF)_8$ nanocapsule (1 eq) are dissolved with the minimum amount of CH_3CN (1 mL). Then 1.2 eq of $C_{70} \subset [10]CPP$ (2.35 mg) dissolved in DCM are added to the nanocapsule solution and stirred at room temperature for 16 h. After this time the solvent is removed under reduced pressure,

MeCN is add to solubilize the product filtering to eliminate the excess of C_{70}⊂[10]CPP (that can be recovered adding $CHCl_3$.).

Characterization:

6·$(BArF)_8$ HRMS (m/z) (See **Figure S67**):

calculated 666.4128 and found 666.3969 ([**6**·$(BArF)_0]^{+8}$);

calculated 884.9099 and found 884.8879 ([**6**·$(BArF)_1]^{+7}$);

calculated 1176.2394 and found 1176.2067 ([**6**·$(BArF)_2]^{+6}$);

calculated 1584.1008 and found 1584.0532 ([**6**·$(BArF)_3]^{+5}$);

calculated 2195.8928 and found 2195.8176 ([**6**·$(BArF)_4]^{+4}$).

C_{70}⊂[10]CPP⊂**6**·$(BArF)_8$ HRMS (m/z) (See **Figure S67**):

calculated 866.5775 and found 866.5660 (C_{70}⊂[10]CPP⊂[**6**·$(BArF)_0]^{+8}$);

calculated 1113.6696 and found 1113.6539 (C_{70}⊂[10]CPP⊂[**6**·$(BArF)_1]^{+7}$);

calculated 1443.2924 and found 1443.1051 (C_{70}⊂[10]CPP⊂[**6**·$(BArF)_2]^{+6}$);

calculated 1904.5643 and found 1904.5393 (C_{70}⊂[10]CPP⊂[**6**·$(BArF)_3]^{+5}$);

calculated 2596.4722 and found 2596.1868 (C_{70}⊂[10]CPP⊂[**6**·$(BArF)_4]^{+4}$).

3.2.2 General procedure for Bingel reaction

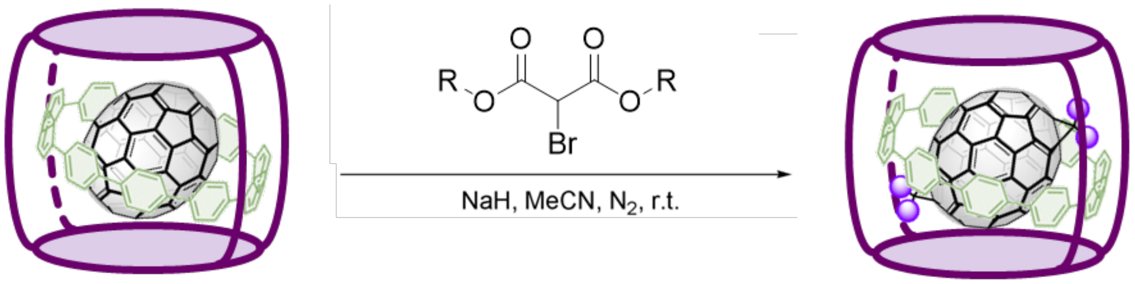

Figure S22. General procedure for Bingel reaction via supramolecular mask strategy on C_{70}⊂[10]CPP⊂**6**·$(BArF)_8$ (R= -ethyl, -isopropyl, -tertbutyl, -benzyl.)

15 mg of C_{70}⊂[10]CPP⊂**6**·$(BArF)_8$ were totally dissolved in 0.5 ml of CH_3CN. Then, x equiv. (x = 8-10, see below) of the corresponding bromomalonate (diethyl; diisopropyl; ditertbutyl; dibenzyl) and x equiv. (x = 8-10, see below) of NaH as a base were added sequentially and the reaction was stirred under N_2 at room temperature. Once the bis-adducts were completely formed (monitored by HRMS), the reaction was stopped by filtering and removing the solvent under reduced pressure. The equivalents of NaH and 2-bromo malonate used are in a range of 8-10 with addition of 2 equivalent

each 2 hours in 8-10 hours. Even if the reaction is left 24h stirring with an excess of NaH and bromo-malonate no formation of tris adducts is observed.

To see the HRMS monitoring of each reaction go to **Figure S58** (**Et**), **Figure S59** (***i*Pr**), **Figure S60** (***t*Bu**), **Figure S61** (**Bn**).

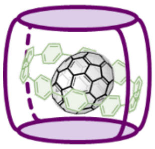

2-bromo malonates		12 o'clock	2 o'clock	5 o'clock
	**Et**	12%	72%	16%
	***i*Pr**	12%	45%	43%
	***t*Bu**	4%	73%	23%
	**Bn**	**0%**	**100%**	**0%**

Figure S23. Regioisomers ratio obtained for the Bingel reaction on C_{70}⊂[10]CPP⊂**6**·(BArF)$_8$

3.2.3 Isolation of α,α-*bis*-malonate- C_{70}⊂[10]CPP⊂6·(BArF)$_8$

The crude α,α-*bis*-malonate- C_{70}⊂[10]CPP⊂**6**·(BArF)$_8$ reaction mixture was washed with $CHCl_3$ affording a precipitate. The precipitate was dissolved in CH_3CN and C_{70} (4.0 equiv.) was added as a suspension in $CHCl_3$ (CH_3CN/$CHCl_3$ 1:1) and stirred at room temperature overnight. Then, the solution was dried with N_2 gas flow and the α,α-*bis*-C_{70}-adducts were obtained after $CHCl_3$ addition and filtration of the solid, C_{70}⊂[10]CPP⊂**6**·(BArF)$_8$ complex (that can be recovered adding CH_3CN). The three regioisomers were separated by preparative TLC (silica) in Toluene 100%. Each regioisomer has been characterized by HPLC and UV-Vis to confirm the identity, which was also cross-matched with an independently synthesized sample of the regioisomer. The yield of the reaction was calculated by HPLC using C_{60} as internal standard (taking into account the calibration factor calculated in section 3.4). The yield obtained is in the range of 29-43% depending on the bromomalonate used (diethyl= 29%; diisopropyl= 32%; ditertbutyl= 29%; dibenzyl= 43%).

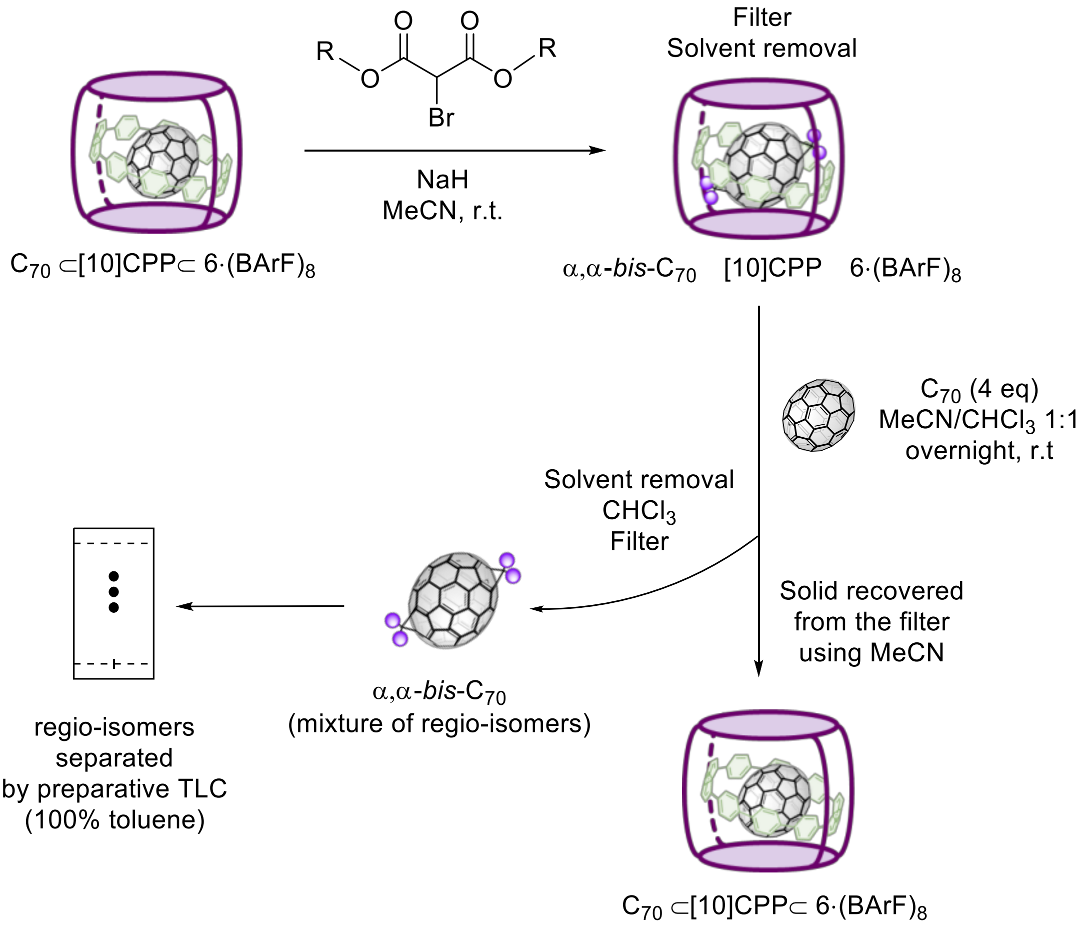

Figure S24. Detailed work-up flow for the isolation of α,α-*bis*-C_{70} -adducts obtained from Bingel reaction with C_{70}⊂[10]CPP⊂**6**·(BArF)$_8$ system.

3.2.4 Characterization α,α-*bis*-malonate-C_{70}⊂[10]CPP⊂6·(BArF)$_8$

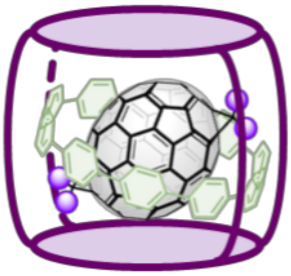

Figure S25. α,α-*bis*-malonate- C_{70}⊂[10]CPP⊂**6**·(BArF)$_8$

The formation of the α,α-*bis*-malonate-C_{70} -adducts inside the Matryoshka-like complex is confirmed by HRMS, taking into account that the three regioisomers obtained with one 2-bromo malonate (2 o'clock, 5 o'clock and 12 o'clock) present the same mass.

α,α-*bis*-diethylmalonate-C_{70}⊂[10]CPP⊂**6**·(BArF)$_{8}$ HRMS (m/z) (See **Figure S68**):

calculated 906.0920 and found 906.0748 (α,α-*bis*-diethylmalonate-C_{70}⊂[10]CPP⊂[**6**·(BArF)$_{0}$]$^{+8}$);

calculated 1158.9716 and found 1158.9536 (α,α-*bis*-diethylmalonate-C_{70}⊂[10]CPP⊂[**6**·(BArF)$_{1}$]$^{+7}$);

calculated 1495.9785 and found 1495.9804 (α,α-*bis*-diethylmalonate-C_{70}⊂[10]CPP⊂[**6**·(BArF)$_{2}$]$^{+6}$);

calculated 1967.7876 and found 1967.8097 (α,α-*bis*-diethylmalonate-C_{70}⊂[10]CPP⊂[**6**·(BArF)$_{3}$]$^{+5}$);

calculated 2675.5013 and found 2675.5735 (α,α-*bis*-diethylmalonate-C_{70}⊂[10]CPP⊂[**6**·(BArF)$_{4}$]$^{+4}$).

α,α-*bis*-diisopropylmalonate-C_{70}⊂[10]CPP⊂**6**·(BArF)$_{8}$ HRMS (m/z) (See **Figure S69**):

calculated 913.0998 and found 912.9011 (α,α-*bis*-diisopropylmalonate-C_{70}⊂[10]CPP⊂[**6**·(BArF)$_{0}$]$^{+8}$);

calculated 1166.9809 and found 1166.9033 (α,α-*bis*-diisopropylmalonate C_{70}⊂[10]CPP⊂[**6**·(BArF)$_{1}$]$^{+7}$);

calculated 1505.3222 and found 1505.2590 (α,α-*bis*-diisopropylmalonate-C_{70}⊂[10]CPP⊂[**6**·(BArF)$_{2}$]$^{+6}$);

calculated 1979.0001 and found 1978.9520 (α,α-*bis*-diisopropylmalonate-C_{70}⊂[10]CPP⊂[**6**·(BArF)$_{3}$]$^{+5}$);

calculated 2689.5170 and found 2689.5186 (α,α-*bis*-diisopropylmalonate-C_{70}⊂[10]CPP⊂[**6**·(BArF)$_{4}$]$^{+4}$).

α,α-*bis*-ditertbutylmalonate-C_{70}⊂[10]CPP⊂**6**·(BArF)$_{8}$ HRMS (m/z) (See **Figure S70**):

calculated 920.2327 and found 920.2214 (α,α-*bis*-ditertbutylmalonate-C_{70}⊂[10]CPP⊂[**6**·(BArF)$_{0}$]$^{+8}$);

calculated 1174.9898 and found 1174.9712 (α,α-*bis*-ditertbutylmalonate-C_{70}⊂[10]CPP⊂[**6**·(BArF)$_{1}$]$^{+7}$);

calculated 1514.6660 and found 1514.6218(α,α-*bis*-ditertbutylmalonate-C_{70}⊂[10]CPP⊂[**6**·(BArF)$_{2}$]$^{+6}$);

calculated 1990.2127 and found 1990.1471 (α,α-*bis*-ditertbutylmalonate-C_{70}⊂[10]CPP⊂[**6**·(BArF)$_{3}$]$^{+5}$);

calculated 2703.5327 and found 2703.4865 (α,α-*bis*-ditertbutylmalonate-C_{70}⊂[10]CPP⊂[**6**·(BArF)$_{4}$]$^{+4}$).

α,α-*bis*-dibenzylmalonate-C_{70}⊂[10]CPP⊂**6**·(BArF)$_{8}$ HRMS (m/z) (See **Figure S71**):

calculated 937.2249 and found 937.3617 (α,α-*bis*-dibenzylmalonate-C_{70}⊂[10]CPP⊂[**6**·(BArF)$_{0}$]$^{+8}$);

calculated 1194.4095 and found 1194.4265 (α,α-*bis*-dibenzylmalonate-C_{70}⊂[10]CPP⊂[**6**·(BArF)$_{1}$]$^{+7}$);

calculated 1537.3223 and found 1537.3469 (α,α-*bis*-dibenzylmalonate-C_{70}⊂[10]CPP⊂[**6**·(BArF)$_{2}$]$^{+6}$);

calculated 2017.4002 and found 2017.4153 (α,α-*bis*-dibenzylmalonate-C_{70}⊂[10]CPP⊂[**6**·(BArF)$_{3}$]$^{+5}$);

calculated 2737.5171 and found 2737.5183 (α,α-*bis*-dibenzylmalonate-C_{70}⊂[10]CPP⊂[**6**·(BArF)$_{4}$]$^{+4}$).

3.3 C_{70}⊂[10]CPP⊂7·(BArF)$_8$

3.3.1 Preparation of C_{70}⊂[10]CPP⊂7·(BArF)$_8$

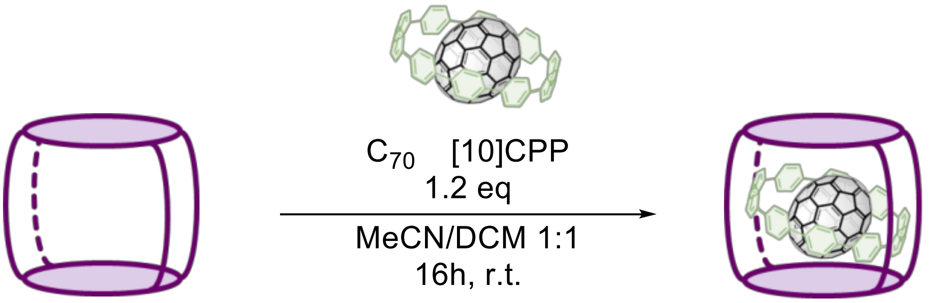

7·(BArF)$_8$ 1.0 eq

C_{70} [10]CPP 7·(BArF)$_8$ 1.0 eq

Figure S26. Synthesis of the Matryoshka-like complex C_{70}⊂[10]CPP⊂**7**·(BArF)$_8$.

Analogous synthesis to C_{70}⊂[10]CPP⊂**6**·(BArF)$_8$ has been carried out to obtain the Cu^{II}-based C_{70}⊂[10]CPP⊂**7**·(BArF)].

15 mg of **7**·(BArF)$_8$ nanocapsule (1 eq) are dissolved with the minimum amount of CH_3CN (1 mL). Then 1.2 eq of C_{70}⊂[10]CPP (2.35 mg) dissolved in DCM are added to the nanocapsule solution and stirred at room temperature for 16 h. After this time the solvent is removed under reduced pressure, MeCN is add to solubilize the product filtering to eliminate the excess of C_{70}⊂[10]CPP (that can be recovered adding $CHCl_3$).

3.3.2 Characterization of 7·(BArF)$_8$ and C_{70}⊂[10]CPP⊂[7·(BArF)$_8$]

7·(BArF)$_8$ HRMS (m/z) (See **Figure S72**):

calculated 623.5626 and found 623.5636 ([**7**·(BArF)$_0$]$^{+8}$);

calculated 835.9383 and found 835.9366 ([**7**·(BArF)$_1$]$^{+7}$);

calculated 1119.1059 and found 1119.1031 ([**7**·(BArF)$_2$]$^{+6}$);

calculated 1515.5405 and found 1515.5329 ([**7**·(BArF)$_3$]$^{+5}$);

calculated 2110.1925 and found 2110.1818 ([**7**·(BArF)$_4$]$^{+4}$).

C_{70}⊂[10]CPP⊂**7**·(BArF)$_8$ HRMS (m/z) (See **Figure S72**):

calculated 823.7273 and found 823.7140 (C_{70}⊂[10]CPP⊂[**7**·(BArF)$_0$]$^{+8}$);

calculated 1064.6979 and found 1064.6809 (C_{70}⊂[10]CPP⊂[**7**·(BArF)$_1$]$^{+7}$);

calculated 1385.9921 and found 1385.9680 (C_{70}⊂[10]CPP⊂[**7**·(BArF)$_2$]$^{+6}$);

calculated 1835.8040 and found 1835.7766 (C_{70}⊂[10]CPP⊂[**7**·(BArF)$_3$]$^{+5}$);

calculated 2510.7719 and found 2510.7252 (C_{70}⊂[10]CPP⊂[**7**·(BArF)$_4$]$^{+4}$).

3.4 Linear calibration for quantitative HPLC analysis

For the quantitative determination of yields by HPLC C_{60} was used as internal standard. Due to the different extinction coefficients of the bis-C_{70}-adducts and C_{60} two correction factor were determined by measuring peak area vs. concentration plots for C_{60} and for α,α-*bis*-dibenzylmalonate-C_{70} and α,α-*bis*-diethylmalonate-C_{70} where the concentration was determined by quantitative NMR.

The reasonable assumption is that the extinction coefficients of α,α-*bis*-diethylmalonate-C_{70} , α,α-*bis*-diisopropylmalonate-C_{70} and α,α-*bis*-ditetbutylmalonate-C_{70} are nearly identical, whereas the extinction coefficient of α,α-*bis*-dibenzylmalonate-C_{70} should be different thanks to the aromatic moieties, so it is calculated separately.

Is also considered that the extinction coefficients of the 2 o'clock, 12 o'clock and 5 o'clock regioisomers of the same bis-C_{70}-adduct are comparable.

In this perspective five solutions of the α,α-*bis*-dibenzylmalonate-C_{70} with concentration ranging from 13 to 214 μmol (calculated by ^{1}H NMR with internal standard) , five solutions of the α,α-*bis*-diethylmalonate-C_{70} with concentration ranging from 17 to 206 μmol (calculated by ^{1}H NMR with internal standard) and four solutions of C_{60} (commercial) with concentration ranging from 14 to 116 μmol were eluted over a Buckyprep-M column at a flow rate of 1.0 mL/min of toluene at 20 °C. The integrals (320 nm) at retention times of 10.1, 11.2 min and 12.9 min, respectively were plotted against the concentration. The correction factor "*f*" for quantitative HPLC was determined by comparison of the linear slope "*a*" of plots obtained from linear regression. This correction factor was used to calculate the HPLC yields for all the α,α-*bis*-C_{70}-adducts under the reasonable assumption, that their extinction coefficients are nearly identical.

$$f = \frac{a(bis - dibenzyl - adduct)}{a(C60)} = 0.81$$

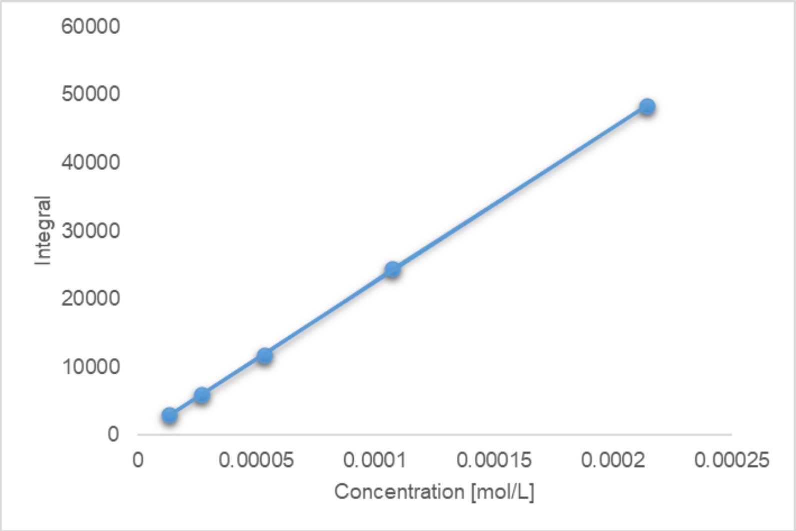

Equation	y = -97.191 + 226471695.782x
Slope	226471695.8
R-Square	0.9999

Figure S27. Peak area vs. concentration plot of α,α-*bis*-dibenzylmalonate-C_{70}.

$$f = \frac{a(bis - diethyl - adduct)}{a(C60)} = 1.04$$

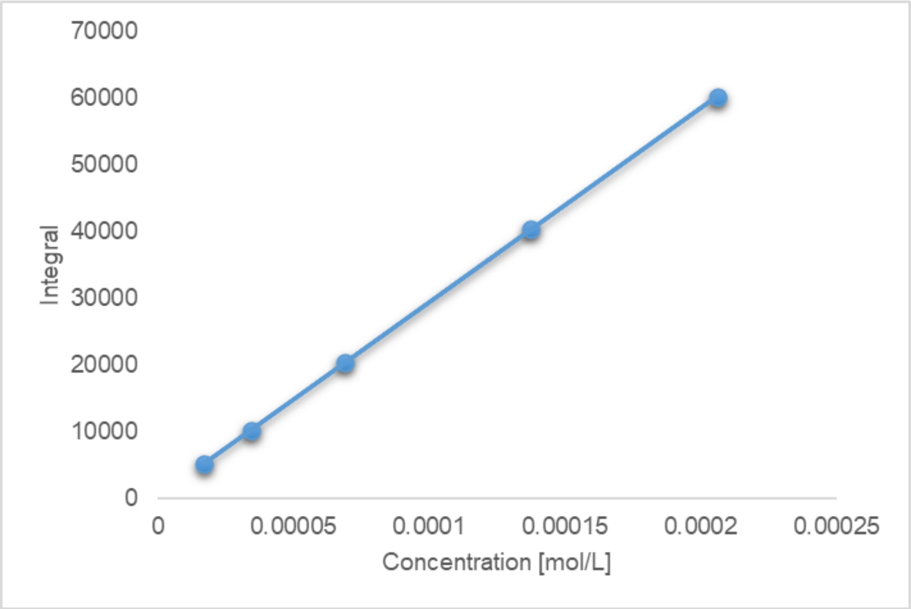

Equation	y = 137.582 + 291971653.016x
Slope	291971653
R-Square	1

Figure S28. Peak area vs. concentration plot of α,α-*bis*-diethylmalonate-C_{70}.

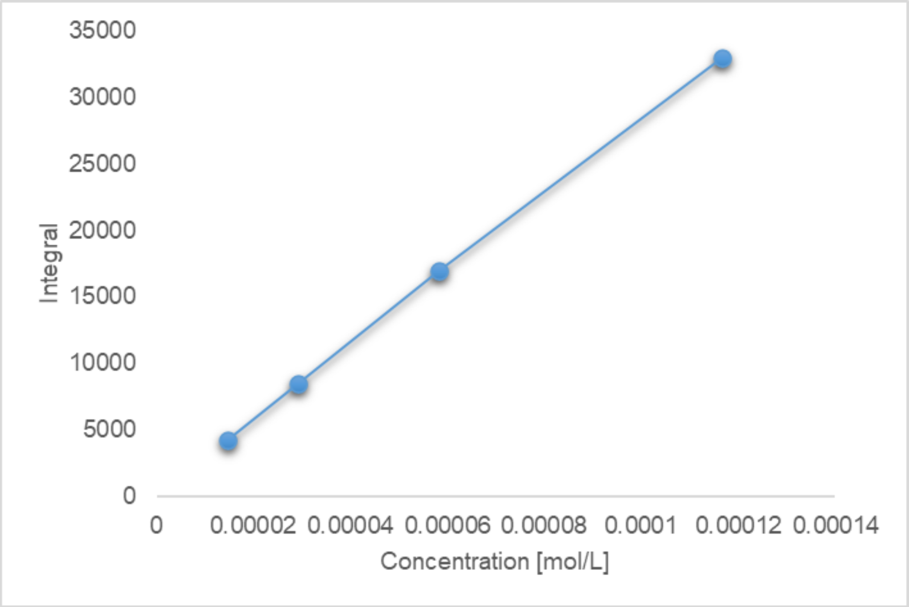

Equation	y = 341.486 + 280062844.965x
Slope	280062845
R-Square	0.9997

Figure S29. Peak area vs. concentration plot of C_{60}

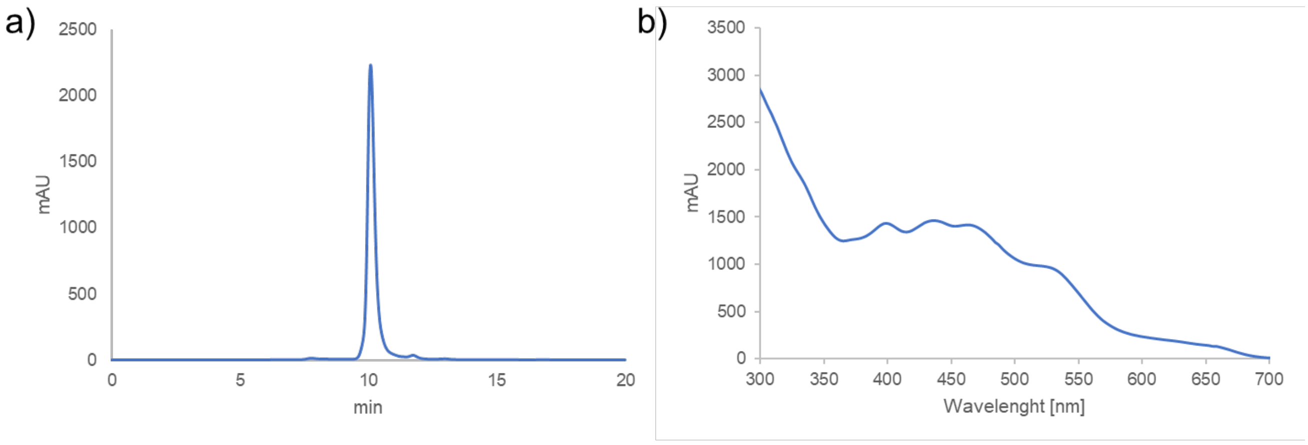

Figure S30 a) HPLC-DAD for α,α-*bis*-dibenzylmalonate-C_{70} b) UV-Vis Spectra showing the characteristic bands of 2 o'clock regioisomer.

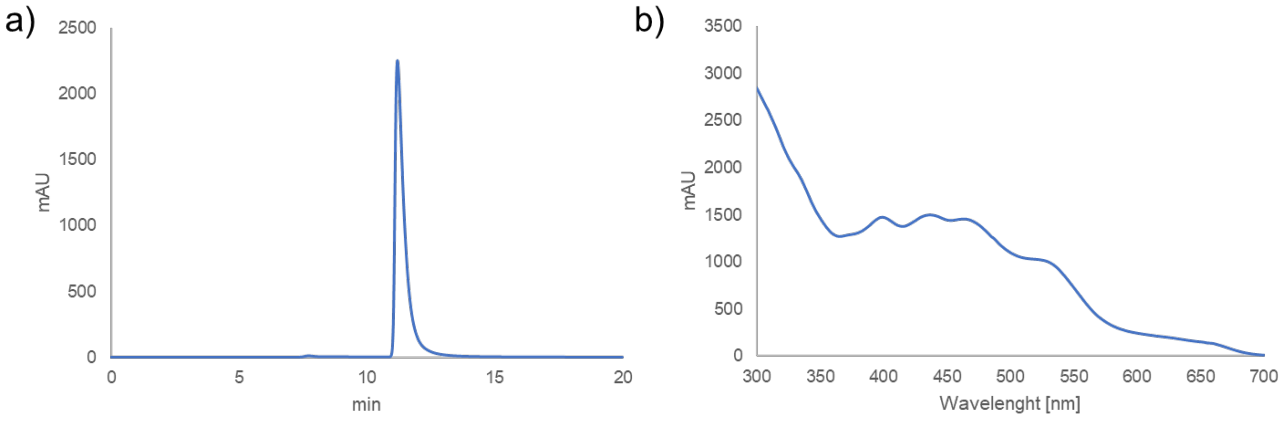

Figure S31 a) HPLC-DAD for α,α-*bis*-diethylmalonate-C_{70} b) UV-Vis Spectra showing the characteristic bands of 2 o'clock regioisomer.

4. Supplementary Figures

4.1 LDI Mass Spectra

The LDI mass spectra performed without matrix in positive mode indicate m/z values and isotope patterns consistent with α,α-*bis*-malonate-C_{70} .

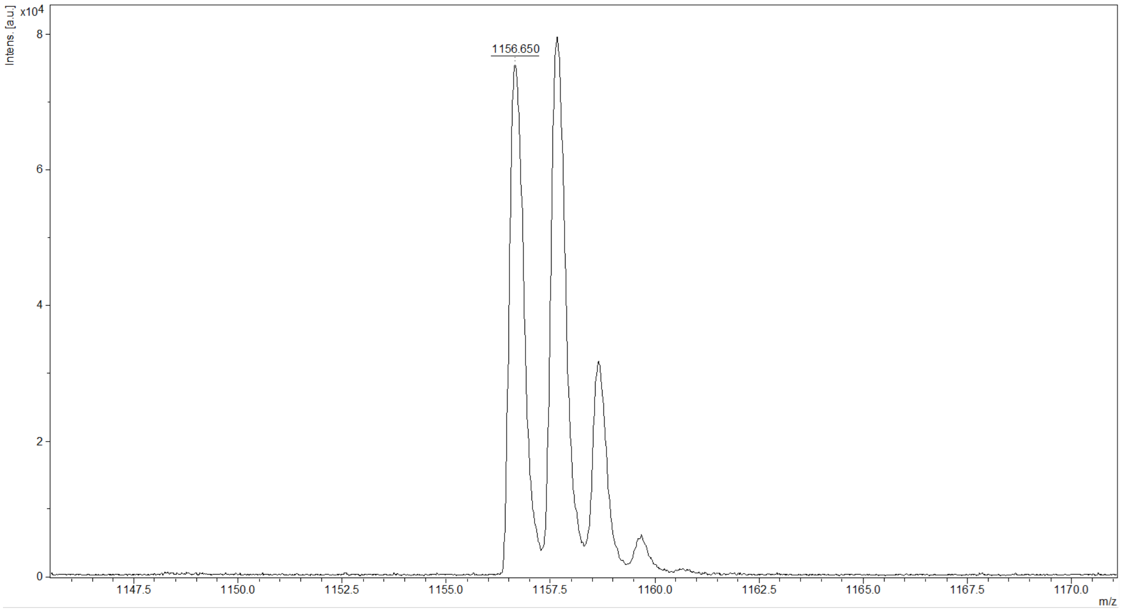

Figure S32. LDI mass spectra of α,α-*bis*-diethylmalonate-C_{70} (mixture of regioisomers). Calculated: 1156.1153 and found 1156.650.

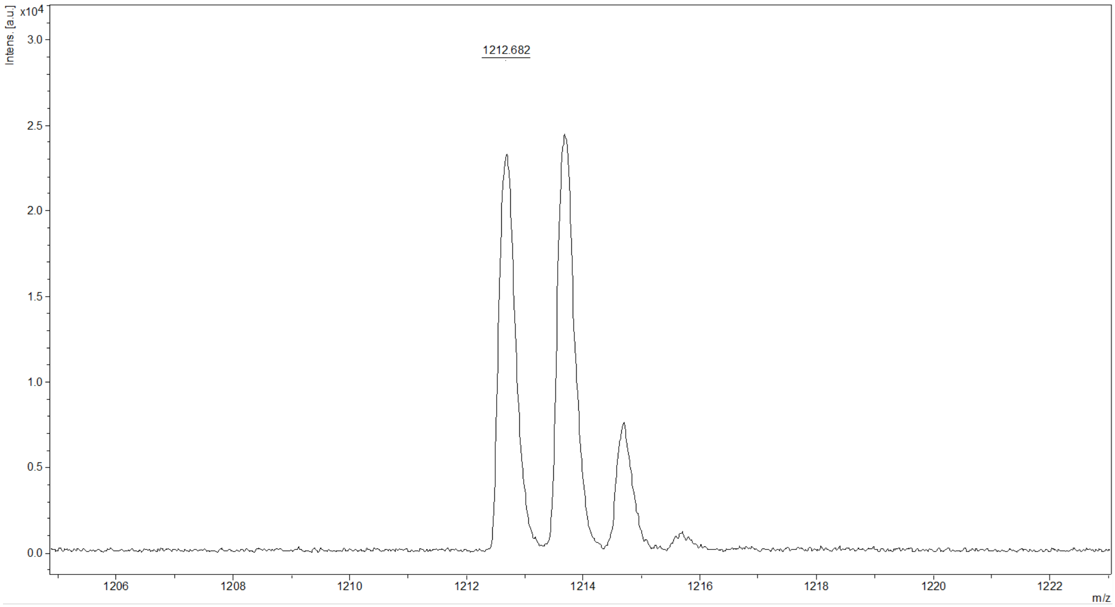

Figure S33. LDI mass spectra of α,α-*bis*-diisopropylmalonate-C_{70} (mixture of regioisomers). Calculated: 1212.1770 and found 1212.682

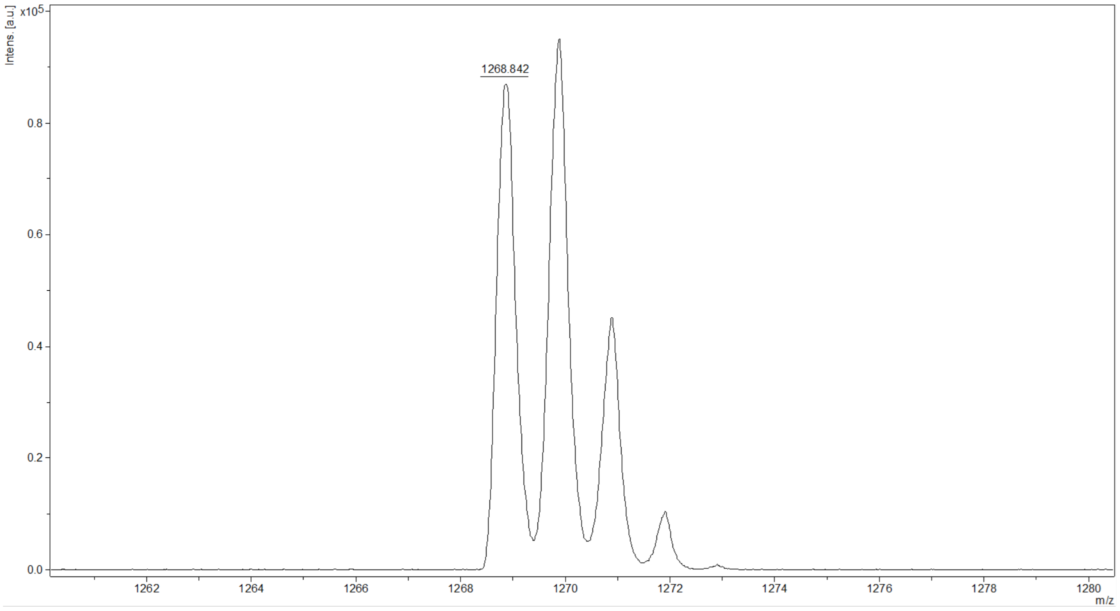

Figure S34. LDI mass spectra of α,α-*bis*-ditertbutylmalonate-C_{70} (mixture of regioisomers). Calculated: 1268.2405 and found 1268.842

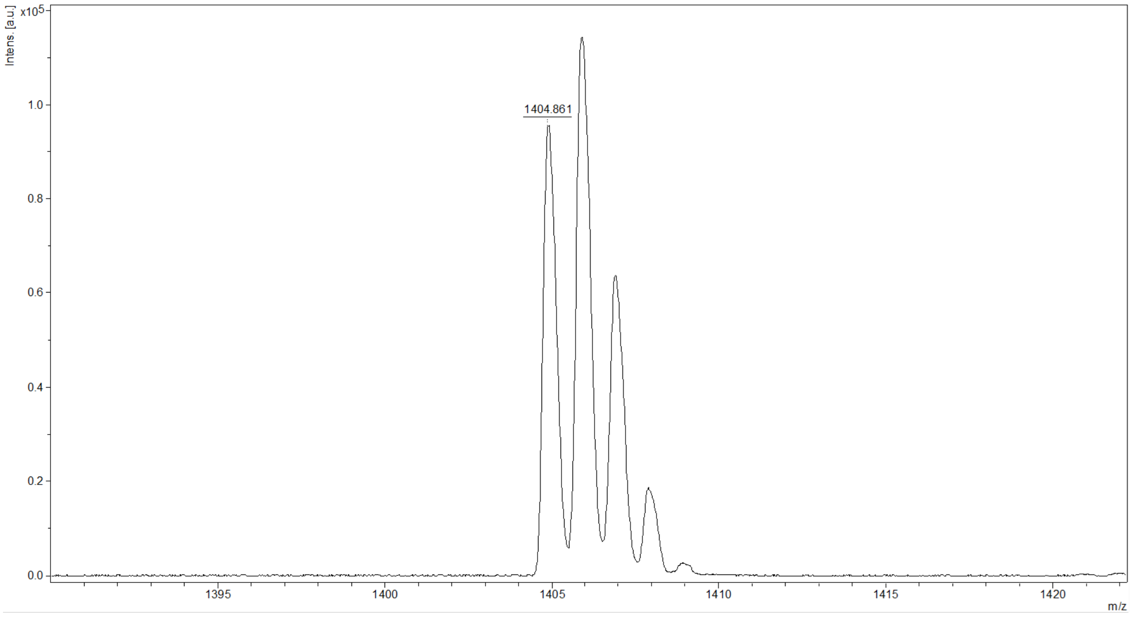

Figure S35. LDI mass spectra of α,α-*bis*-dibenzylmalonate-C_{70} (mixture of regioisomers). Calculated: 1404.1779 and found 1404.861

4.2 UV-Vis Spectra

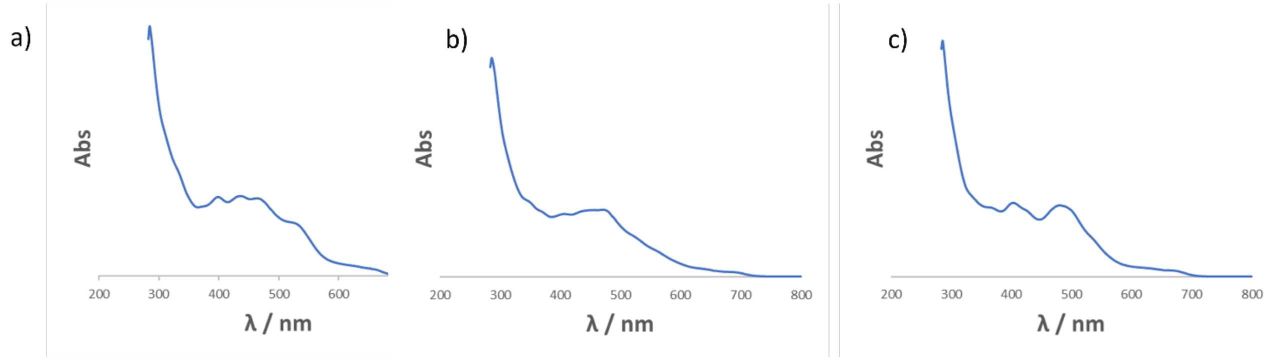

Figure S36. Spectra diagnostic of α,α-*bis*-malonate-C_{70} regioisomers, which are not dependent on the 2-bromo malonates used for the functionalization, but just on the bonds functionalized. a) 2 o'clock regioisomer; b) 5 o'clock regioisomer; c) 12 o'clock regioisomer.

4.3 NMR

<u>C_{70}⊂[10]CPP</u>

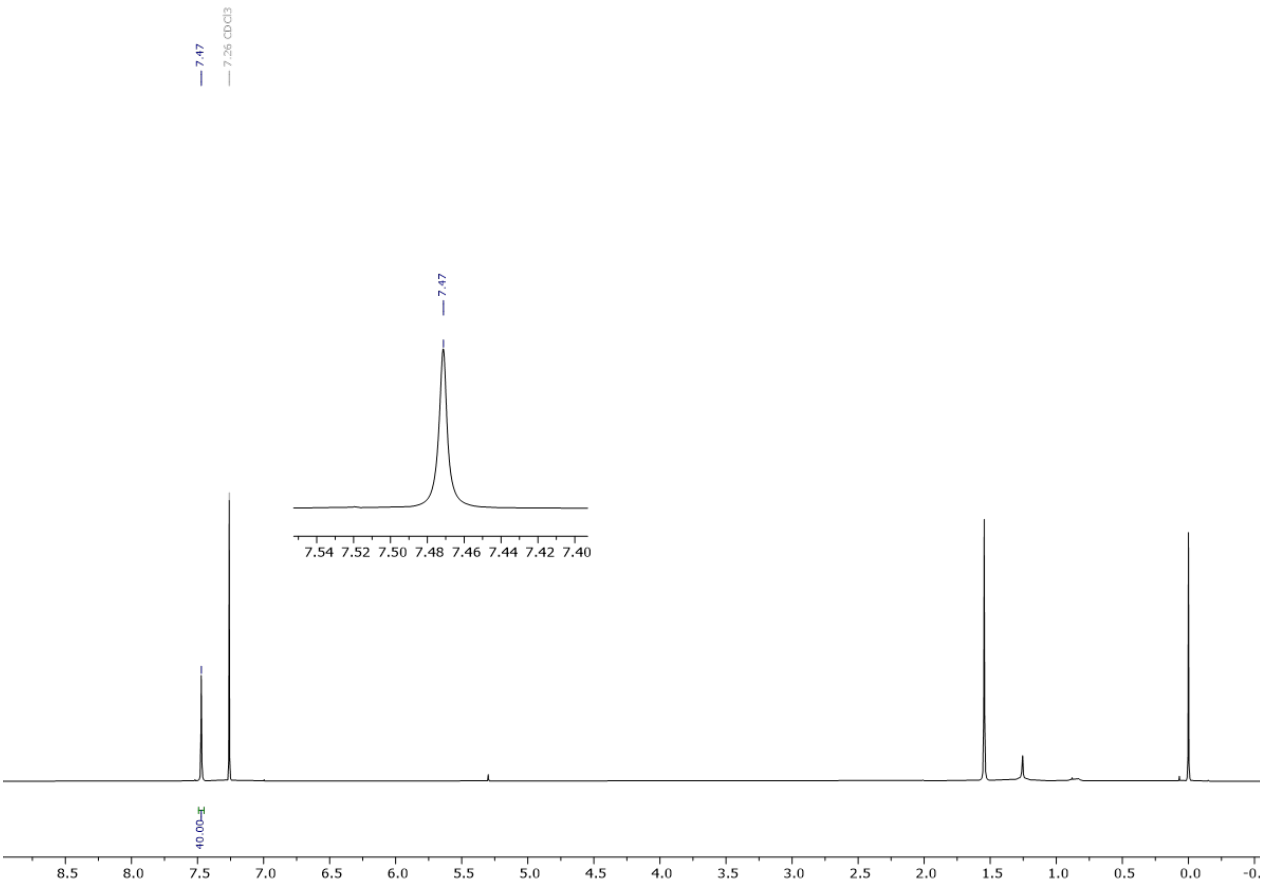

Figure S37. ^{1}H-NMR of C_{70}⊂[10]CPP (400 MHz, $CDCl_3$) δ p.p.m.: 7.47 (s, 40H, arom).

α,α-*bis*-diethylmalonate-C_{70} (2 o'clock regioisomer)

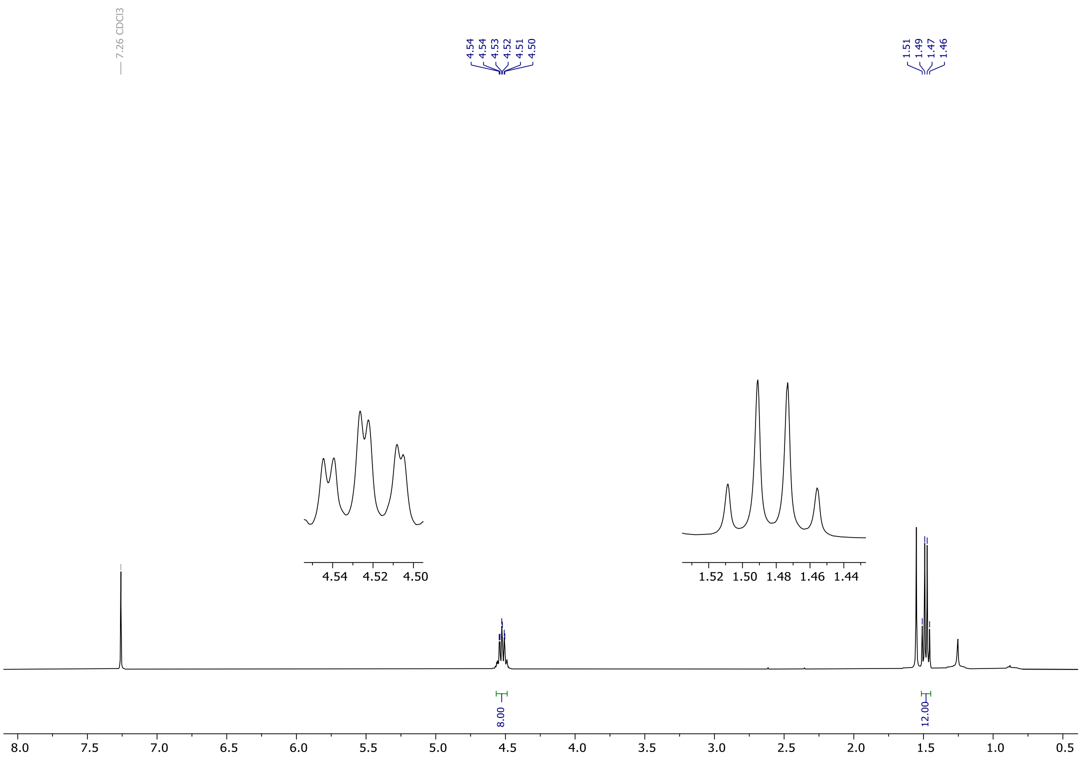

Figure S38. ^{1}H NMR of α,α-*bis*-diethylmalonate-C_{70} (2 o'clock regioisomer) (400 MHz, $CDCl_3$) δ p.p.m.: δ 4.52 (td, J = 7.1, 1.8 Hz, 8H), 1.48 (q, J = 7.1 Hz, 12H).

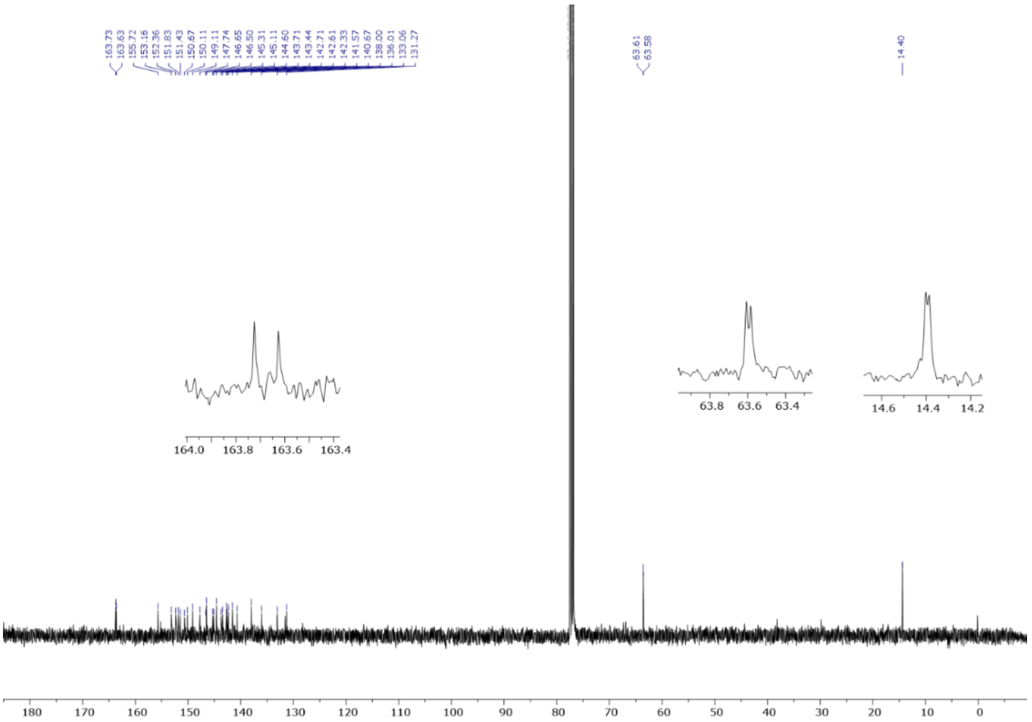

Figure S39. ^{13}C NMR of α,α-*bis*-diethylmalonate-C_{70} (2 o'clock regioisomer) (100 MHz, $CDCl_3$) δ p.p.m.: δ 163.73 (C=O), 163.63 (C=O), 155.72 - 131.27 (C_{70}), 63.61 (CH_2), 63.58 (CH_2), 14.40 (CH_3).

α,α-*bis*-diethylmalonate-C_{70} (5 o'clock regioisomer)

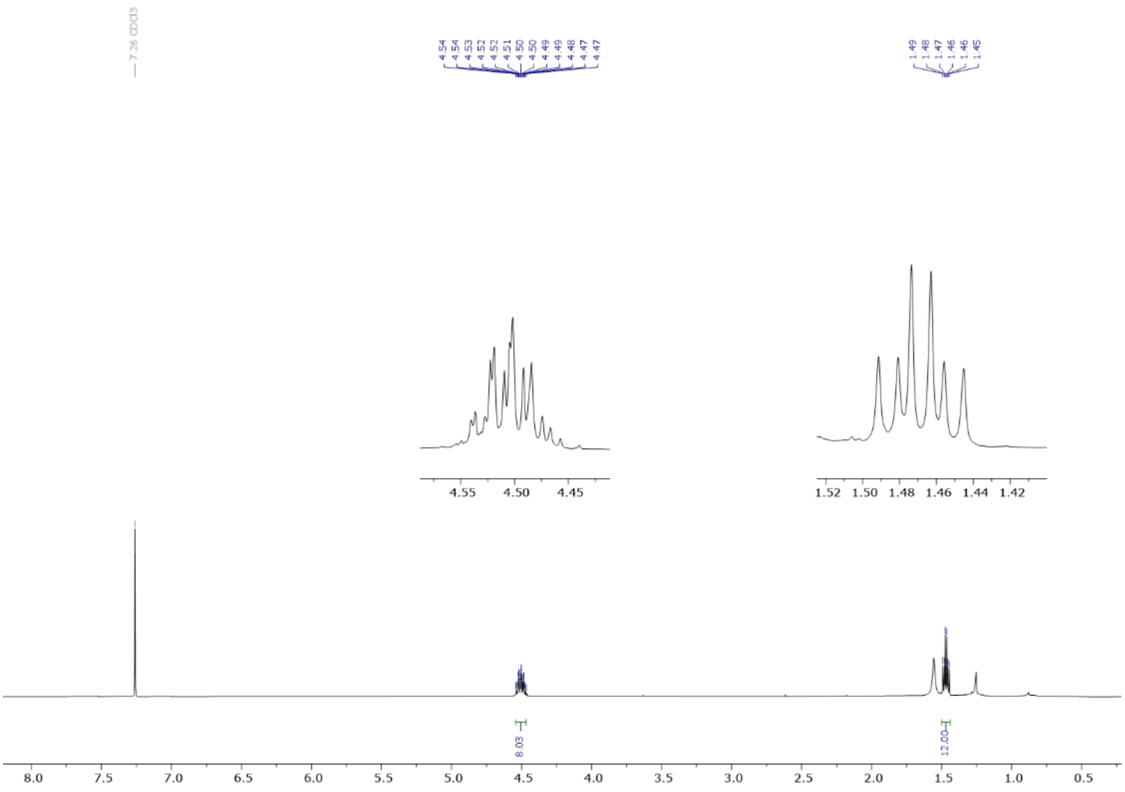

Figure S40. ^{1}H NMR of α,α-*bis*-diethylmalonate-C_{70} (5 o'clock regioisomer) (400 MHz, $CDCl_3$) δ p.p.m.: δ 4.54 – 4.47 (m, 8H), 1.47 (td, *J* = 7.1, 4.2 Hz, 12H).

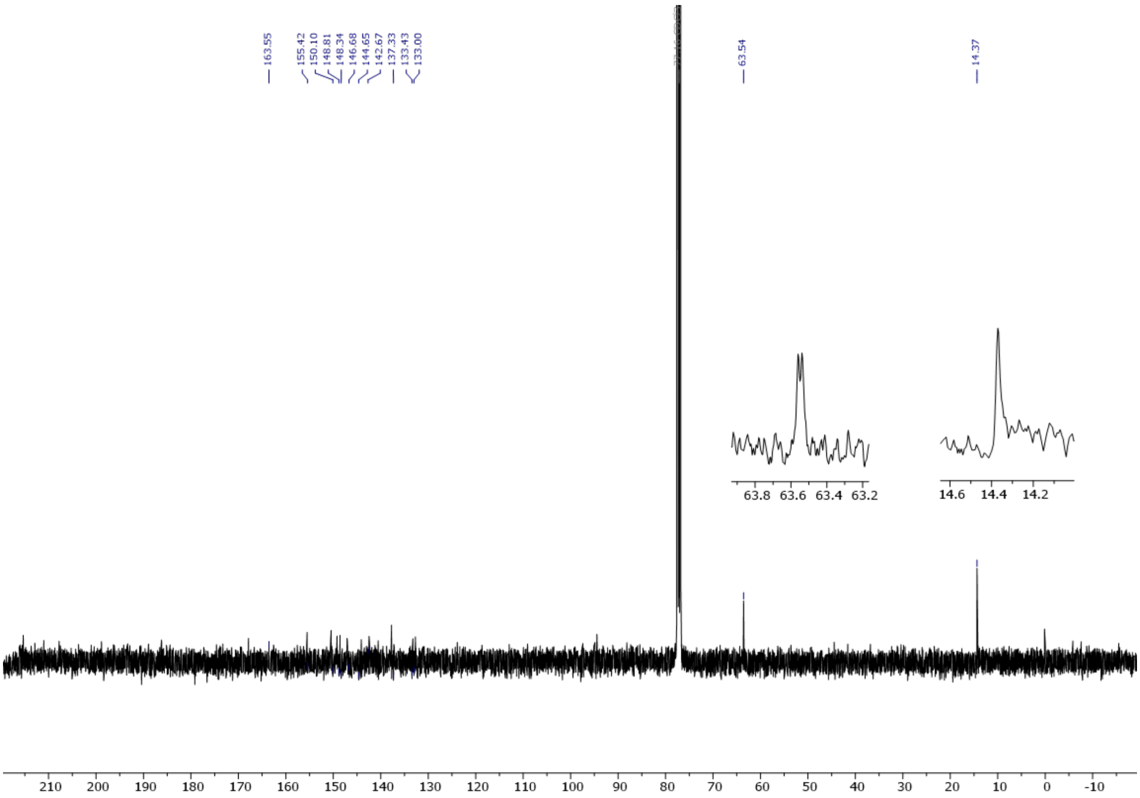

Figure S41. ^{13}C NMR of α,α-*bis*-diethylmalonate-C_{70} (5 o'clock regioisomer) (100 MHz, $CDCl_3$) δ p.p.m.: δ 163.55 (C=O); 155.42 – 133.00 (C_{70}); 63.54 (CH_2), 14.37 (CH_3).

α,α-*bis*-diethylmalonate-C_{70} (12 o'clock regioisomer)

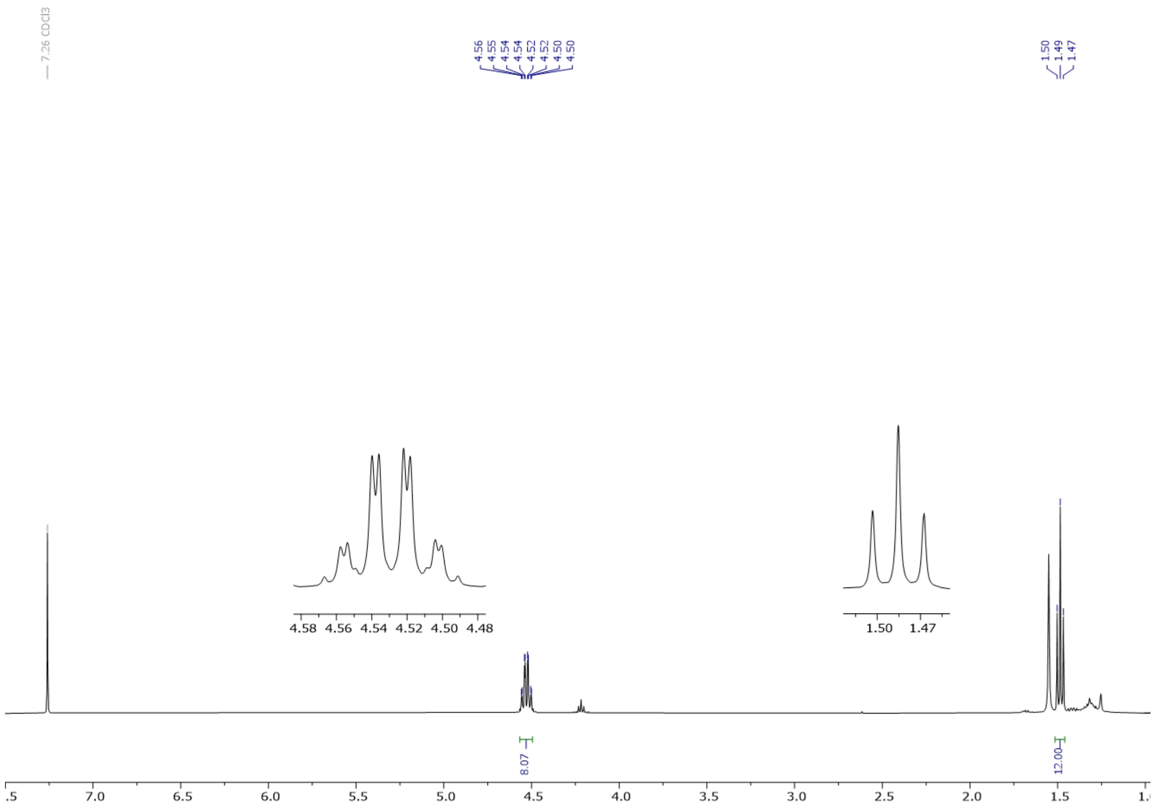

Figure S42. ^{1}H NMR of α,α-*bis*-diethylmalonate-C_{70} (12 o'clock regioisomer) (400 MHz, $CDCl_3$) δ p.p.m.: δ 4.53 (qd, J = 7.1, 1.6 Hz, 8H), 1.49 (t, J = 7.1 Hz, 12H).

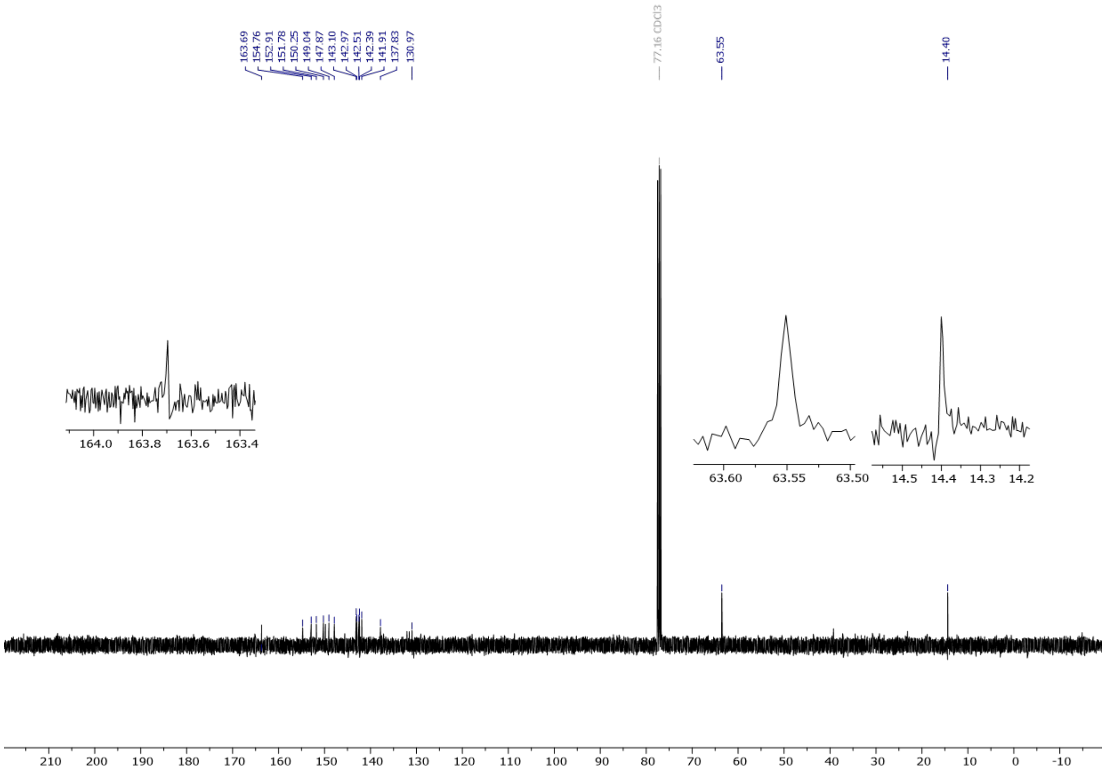

Figure S43. ^{13}C NMR of α,α-*bis*-diethylmalonate-C_{70} (12 o'clock regioisomer) (100 MHz, $CDCl_3$) δ p.p.m.: δ 163.7 (C=O) 154.76-130.97 (C_{70}), 63.55 (CH_2), 14.40 (CH_3).

α,α-*bis*-diisopropylmalonate-C_{70} (2 o'clock regioisomer)

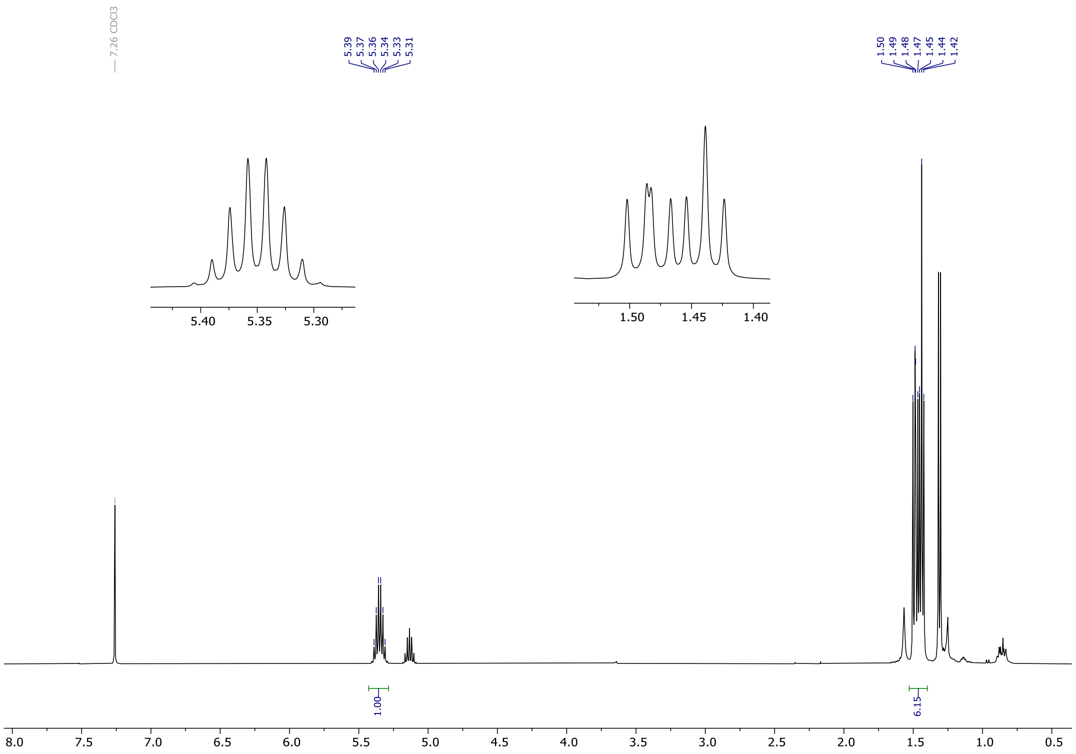

Figure S44. ^{1}H NMR of α,α-*bis*-diisopropylmalonate-C_{70} (2 o'clock regioisomer) (400 MHz, $CDCl_3$) δ p.p.m.: δ 5.35 (dq, *J* = 12.8, 6.4 Hz, 1H), 1.52 – 1.40 (m, 6H).

α,α-*bis*-diisopropylmalonate-C_{70} (5 o’clock regioisomer)

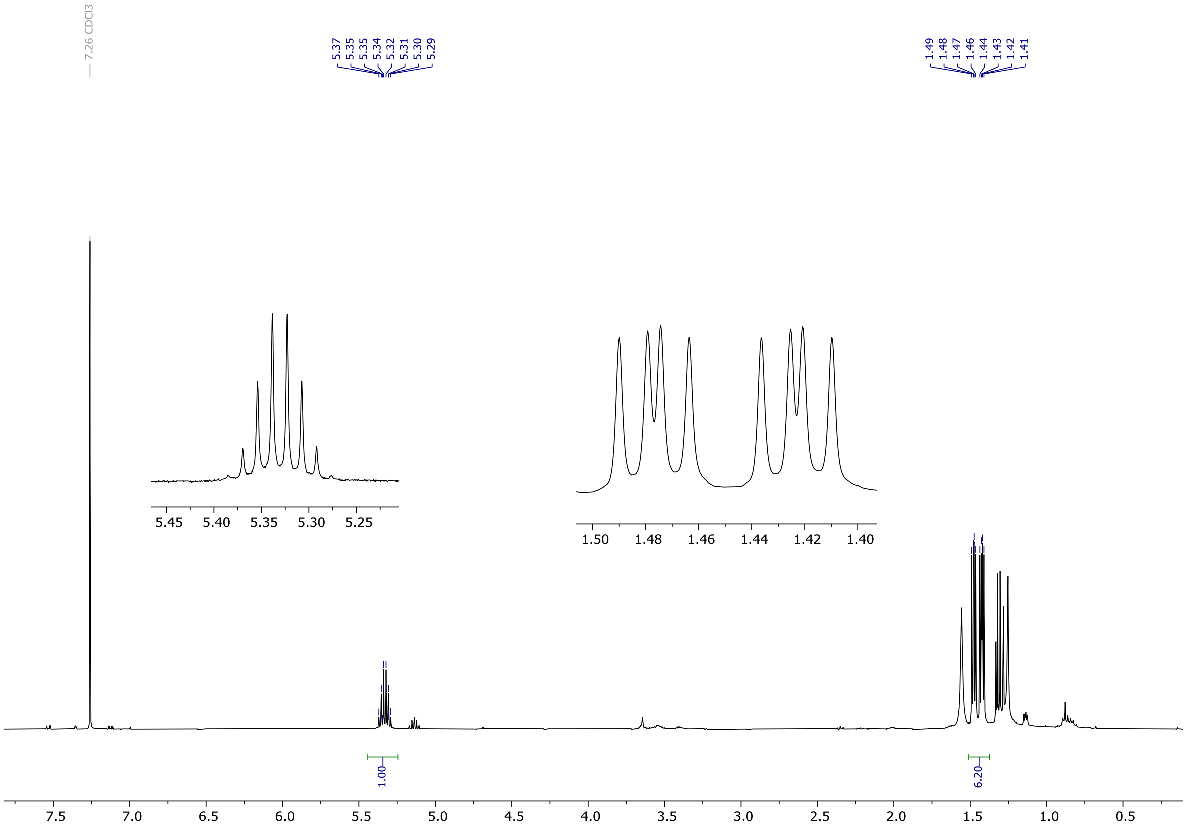

Figure S45. ^{1}H NMR of α,α-*bis*-diisopropylmalonate-C_{70} (5 o’clock regioisomer) (400 MHz, $CDCl_3$) δ p.p.m.: δ 5.34 (hept, *J* = 6.2 Hz, 1H), 1.45 (ddd, *J* = 21.5, 6.3, 4.3 Hz, 6H).

α,α-*bis*-diisopropylmalonate-C_{70} (12 o'clock regioisomer)

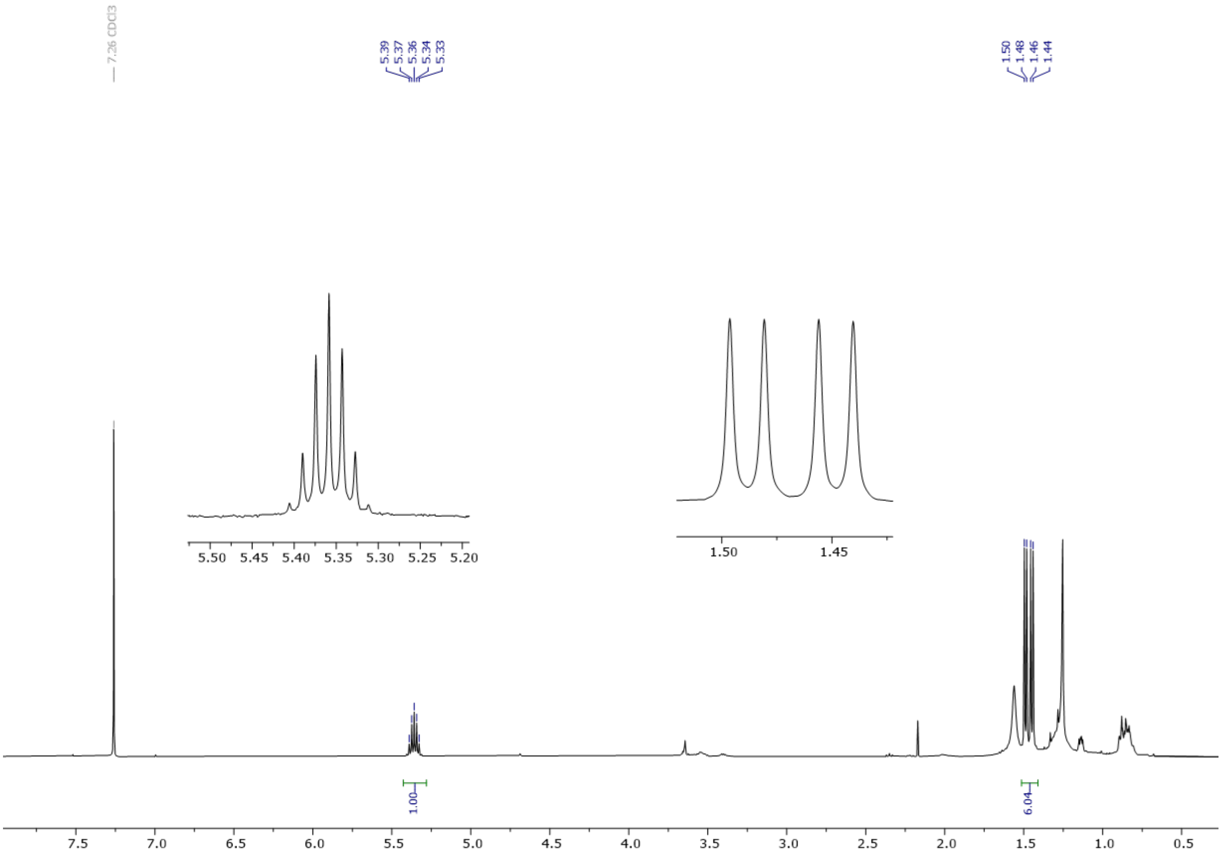

Figure S46. ^{1}H NMR of α,α-*bis*-diisopropylmalonate-C_{70} (12 o'clock regioisomer) (400 MHz, $CDCl_3$) δ p.p.m.: δ 5.36 (hept, *J* = 6.2 Hz, 1H), 1.47 (dd, *J* = 16.1, 6.3 Hz, 6H).

α,α-*bis*-ditertbutylmalonate-C_{70} (2 o'clock regioisomer)

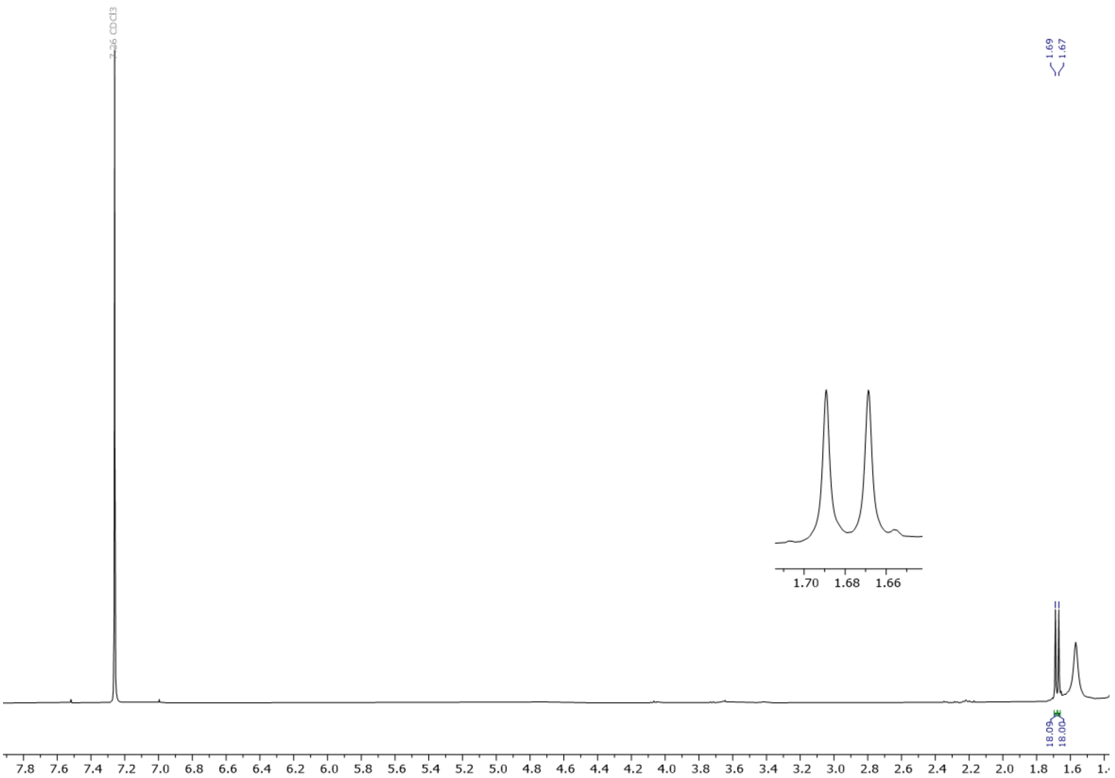

Figure S47. ^{1}H NMR of α,α-*bis*-ditertbutylmalonate-C_{70} (2 o'clock regioisomer) (400 MHz, $CDCl_3$) δ p.p.m.: δ 1.69 (s, 18H), 1.67 (s, 18H).

α,α-*bis*-ditertbutylmalonate-C_{70} (5 o'clock regioisomer)

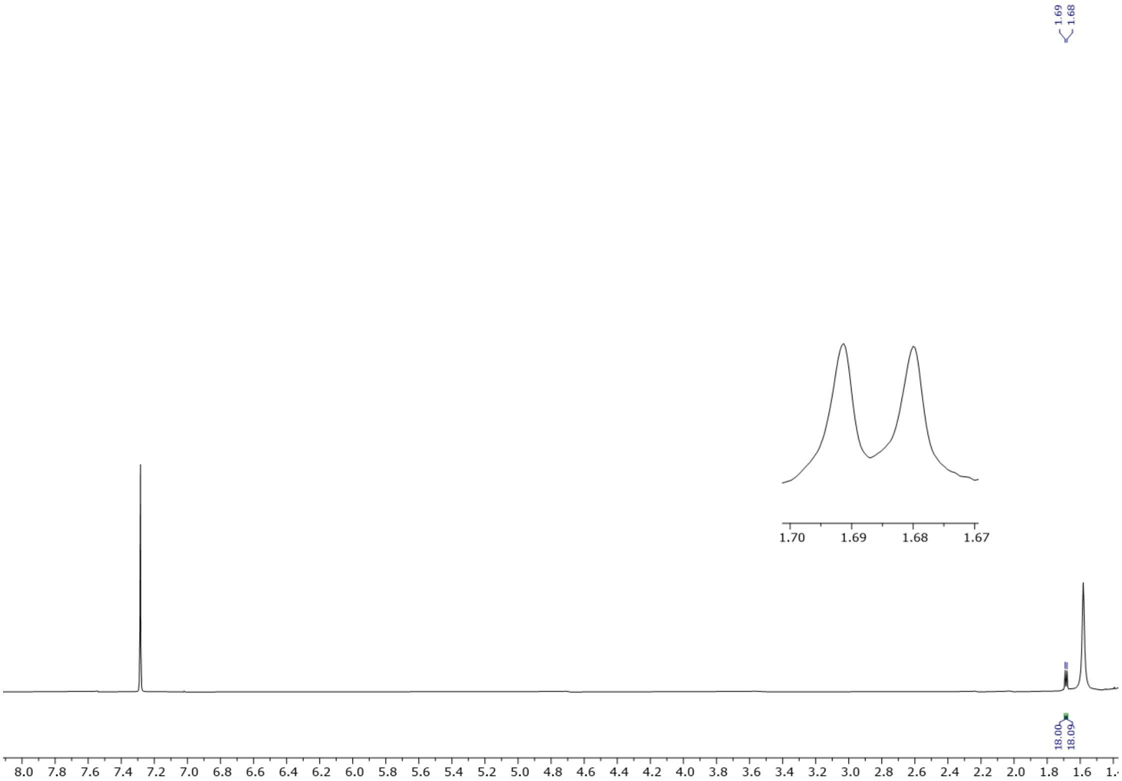

Figure S48. ^{1}H NMR of α,α-*bis*-ditertbutylmalonate-C_{70} (5 o'clock regioisomer) (400 MHz, $CDCl_3$) δ p.p.m.: δ 1.69 (s, 18H), 1.68 (s, 18H).

α,α-*bis*-ditertbutylmalonate-C_{70} (12 o'clock regioisomer)

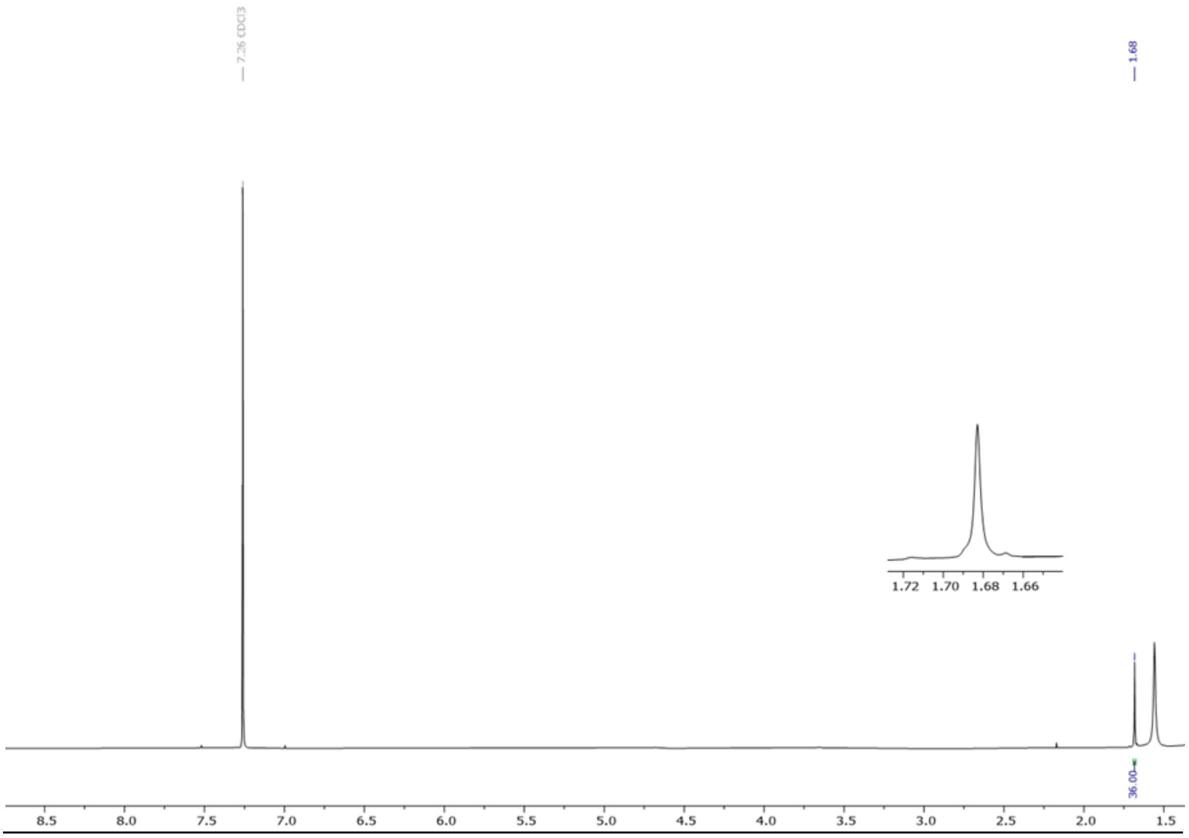

Figure S49. ^{1}H NMR of α,α-*bis*-ditertbutylmalonate-C_{70} (12 o'clock regioisomer) (400 MHz, $CDCl_3$) δ p.p.m.: δ 1.68 (s, 36H).

α,α-*bis*-dibenzylmalonate-C_{70} (2 o'clock regioisomer)

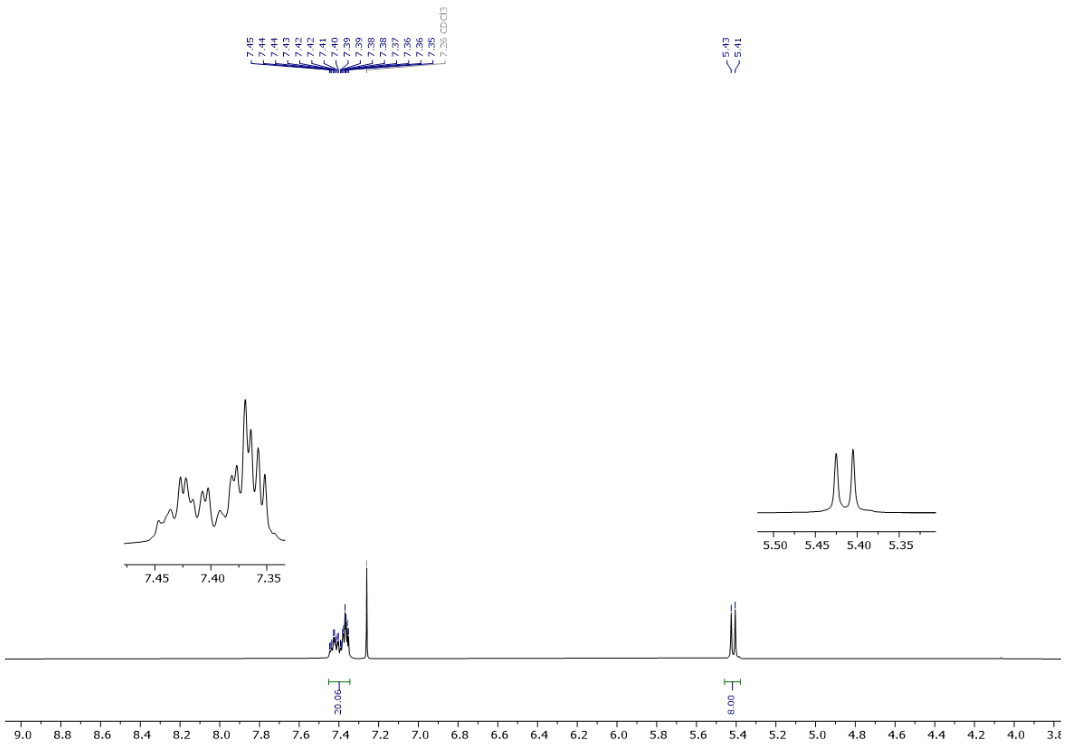

Figure S50. ^{1}H NMR of α,α-*bis*-dibenzylmalonate-C_{70} (2 o'clock regioisomer) (400 MHz, $CDCl_3$) δ p.p.m.: δ 7.45 – 7.34 (m, 20H), 5.42 (d, *J* = 8.1 Hz, 8H).

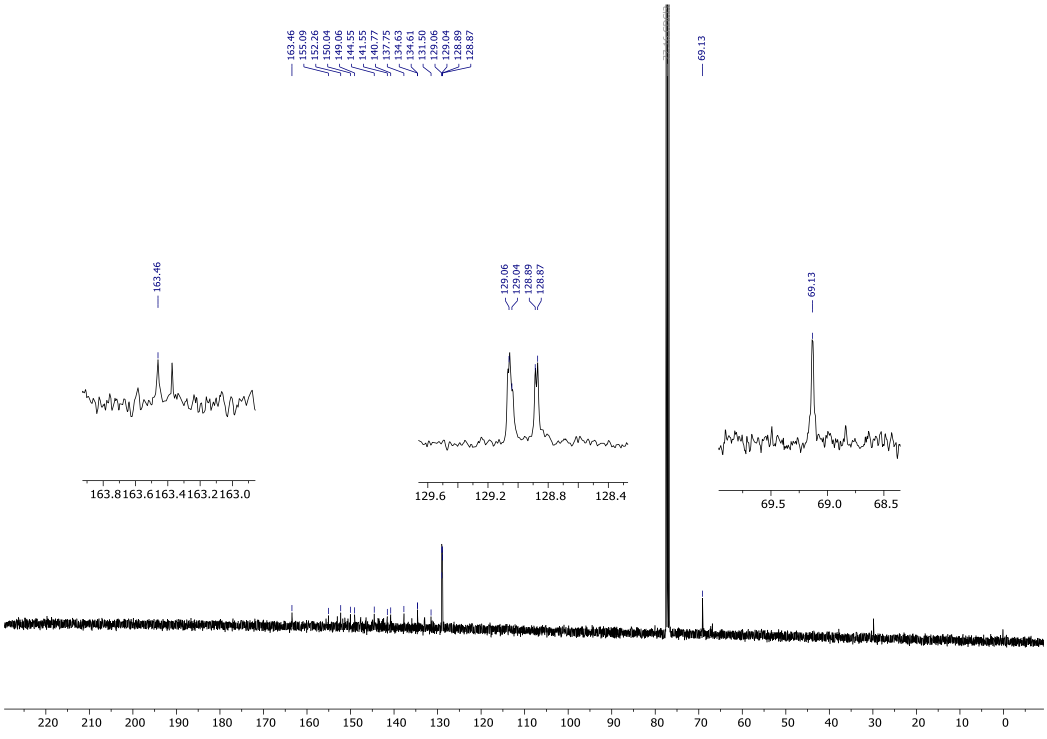

Figure S51. ^{13}C NMR of α,α-*bis*-dibenzylmalonate-C_{70} (2 o'clock regioisomer) (100 MHz, $CDCl_3$) δ p.p.m.: δ 163.46 (C=O), 163.37 (C=O), 155.62 – 131.51 (C_{70}), 129.06 – 128.87 (C-Ar), 69.13 (CH_2).

α,α-*bis*-dibenzylmalonate-C_{70} (5 o'clock regioisomer)

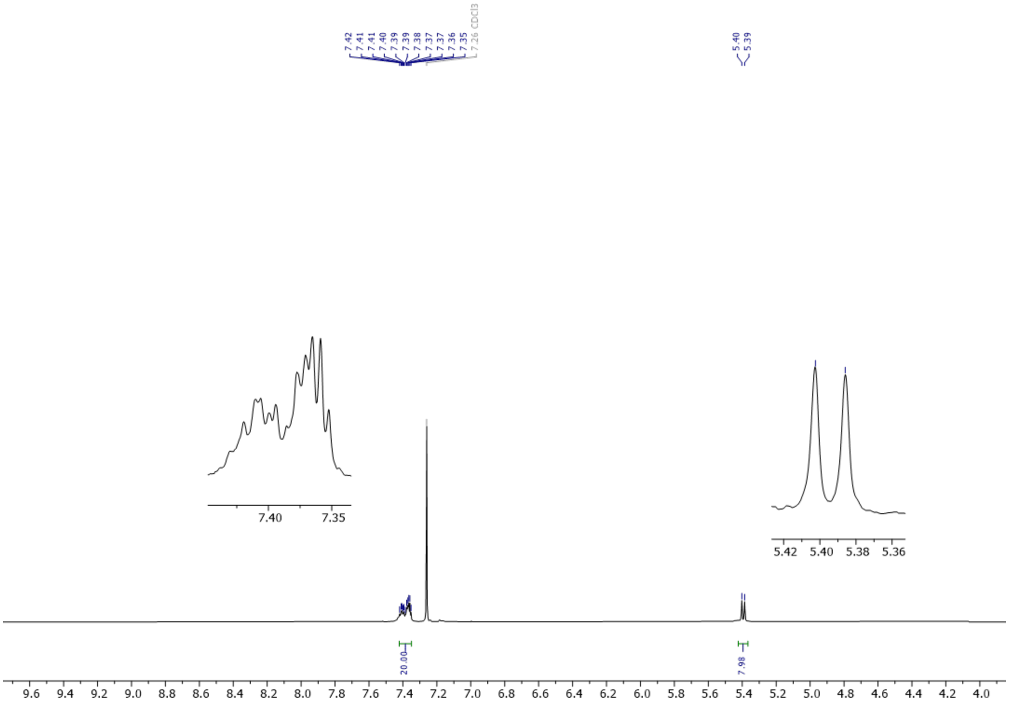

Figure S52. ^{1}H NMR of α,α-*bis*-dibenzylmalonate-C_{70} (5 o'clock regioisomer) (400 MHz, $CDCl_3$) δ p.p.m.: δ 7.42 – 7.35 (m, 20H), 5.39 (d, *J* = 6.6 Hz, 8H).

α,α-*bis*-dibenzylmalonate-C_{70} (12 o'clock regioisomer)

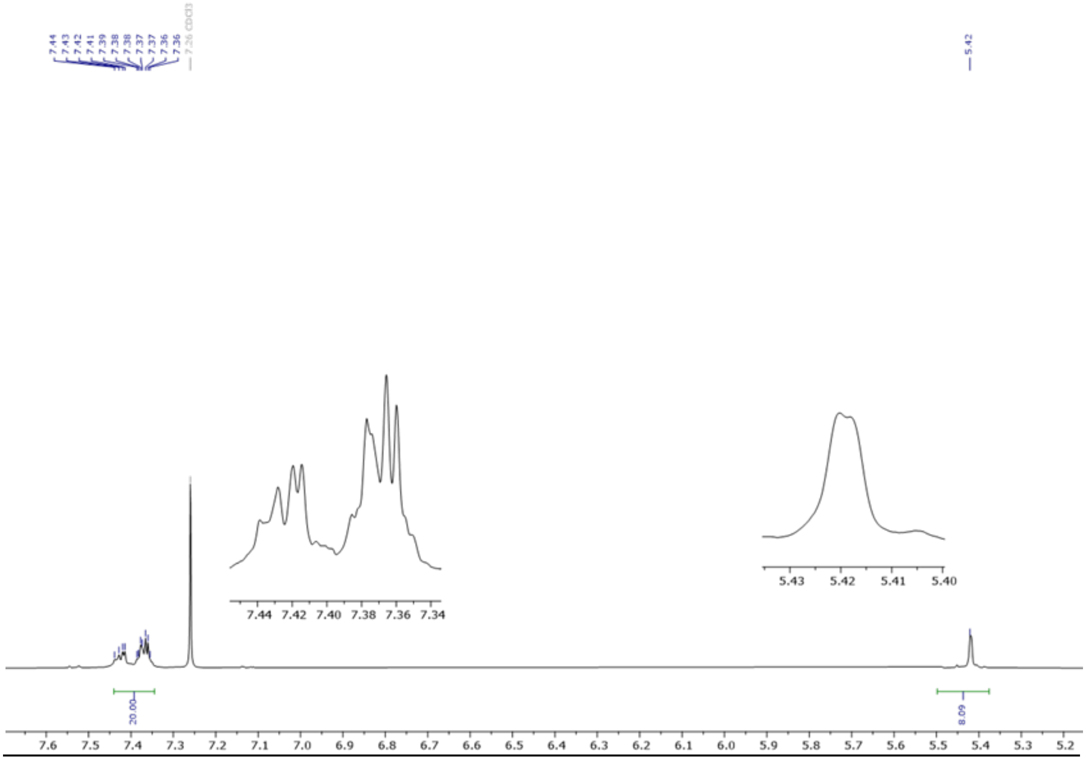

Figure S53. ^{1}H NMR of α,α-*bis*-dibenzylmalonate-C_{70} (12 o'clock regioisomer) (400 MHz, $CDCl_3$) δ p.p.m.: ^{1}H NMR (400 MHz, $CDCl_3$) δ 7.44 – 7.34 (m, 20H), 5.42 (s, broad 8H).

4.4 HRMS Monitoring

4.4.1 C_{70}⊂**4**·$(BArF)_8$

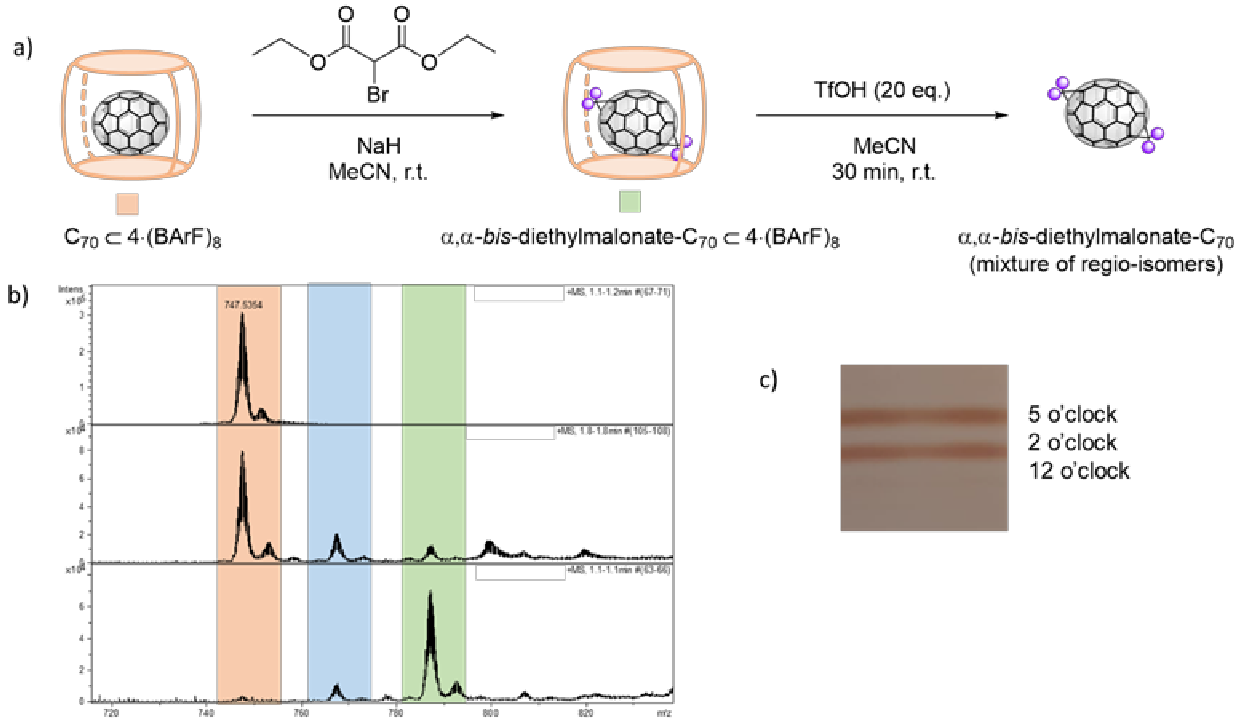

Figure S54. a) Exposure of_C_{70}⊂**4**·$(BArF)_8$ to Bingel cyclopropanation conditions with diethyl bromomalonate leads to the chemo-selective formation of the bis-adduct; the disassembly of the nanocapsule with TfOH liberates the product. b) HRMS monitoring of the bis-adduct formation upon addition of 3 equiv. bromomalonate (two sequential additions of 1.5 equiv.) in 3h. c) Preparative TLC (SiO_2, Toluene) is used to separate the three regioisomers.

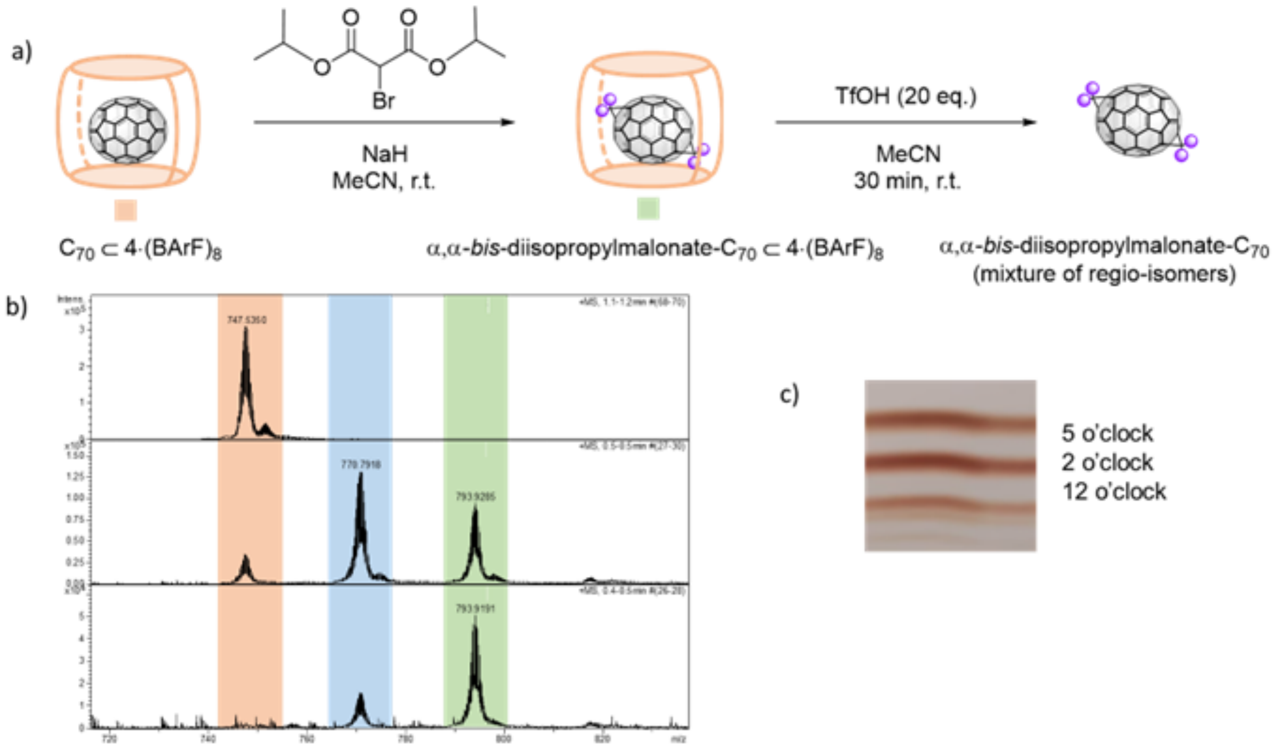

Figure S55. a) Exposure of_C_{70}⊂**4**·(BArF)$_8$ to Bingel cyclopropanation conditions with diisopropyl bromomalonate leads to the chemo-selective formation of the bis-adduct; the disassembly of the nanocapsule with TfOH liberates the product. b) HRMS monitoring of the bis-adduct formation upon addition of 4 equiv. bromomalonate (two sequential additions of 2.0 equiv.) in 4h. c) Preparative TLC (SiO_2, Toluene) is used to separate the three regioisomers.

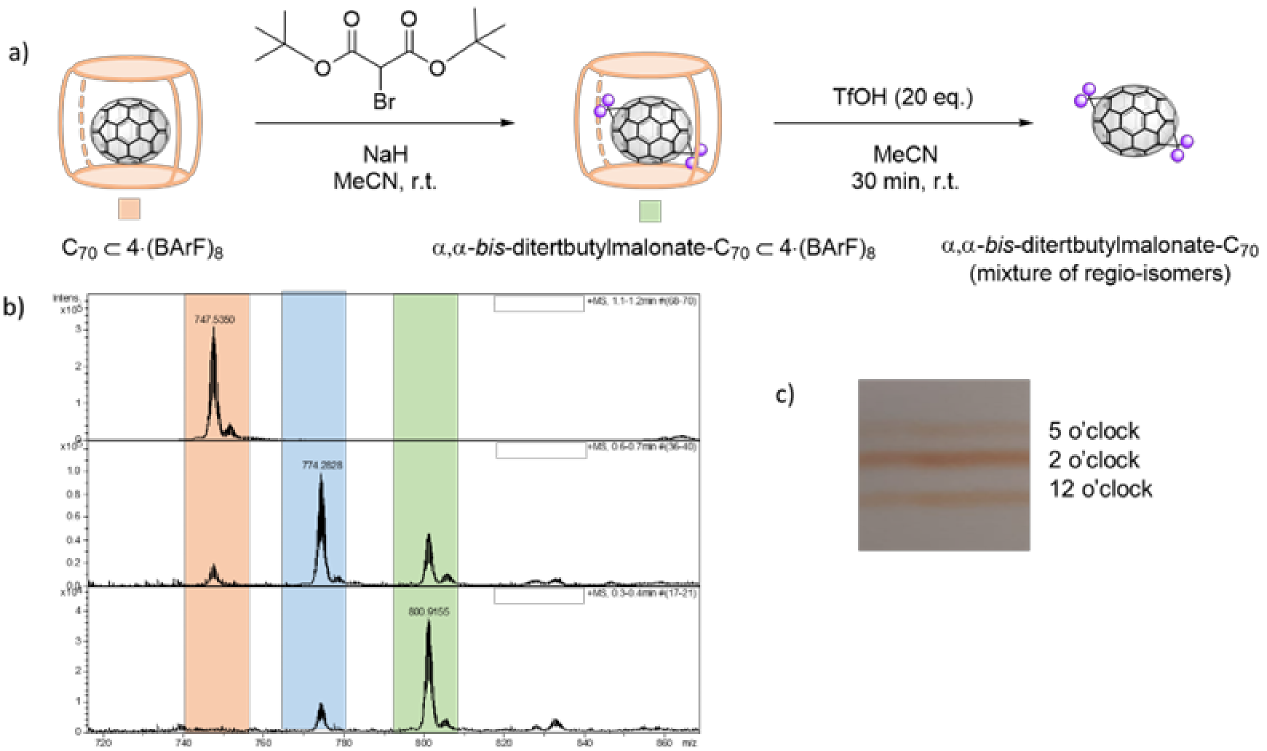

Figure S56. a) Exposure of_C_{70}⊂**4**·$(BArF)_8$ to Bingel cyclopropanation conditions with ditertbutyl bromomalonate leads to the chemo-selective formation of the bis-adduct; the disassembly of the nanocapsule with TfOH liberates the product. b) HRMS monitoring of the bis-adduct formation upon addition of 4 equiv. bromomalonate (two sequential additions of 2.0 equiv.) in 4h. c) Preparative TLC (SiO_2, Toluene) is used to separate the three regioisomers.

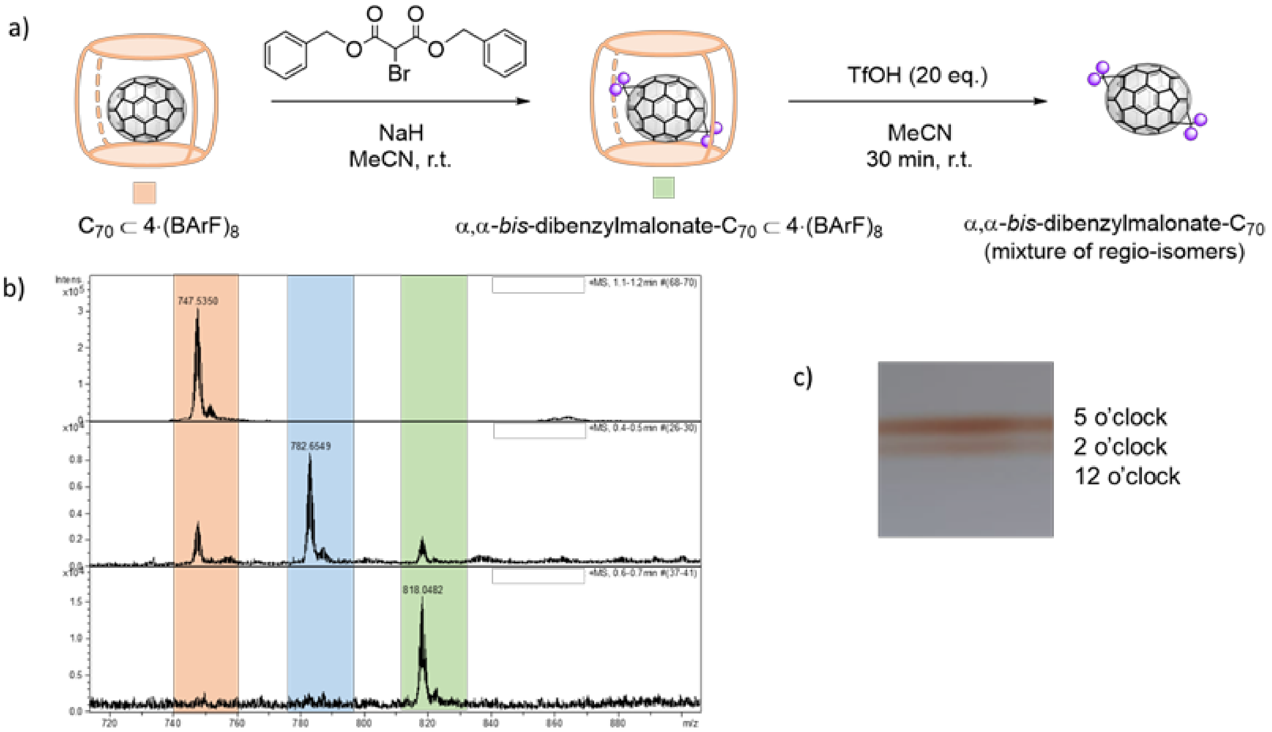

Figure S57. a) Exposure of_C_{70}⊂**4**·$(BArF)_8$ to Bingel cyclopropanation conditions with dibenzyl bromomalonate leads to the chemo-selective formation of the bis-adduct; the disassembly of the nanocapsule with TfOH liberates the product. b) HRMS monitoring of the bis-adduct formation upon addition of 4 equiv. bromomalonate (two sequential additions of 2.0 equiv.) in 4h. c) Preparative TLC (SiO_2, Toluene) is used to separate the three regioisomers.

4.4.2 C_{70}⊂[10]CPP⊂6·(BArF)$_8$

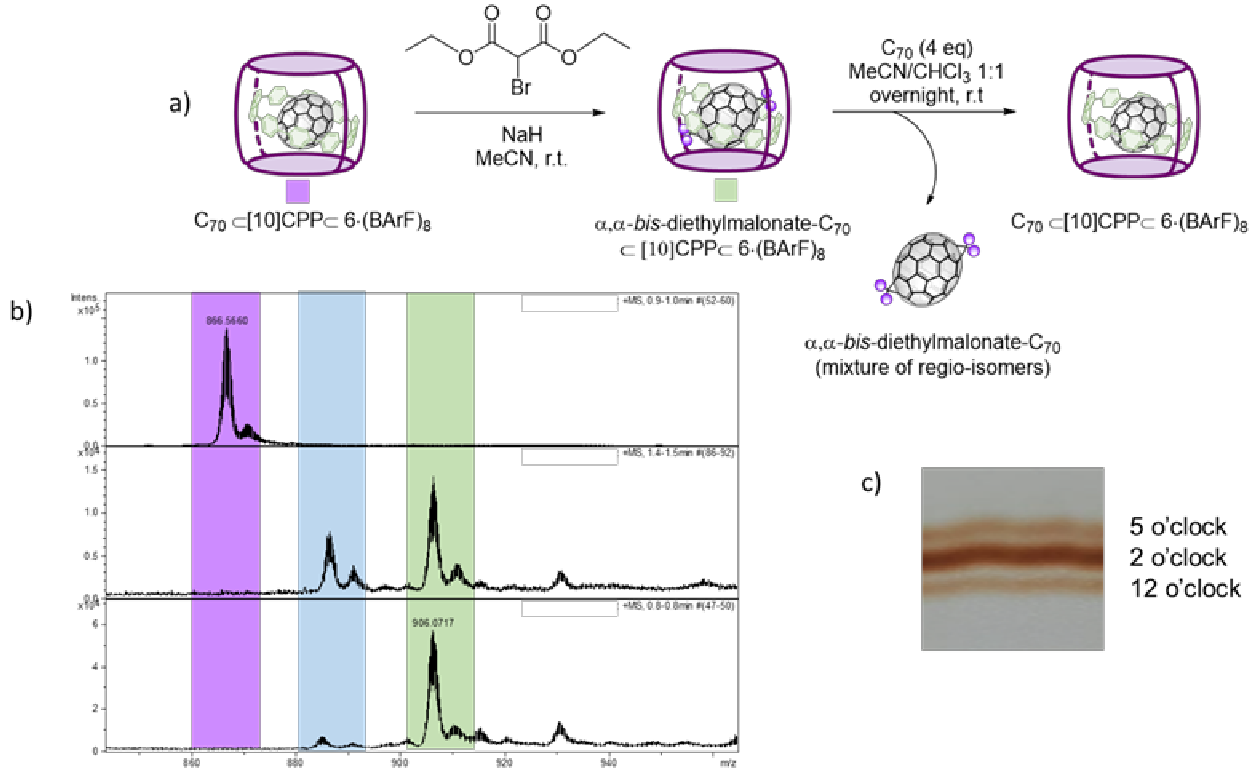

Figure S58. a) Exposure of_C_{70}⊂[10]CPP⊂**6**·(BArF)$_8$ to Bingel cyclopropanation conditions with diethyl bromomalonate leads to the chemo-selective formation of the bis-adduct; exchange with pristine C_{70} liberates the product. b) HRMS monitoring of the bis-adduct formation upon addition of 8 equiv. bromomalonate (four sequential additions of 2.0 equiv.) in 8h. c) Preparative TLC (SiO_2, Toluene) is used to separate the three regioisomers.

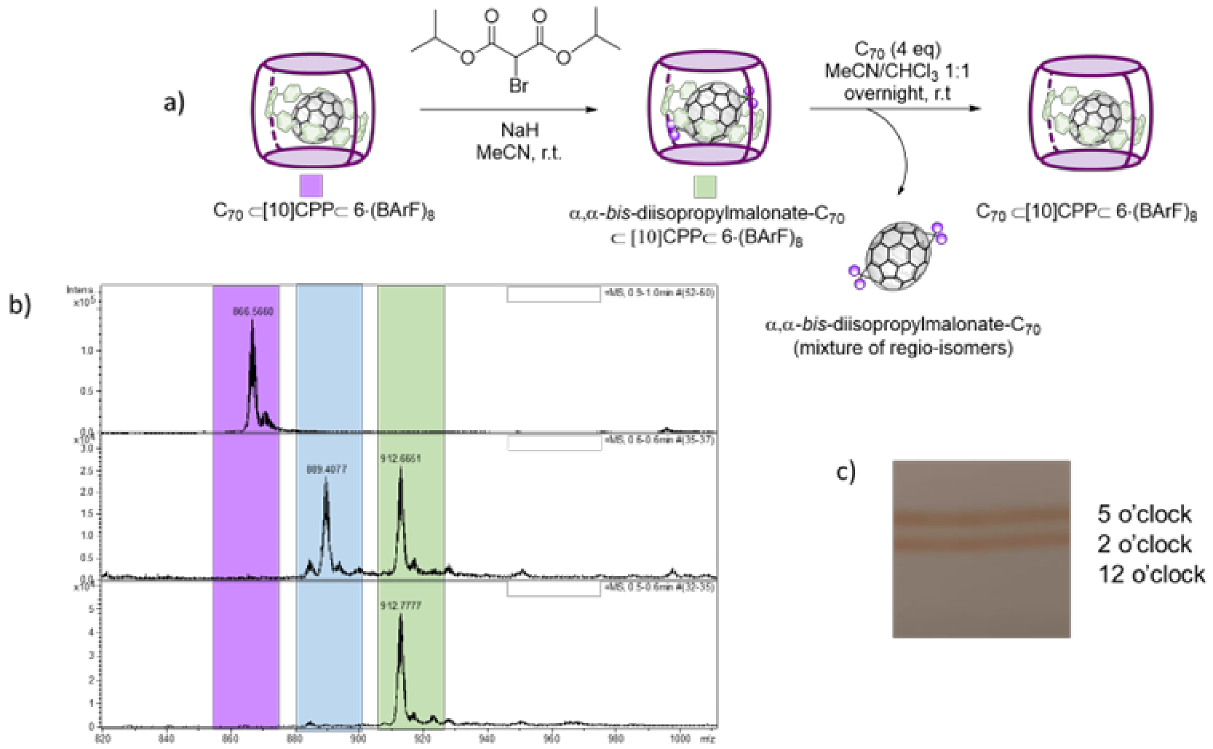

Figure S59. a) Exposure of_C_{70}⊂[10]CPP⊂**6**·(BArF)$_8$ to Bingel cyclopropanation conditions with diisopropyl bromomalonate leads to the chemo-selective formation of the bis-adduct; exchange with pristine C_{70} liberates the product. b) HRMS monitoring of the bis-adduct formation upon addition of 8 equiv. bromomalonate (four sequential additions of 2.0 equiv.) in 8h. c) Preparative TLC (SiO_2, Toluene) is used to separate the three regioisomers.

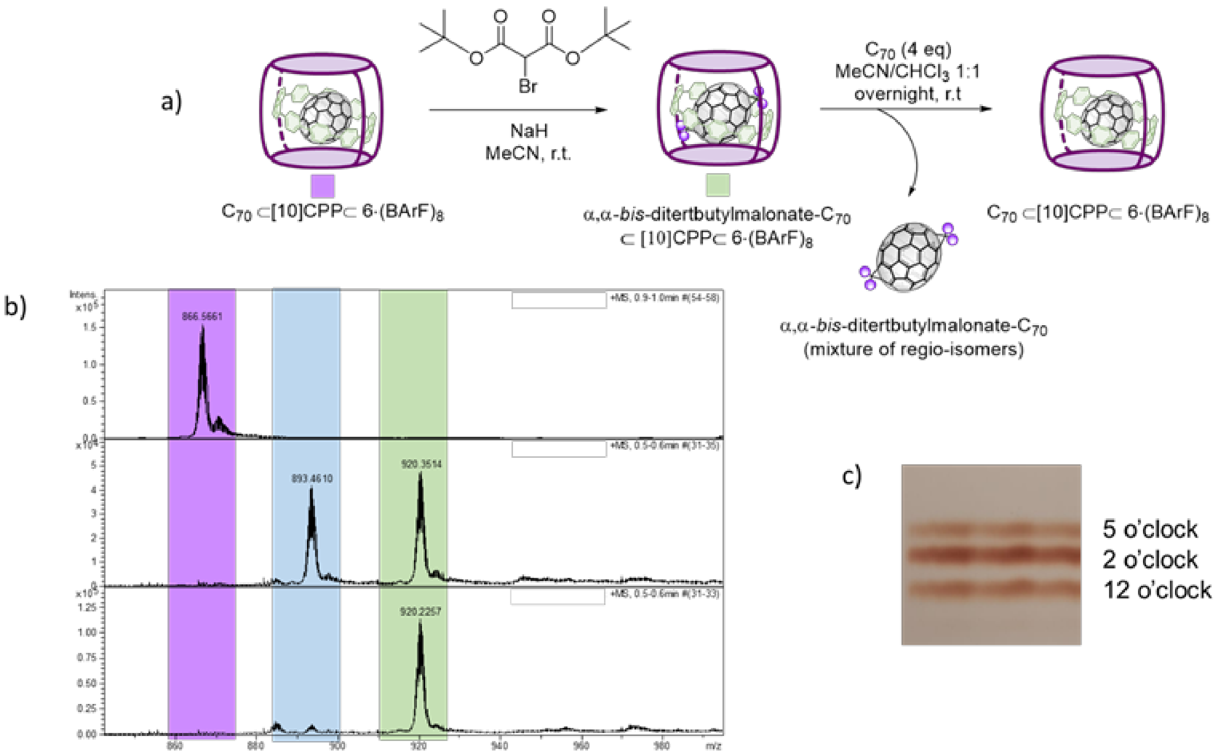

Figure S60. Exposure of_C_{70}⊂[10]CPP⊂**6**·$(BArF)_8$ to Bingel cyclopropanation conditions with ditertbutyl bromomalonate leads to the chemo-selective formation of the bis-adduct; exchange with pristine C_{70} liberates the product. b) HRMS monitoring of the bis-adduct formation upon addition of 8 equiv. bromomalonate (two sequential additions of 2.0 equiv.) in 8h. c) Preparative TLC (SiO_2, Toluene) is used to separate the three regioisomers.

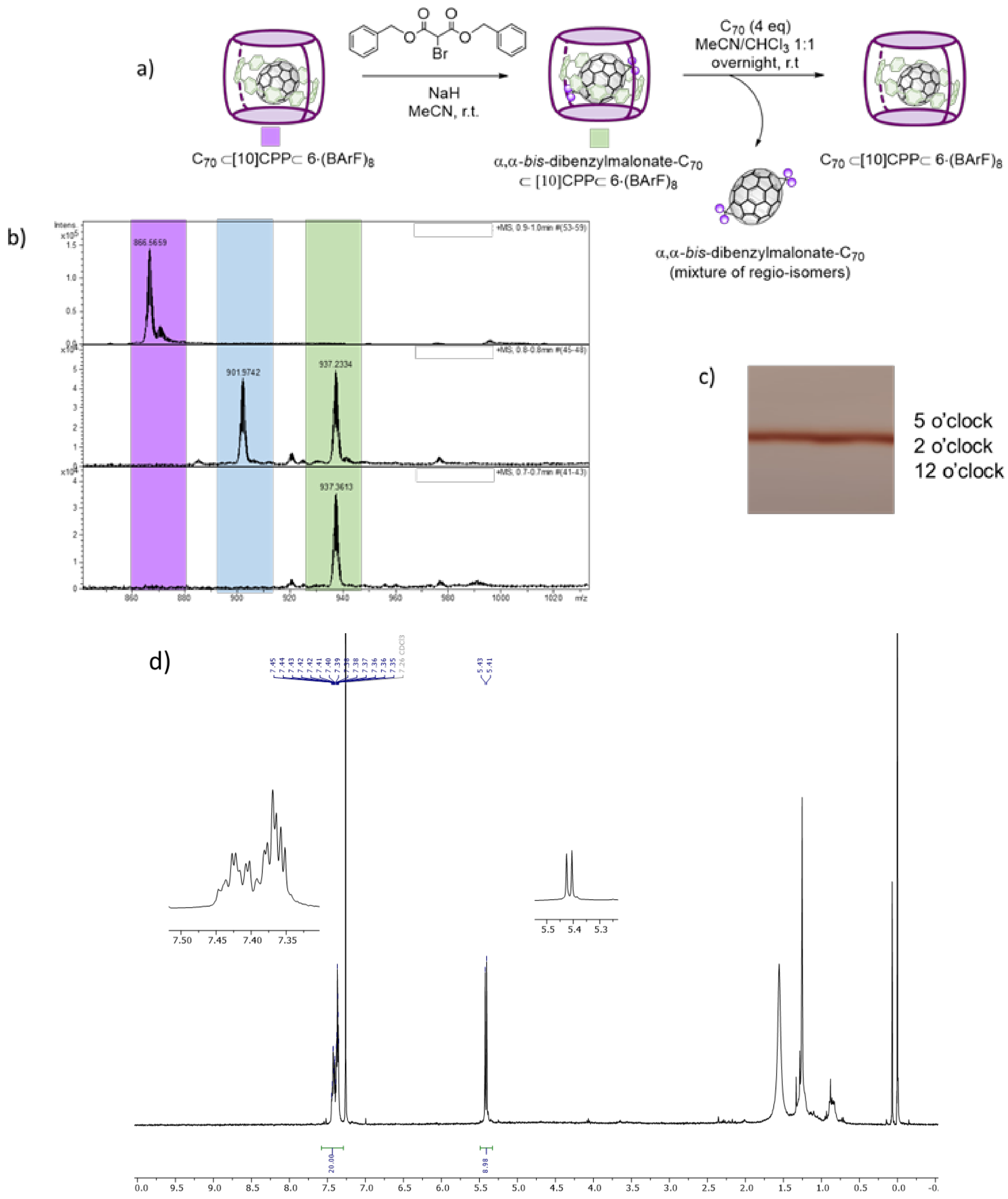

Figure S61. Exposure of_C_{70}⊂[10]CPP⊂**6**·(BArF)$_8$ to Bingel cyclopropanation conditions with dibenzyl bromomalonate leads to the chemo- and regio-selective formation of the bis-adduct 2 o'clock regioisomer; exchange with pristine C_{70} liberates the product. b) HRMS monitoring of the bis-adduct formation upon addition of 10 equiv. bromomalonate (five sequential additions of 2.0 equiv.) in 10h. c) Preparative TLC (SiO_2, Toluene) is used to separate the three regioisomers (just 2 o'clock regioisomer is obtained). d) ^{1}H NMR of the the *bis*-C_{70}-adduct extracted form the Matryoshka-like complex matching with the 2 o'clock regioisomer of α,α-*bis*-dibenzylmalonate-C_{70} (see Figure S45)**.** (400 MHz, $CDCl_3$) δ p.p.m.: δ 7.45 – 7.35 (m, 20H), 5.42 (d, *J* = 8.1 Hz, 8H).

4.5 HRMS Spectra

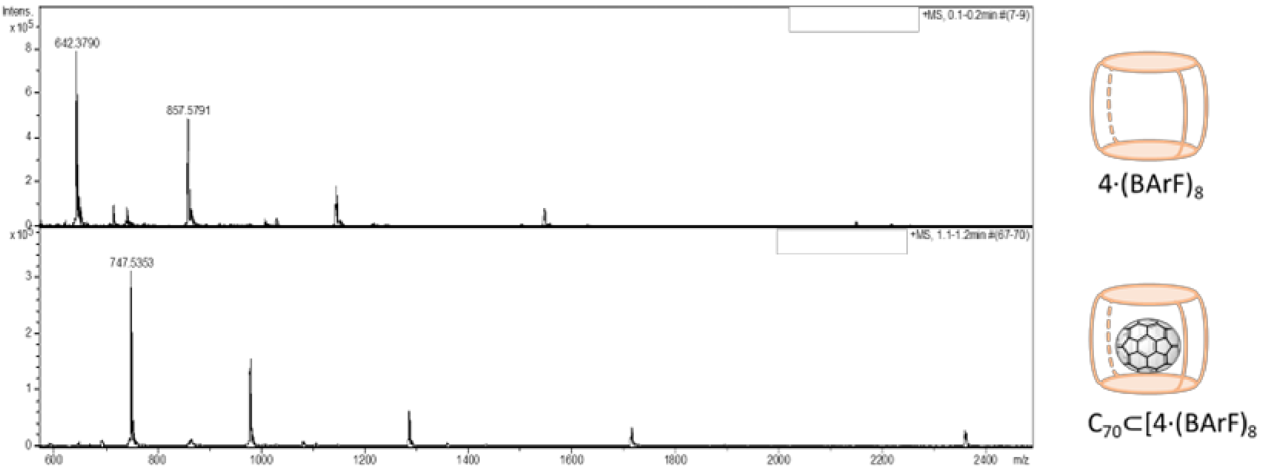

Figure S62. HRMS spectrum of C_{70}⊂**4**·$(BArF)_8$ complex. Sample dissolved in acetonitrile and registered with a Bruker MicroTOF-Q-II mass spectrometer.

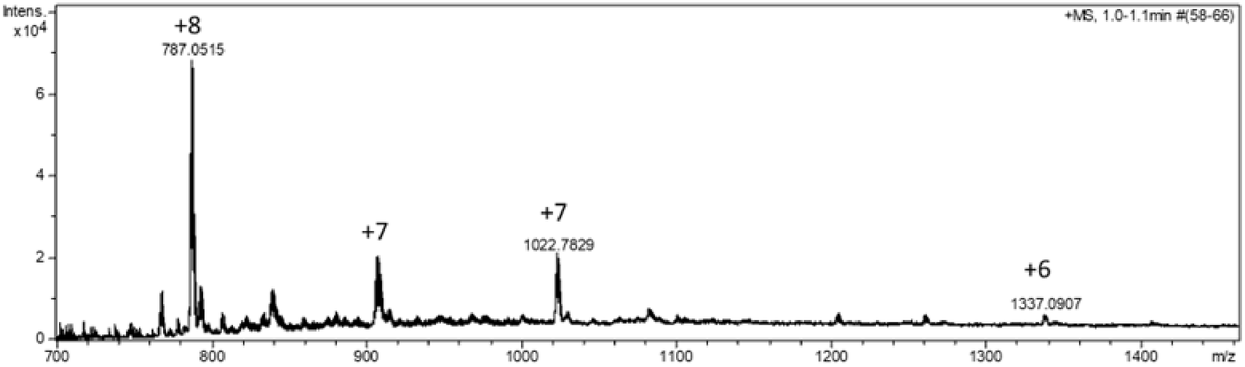

Figure S63. HRMS spectrum of α,α-*bis*-diethylmalonate-C_{70}⊂**4**·$(BArF)_8$. Sample solved in acetonitrile and registered with a Bruker MicroTOF-Q-II mass.

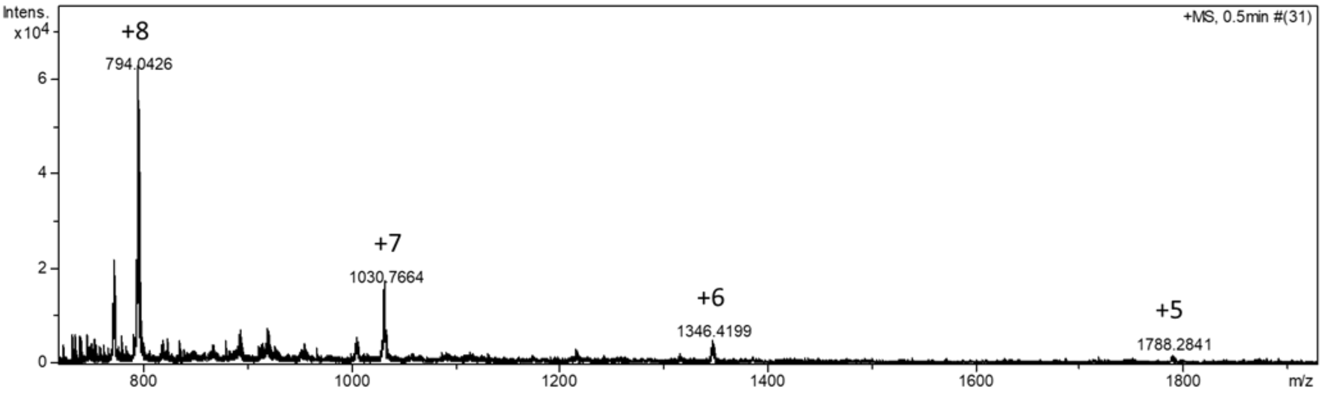

Figure S64. HRMS spectrum of α,α-*bis*-diisopropylmalonate-C_{70}⊂**4**·$(BArF)_8$. Sample solved in acetonitrile and registered with a Bruker MicroTOF-Q-II mass

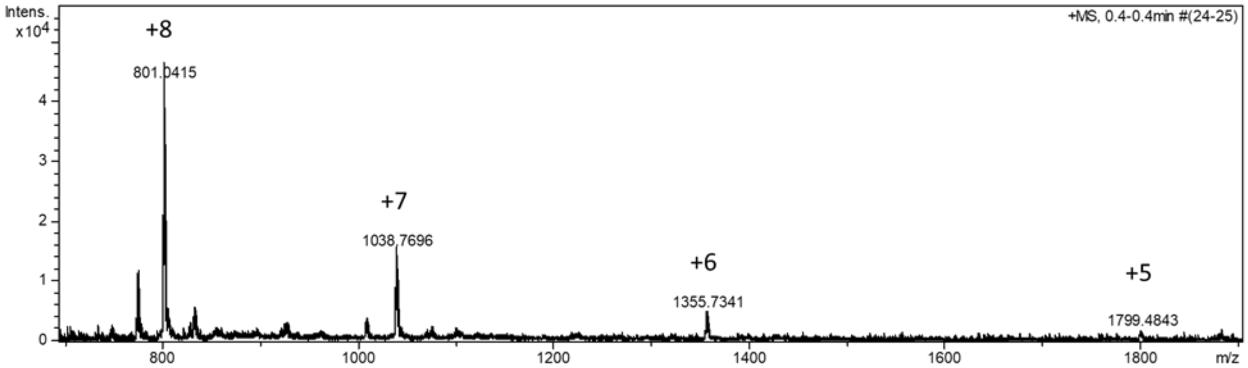

Figure S65. HRMS spectrum of α,α-*bis*-ditertbutylmalonate-C_{70}⊂**4**·$(BArF)_8$. Sample solved in acetonitrile and registered with a Bruker MicroTOF-Q-II mass.

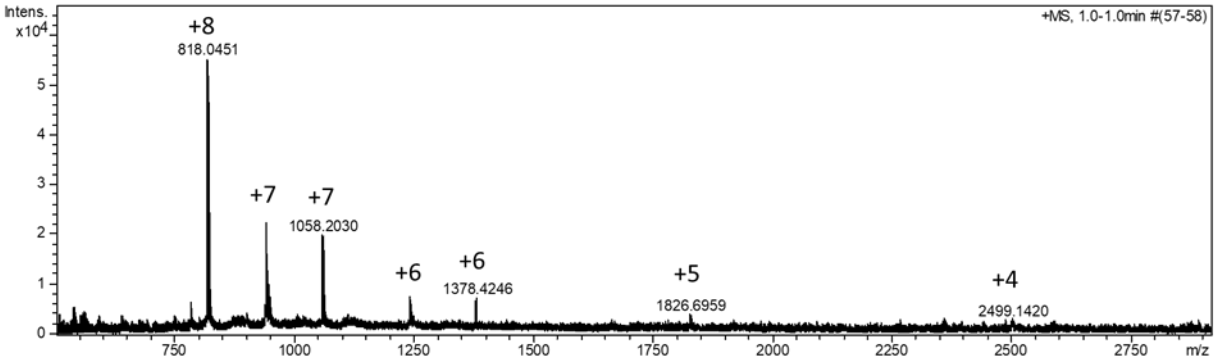

Figure S66. HRMS spectrum of α,α-*bis*-dibenzylmalonate-C_{70}⊂**4**·$(BArF)_8$**.** Sample solved in acetonitrile and registered with a Bruker MicroTOF-Q-II mass spectrometer.

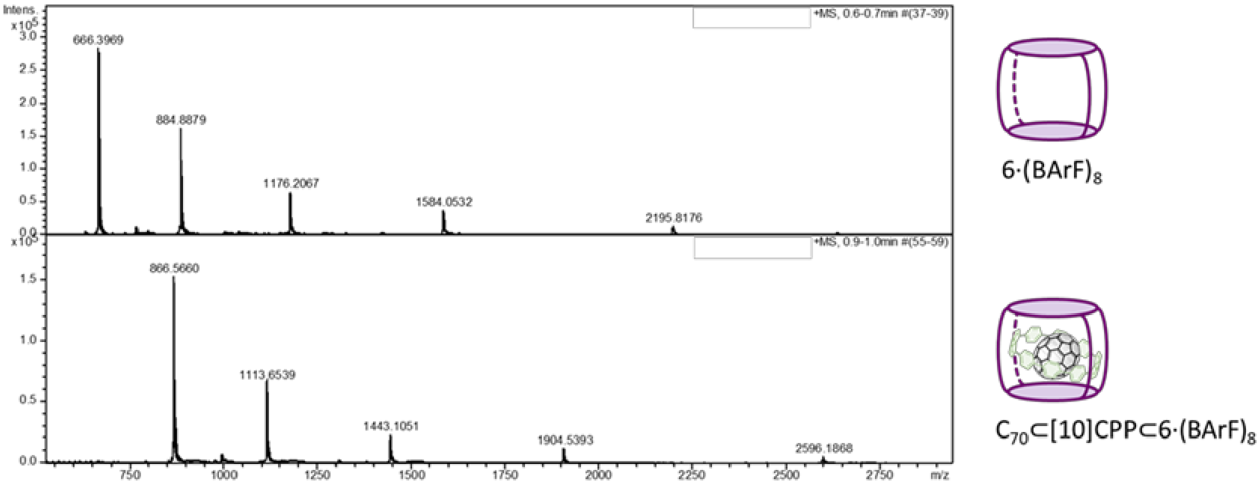

Figure S67. HRMS spectrum of C_{70}⊂[10]CPP⊂**6**·$(BArF)_8$ complex. Sample solved in acetonitrile and registered with a Bruker MicroTOF-Q-II mass spectrometer.

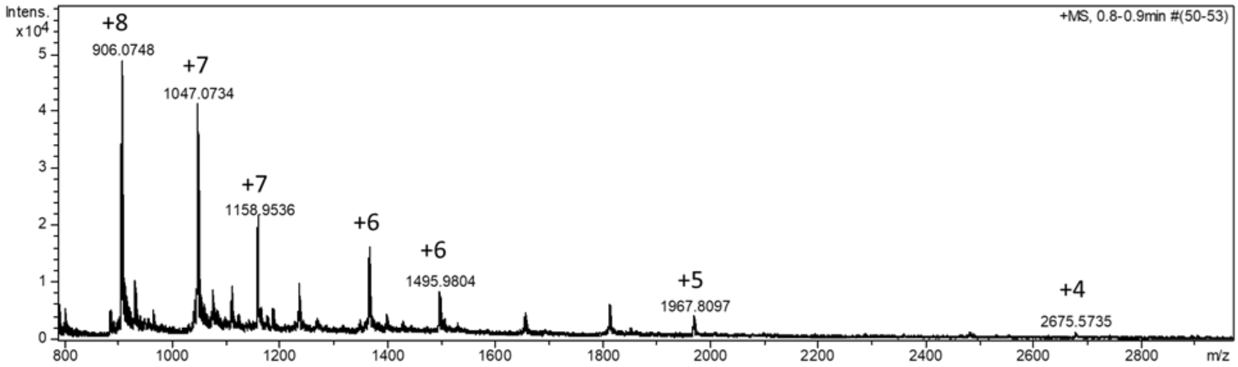

Figure S68. HRMS spectrum of α,α-*bis*-diethylmalonate-C_{70}⊂[10]CPP⊂**6**·$(BArF)_8$. Sample solved in acetonitrile and registered with a Bruker MicroTOF-Q-II mass spectrometer.

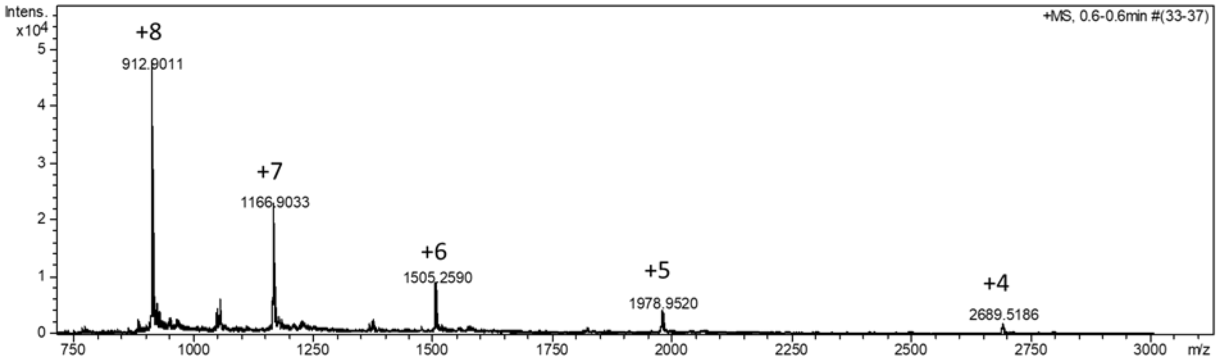

Figure S69. HRMS spectrum of α,α-*bis*-diisopropylmalonate-C_{70}⊂[10]CPP⊂**6**·$(BArF)_8$. Sample solved in acetonitrile and registered with a Bruker MicroTOF-Q-II mass spectrometer.

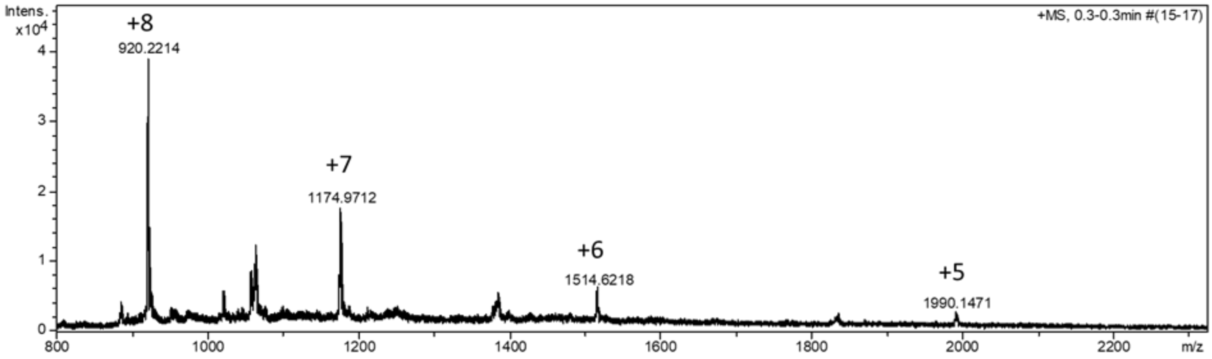

Figure S70. HRMS spectrum of α,α-*bis*-ditertbutylmalonate-C_{70}⊂[10]CPP⊂**6**·$(BArF)_8$. Sample solved in acetonitrile and registered with a Bruker MicroTOF-Q-II mass spectrometer.

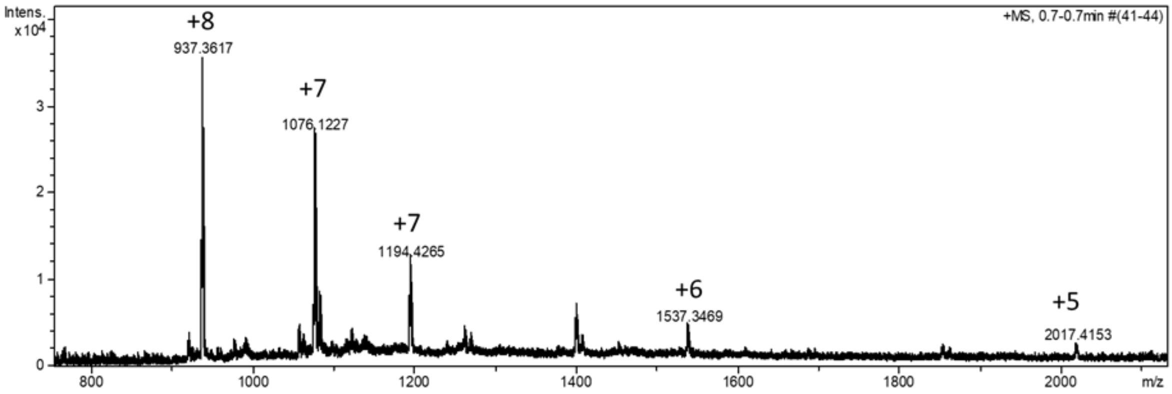

Figure S71. HRMS spectrum of α,α-*bis*-dibenzylmalonate-C_{70} ⊂[10]CPP⊂**7**·(BArF)$_8$ **.** Sample solved in acetonitrile and registered with a Bruker MicroTOF-Q-II mass spectrometer.

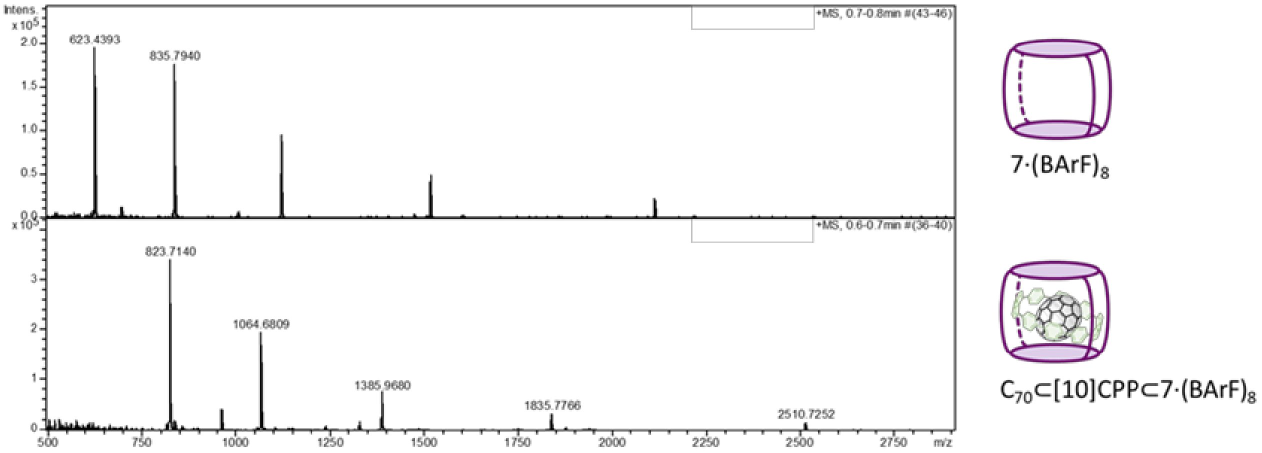

Figure S72. HRMS spectrum of C_{70}⊂[10]CPP⊂**7**·(BArF)$_8$ complex. Sample solved in acetonitrile and registered with a Bruker MicroTOF-Q-II mass spectrometer.

5. X-Ray Diffraction

Crystallographic data for **7·(BArF)$_8$** was collected at 100 K at the XALOC beamline at ALBA synchrotron (λ= 0.82653 Å).[6] Data were indexed, integrated and scaled using the XDS program.[7] Diffraction data was collected up to 1.0 Å resolution, with no reflections observed beyond this value. Increasing the exposure time per frame did not improve the resolution limit. Absorption correction was not applied. The structures were solved by direct methods and subsequently refined by correction of F2 against all reflections, using SHELXT2018 within Olex2 package.[8-9] All non-hydrogen atoms were refined with anisotropic thermal parameters by full-matrix least-squares calculations on F2 using the program SHELXL2018.[3] The hydrogen atoms were calculated in their expected positions with the HFIX instruction of SHELXL2018 and refined as riding atoms with Uiso(H) = 1.5 Ueq(C). A large amount of electron density belonging to solvent molecules was clearly observed, but the exact location of all the atoms could not be refined, and therefore, a solvent mask was applied, accounting for a total of 2731 electrons per unit cell, which is consistent with 68 DMF molecules.[10-11] Due to the low resolution of the crystal data (1.21 Å, with no reflections observed beyond this value) and the high mobility observed in the BArF counteranions, several CF_3 functional group of the BArF were restricted by SADI, DELU, EADP and DFIX.

Table S1. Crystal data and structure refinement for **7**·(BArF)$_8$ (CCDC 2286023).

Identification code	shelx
Empirical formula	$C_{536}H_{370}B_8Cu_8F_{192}N_{32}O_{18}Zn_2$
Formula weight	11920.16
Temperature/K	100
Crystal system	monoclinic
Space group	$P2_1/n$
a/Å	31.3261(7)
b/Å	30.9330(6)
c/Å	37.9458(7)
α/°	90
β/°	98.095(2)
γ/°	90
Volume/Å^3	36403.5(13)
Z	2
ρ_{calc}g/cm^3	1.087
μ/mm^{-1}	0.582
F(000)	12028.0
Crystal size/mm^3	0.06 × 0.06 × 0.04
Radiation	synchrotron (λ = 0.82653)
2Θ range for data collection/°	1.838 to 44.584
Index ranges	-28 ≤ h ≤ 28, 0 ≤ k ≤ 28, 0 ≤ l ≤ 34
Reflections collected	183584
Independent reflections	29387 [R_{int} = 0.1336, R_{sigma} = 0.1301]
Data/restraints/parameters	29387/573/3330
Goodness-of-fit on F^2	1.544
Final R indexes [I>=2σ (I)]	R_1 = 0.1595, wR_2 = 0.4089
Final R indexes [all data]	R_1 = 0.1858, wR_2 = 0.4312
Largest diff. peak/hole / e Å^{-3}	1.43/-0.59

6. MD Simulations

6.1. System preparation and MD simulations protocols

The parametrization of **4**·(BArF)$_8$ and **6**·(BArF)$_8$ and the molecular dynamics (MD) simulations protocols were extracted from our previous work.[12] The force-field parameters of **4**·(BArF)$_8$ and **6**·(BArF)$_8$ were obtained using a protocol that combines the General Amber Force Field (GAFF)[5] and the Metal Center Parameter Builder (MCPB.py)[13] tool using crystallographic X-ray data of the empty nanocapsule as starting structure. Parameters are available in the supplementary information of reference García-Simón et al. *J. Am. Chem. Soc.* 2020, 142, 37, 16051–16063.[12] The parameters for all mono- and bis-C_{70}⊂**4**·(BArF)$_8$ and the C_{70}-Matryoshka complex, i.e. mono- and bis-C_{70}⊂[10]CPP⊂**6**·(BArF), were generated within the antechamber module using the general AMBER force field (GAFF), with partial charges set to fit the electrostatic potential generated at the B3LYP/6-311+G(d) level by the RESP model.[14] The charges were calculated according to the Merz–Singh–Kollman scheme[15] using the Gaussian 16 package.[16]

Molecular Dynamics (MD) simulations protocols and were performed using the GPU code (pmemd)[4] of the AMBER 16 package[17] using acetonitrile (MeCN) as a solvent. The mono- and bis-adducts are manually placed inside the nanocapsule using different orientations as starting point for the MD trajectories. Each complex was immersed in a preequilibrated truncated octahedron box with a 10 Å buffer of MeCN molecules using the leap module, resulting in the addition of around 700 MeCN solvent molecules. The systems were neutralized by addition of eight explicit counter ions (Cl^-). A two-stage geometry optimization approach was performed. The first stage minimizes the positions of solvent molecules and ions imposing positional restraints on the solute by a harmonic potential with a force constant of 500 kcal mol^{-1} $Å^{-2}$, and the second stage is an unrestrained minimization of all the atoms in the simulation cell. The systems are gently heated using six 50 ps steps, incrementing the temperature 50 K each step (0–300 K) under constant volume and periodic boundary conditions. The SHAKE algorithm was employed to constraint the covalent bonds containing hydrogen. Long-range electrostatic effects were modeled using the particle-mesh Ewald method.[18] A 8 Å cutoff was applied to Lennard-Jones and electrostatic interactions. Harmonic restraints of 10 kcal mol^{-1} were applied to the solute, and the Langevin equilibration scheme was used to control and equalize the temperature. The time step was maintained at 2 fs during the heating stages, allowing potential in homogeneities to self-adjust. Each system was then equilibrated without restrains for 2 ns with a 2 fs time step at a constant pressure of 1 atm and a temperature of 300 K. Then, we performed 5 replicates of 0.5 μs for each system gathering a total of 20 μs of simulation time. This data is then clusterized to identify the most relevant orientations of the fullerene adducts with respect to the nanocapsule. Binding affinities (kcal/mol) of bis-adducts were computed using the MM/GBSA method as implemented in AMBER 16.

6.2. MD simulations of mono- and bis-C_{70}⊂4·(BArF)$_8$ with dibenzyl-bromomalonate

The mono-dibenzylmalonate-C_{70} adduct has been simulated inside the nanocapsule **4**·(BArF)$_8$, along with the study of the accessibility of the α bonds. The C_{70} adopts two major orientations, in which the addend is placed at one of the windows or it is stabilized with one phenyl at each side of a clip (Figure S73A). Through the two criteria (A and B), it can be seen that the 5 o'clock position is the most accessible one. This indicates that this position is more at the center of the nanocapsule (criteria A) and closer to one of the windows (criteria B). These results are in agreement with the experimental ones, indicating that the 5 o'clock is the most accessible one so the yield is higher for this bis-adduct.

The final bis-adducts have been simulated also inside the nanocapsule **4**·(BArF)$_8$ (Figure S73B). These results corroborate that the 5 o'clock bis-adduct is the major formed experimentally, since it adopts a stable orientation during all the simulation time. The binding energy calculated for this bis-adduct is -77.51 kcal/mol. For the 2 o'clock a stable conformation is also observed, with a similar binding energy (-76.43 kcal/mol). Finally, for the 12 o'clock, the C_{70} is placed at one of the windows of the nanocapsule in 3 out of 5 replicates. This may indicate that this bis-adduct is not stable inside the cage, preferring to go outside the nanocapsule. This is also corroborated by the binding energy (-72.09 kcal/mol) since it is not so negative in comparison to the other bis-adducts. For this reason, this 12 o'clock bis-adduct is practically not obtained experimentally.

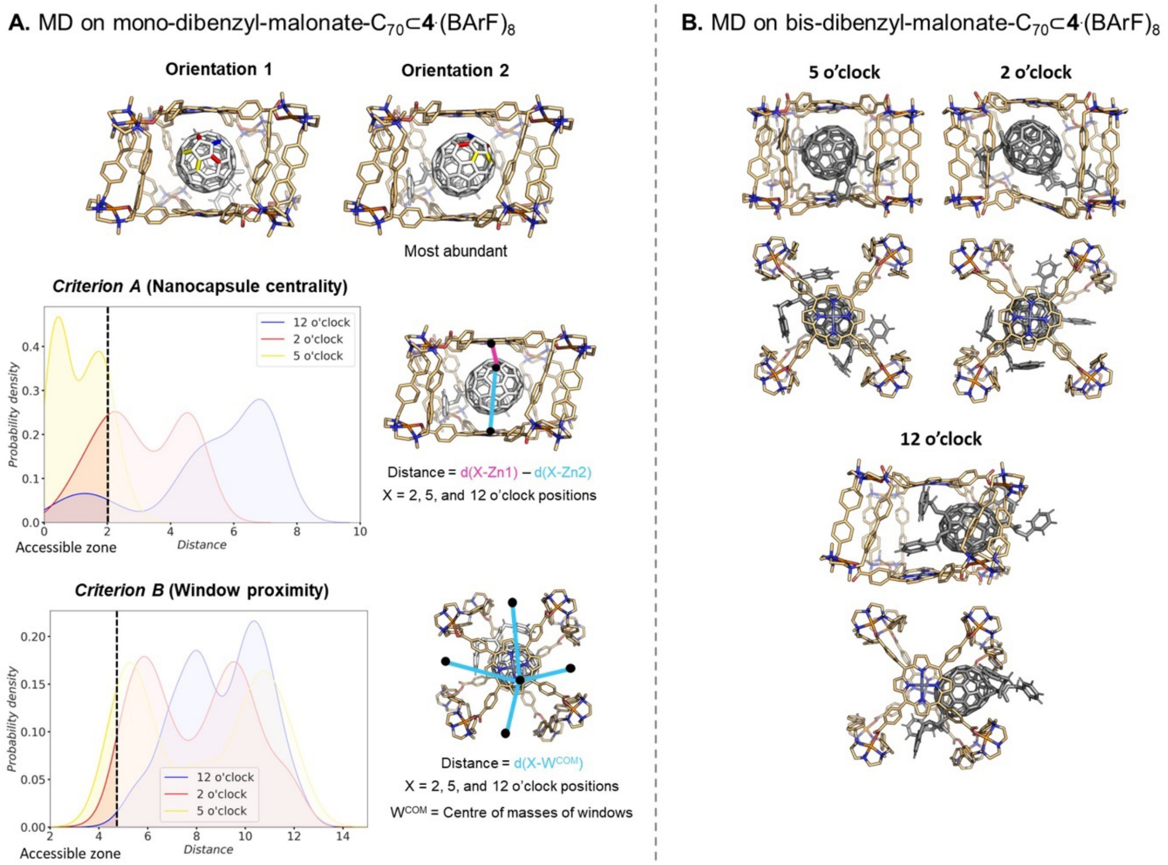

Figure S73. A) MD studies on mono-dibenzyl-malonate-C_{70}⊂**4**·(BArF)$_8$ and accessibility analysis using *Criteria* A and B. B) MD studies on bis-dibenzyl-malonate-C_{70}⊂**4**·(BArF)$_8$.

6.3. MD simulations of mono- and bis-C_{70}⊂**4**·$(BArF)_8$ with diethyl-bromomalonate

The mono-diethylmalonate-C_{70} adduct and the accessibility of the α bonds have been simulated. Since this addend is smaller, the C_{70} can rotate freely inside the cage with the addend placed at the four different windows. For this reason, a variety of different orientations of the mono-adduct can be observed (Figure S74A). With the first criteria A (centrality of the cage), it can be seen clearly that the most accessible α bond is the 5 o'clock, in agreement with the higher yield. The 12 o'clock is the less accessible one, since in some intervals of time during the simulation it is pointing towards one porphyrin. This makes the second addition to this bond practically impossible. With the criteria B it can be seen how the 2 and 5 o'clock positions have very similar distances, which is in agreement with the similar yield obtained experimentally. But if the bands before 4 Å are observed, it can be seen that the 5 o'clock has slightly smaller distances, and maybe for this reason the yield obtained is a little bit higher.

The final bis-adducts have been simulated also inside the nanocapsule **4**·$(BArF)_8$ (Figure S74B). All the bis-adducts adopt a stable orientation with similar binding energy: -64.68 kcal/mol for the 5 o'clock, -67.99 kcal/mol for the 2 o'clock and -65.24 kcal/mol for the 12 o'clock. In the 5 o'clock bis-adduct, the addends are placed at opposite windows, while in the other two bis-adducts (2 and 12 o'clock), one of the addends is interacting with the clip of the nanocapsule.

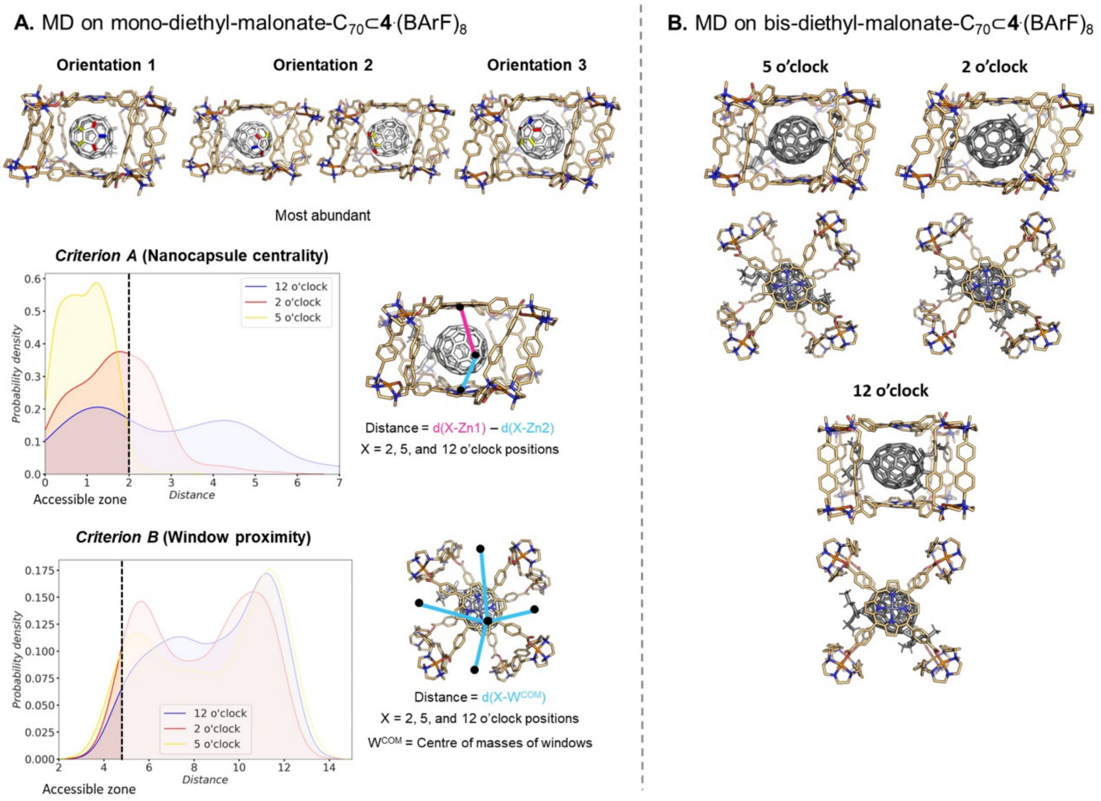

Figure S74. A) MD studies on mono-diethyl-malonate-C_{70}⊂**4**·$(BArF)_8$ and accessibility analysis using *Criteria* A and B. B) MD studies on bis-ethyl-malonate-C_{70}⊂**4**·$(BArF)_8$.

6.4. MD simulations of mono- and bis-C_{70}⊂4·(BArF)$_8$ with diisopropyl-bromomalonate

The mono-diisopropylmalonate-C_{70} adduct inside the nanocapsule **4**·(BArF)$_8$ and the accessibility of the α bonds have been simulated. In this case, the C_{70} moves quite a lot inside the cage, but two principal orientations can be distinguished (Figure S75A). Using the criteria A it can be seen that the 5 o'clock is the most accessible bond, since the difference is closer to 0. But in this case, it is not the bis-adduct obtained in a higher yield experimentally. In the second criteria B, all the bonds present similar distances. This might explain the similar yield obtained experimentally of the 2 and 5 o'clock positions.

The final bis-adducts have also been simulated (Figure S75B). The 5 o'clock bis-adduct adopts a stable conformation inside the cage, with the addends at opposite windows and a binding energy of -70.51 kcal/mol (Figure S75B). In the case of the 2 and 12 o'clock, in 1 out of 5 replicates they are placed at the entry of the cage, while in the other replicates they adopt the same conformation. The binding energy for these two bis-adducts is -66.23 kcal/mol and -68.37 kcal/mol respectively, less negative than the 5 o'clock, probably due to the placement at one of the entrances of the cage.

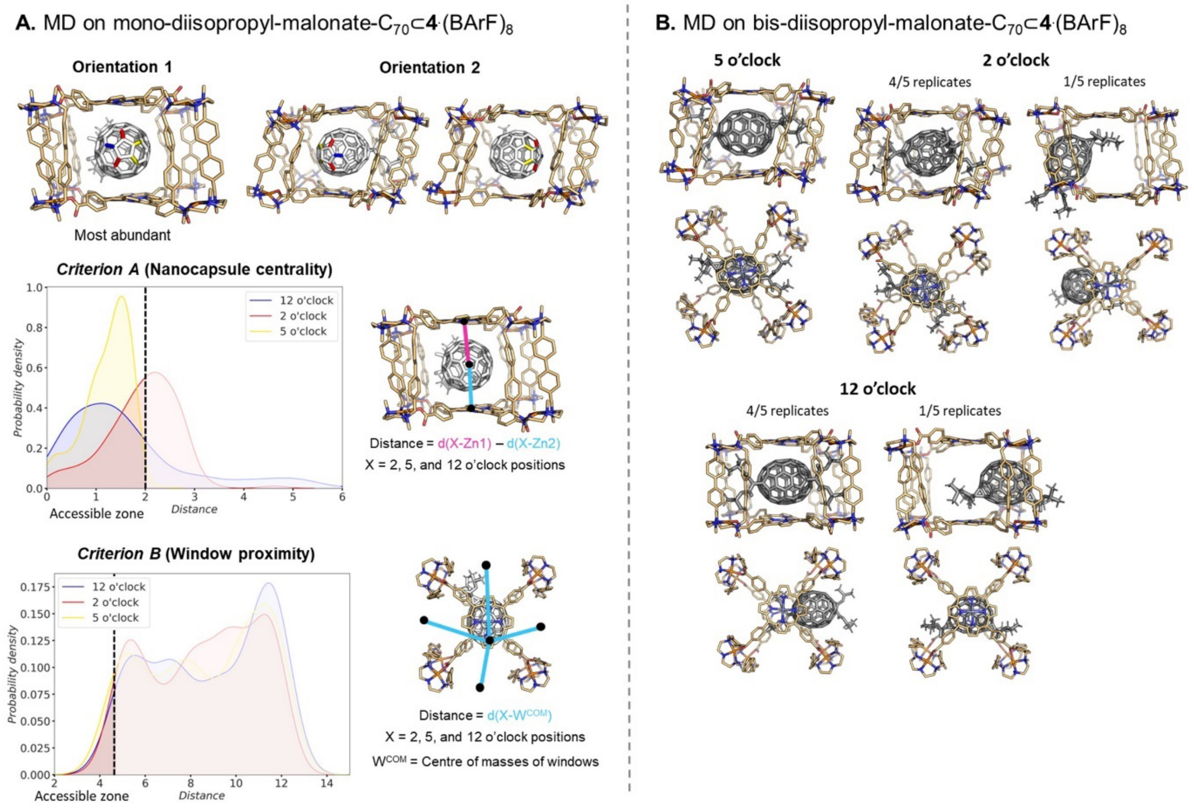

Figure S75. A) MD studies on mono-diisopropyl-malonate-C_{70}⊂**4**·(BArF)$_8$ and accessibility analysis using Criteria A and B. B) MD studies on bis-diisopropyl-malonate-C_{70}⊂**4**·(BArF)$_8$.

6.5. MD simulations of mono- and bis-C_{70}⊂4·(BArF)$_8$ with ditertbutyl-bromomalonate

The mono-ditertbutylmalonate-C_{70} adduct inside the nanocapsule **4**·(BArF) have been simulated. The C_{70} is already placed at one of the windows of the cage in 3 out of 5 replicates (Figure S76A). So, this indicates that the mono-adduct is not stable inside the cage and once it is formed it goes at the entry of the nanocapsule. Then the reaction continues with the C_{70} at one of the windows and for this reason the yields obtained have the same tendency as the bare C_{70}, since the reaction is not controlled by the cage anymore.

The final bis-adducts have also been simulated (Figure S76B). For the 5 o'clock, the C_{70} stays at a stable conformation in 4 of the replicates, but in the other one it is placed at one of the entries of the cage. The binding energy is -66.76 kcal/mol. In the case of the other two bis-adducts (2 and 12 o'clock) the fullerene is placed at one of the windows of the nanocapsule in all the replicates performed. These means that these bis-adducts are not stable inside, which is corroborated with the less negative binding energies of -60.08 kcal/mol for the o'clock -60.19 kcal/mol for the 12 o'clock.

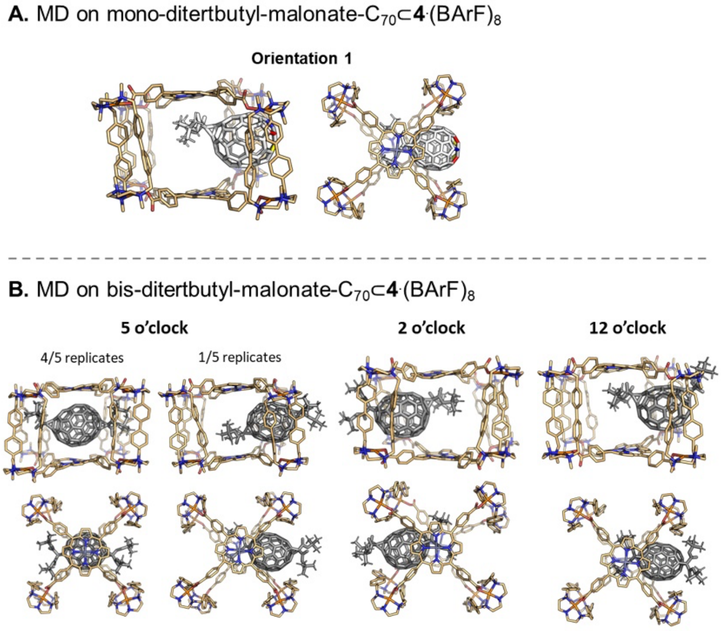

Figure S76. A) MD studies on mono-ditertbutyl-malonate-C_{70}⊂**4**·(BArF)$_8$. B) MD studies on bis-ditertbutyl-malonate-C_{70}⊂**4**·(BArF)$_8$.

6.6. MD simulations of mono- and bis-$C_{70}\subset$[10]CPP$\subset$6·$(BArF)_8$ with dibenzyl-bromomalonate

The mono-dibenzylmalonate-C_{70} adduct inside the matryoshka assembly have been simulated, and the accessibility of the α bonds have been calculated through three criteria. Only two different orientations can be observed in all the replicates performed (Figure S77A). Orientation 1 is the major one and all the positions are quite close and pointing towards one porphyrin of the cage. It is thought that, in this conformation the addition of the second addend does not take place since there is not the required space to react. While in the orientation 2, the C_{70} is more at the middle of the cage, so the positions seem more accessible. Using the criteria A, the position with a difference closer to 0 is the 2 o'clock, which is the only bis-adduct obtained experimentally. With the second criteria B, again the most accessible one is the 2 o'clock, since it is the one that shows smaller distances, closer to one of the windows of the cage. With the last method C, taking into account the [10]CPP, the positions that are at a distance between 7-8 Å are the 12 and one of the 2 o'clock. So, these two are the ones more at the center of the [10]CPP, so they are not blocked by it. But the 2 o'clock is closer to the window, while the 12 is more at the back. So, it would react first and more the 2 o'clock, which is in agreement with the experimental results.

The three final bis-adducts have been also simulated (Figure S77B). In the case of the 5 o'clock, the [10]CPP rotates and is placed vertically respect to the porphyrin planes. This state tenses the cage to high Zn-Zn distances (from 17.5 Å to 19.8 Å) so it is though that it is not possible in solution but in the simulations is observed due to the way the cage is constructed. Also, the binding energy (-48.32 kcal/mol) indicates that this 5 o'clock bis-adduct is not so stable inside the cage, and for this reason it is not formed experimentally. For the 2 o'clock a stable orientation is obtained, in which one of the phenyl substituents in the addend established an interaction with the Zn of the porphyrin, making this 2 o'clock bis-adduct stable inside the nanocapsule. This is also corroborated with the more negative value of the binding energy (-54.76 kcal/mol). Finally, the 12 o'clock bis-adduct can also adapt a stable orientation inside the cage, in this case with each phenyl of the addends placed at one side of the clips and a binding energy of -55.46 kcal/mol.

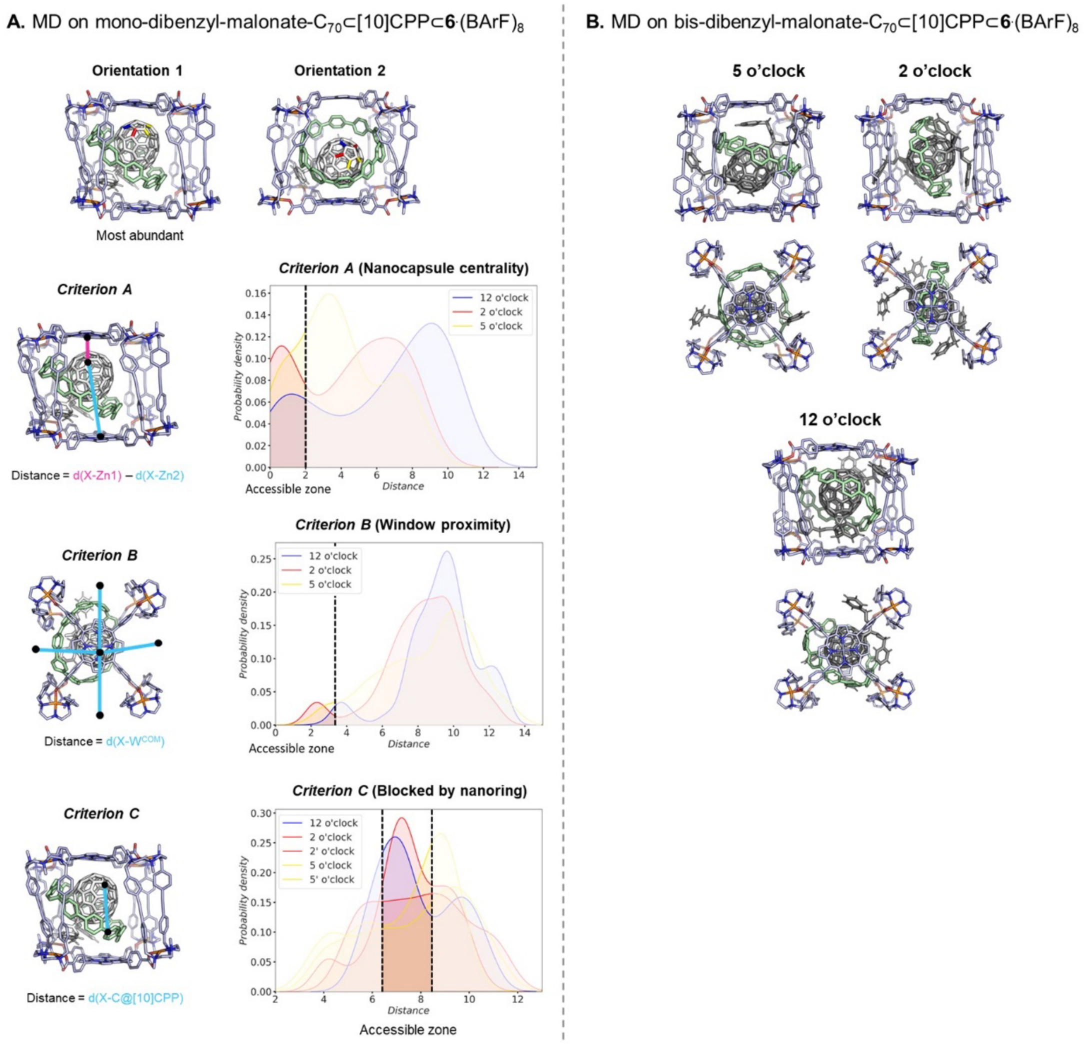

Figure S77. A) MD studies on mono-dibenzyl-malonate-C_{70}⊂[10]CPP⊂**6**·(BArF)$_8$ and accessibility analysis using Criteria A and B. B) MD studies on bis-dibenzyl-malonate-C_{70}⊂[10]CPP⊂**6**·(BArF)$_8$.

6.7. MD simulations of mono- and bis-$C_{70}\subset$[10]CPP$\subset$6·(BArF)$_8$ with diethyl-bromomalonate

The mono-diethylmalonate-C_{70} adduct inside the matryoshka assembly and the accessibility of the α bonds have been simulated. Since the C_{70} can rotate freely inside the matryoshka due to the small size of the addend, different orientations can be distinguished for the mono-addcut (Figure S78A). With the first criteria A, the 2 o'clock is the most accessible bond, which is also the bis-adduct obtained in a higher yield. So, it is the position more at the center of the nanocapsule, not pointing towards any of the porphyrins. In the criteria B, the most accessible positions seem to be the 2 and 5 o'clock. But with the first one, the 5 o'clock is seen to be not so accessible. With the third one (C), where the [10]CPP is taken into account, the 12 o'clock is the one that it is less blocked by the nanoring, but this position is far away from the windows of the cage (criterion B). So, it is expected to obtain a higher yield for the 2 o'clock, as it happens experimentally.

The three final bis-adducts have been also simulated (Figure S78B). For the 5 o'clock and the 2 o'clock, the vertical state, where the [10]CPP is placed vertically respect to the porphyrin planes, is observed in 2 replicates for each bis-adduct. In the other replicates, the C_{70} adopts a stable conformation inside the cage. The binding energy obtained for these two bis-adducts is -46.22 kcal/mol for the 5 o'clock and -49.63 kcal/mol for the 2 o'clock. The 12 o'clock bis-adduct adopts a stable conformation, with a binding energy of -48.45 kcal/mol. So, all the bis-adducts seem to be not so stable inside the cage, since in two of them the vertical state is observed and the binding energy is similar for all of them.

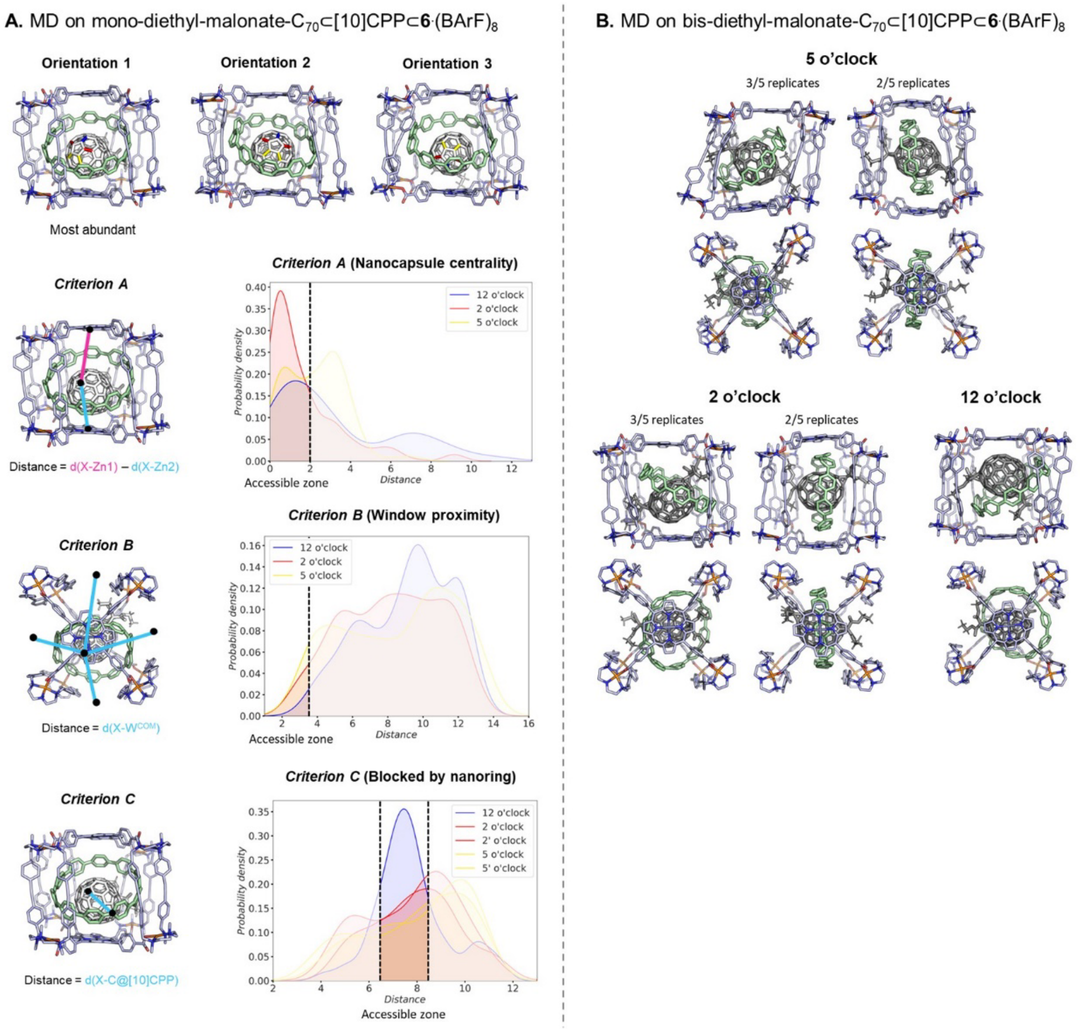

Figure S78. A) MD studies on mono-diethyl-malonate-C_{70}⊂[10]CPP⊂**6**·$(BArF)_8$ and accessibility analysis using Criteria A and B. B) MD studies on bis-diethyl-malonate-C_{70}⊂[10]CPP⊂**6**·$(BArF)_8$.

6.8. MD simulations of mono- and bis-C_{70}⊂[10]CPP⊂6·$(BArF)_8$ with diisopropyl-bromomalonate

The mono-diisopropylmalonate-C_{70} adduct inside the matryoshka assembly have been simulated and also the accessibility of the α bonds have been calculated. Different orientations can be observed (Figure S79A). In two of them (orientation 2 and 3), it seems that all the positions are pointing towards one of the porphyrins, so there is not the required space for the second addend to react. With the first criteria A, the most accessible position is the 12 o'clock, indicating that it is the one more at the center of the cage, not pointing towards any of the porphyrins. This is not in agreement with the experimental results, in which this bis-adduct is obtained in the less yield. In the second criteria B it can be seen that the positions that are more accessible are the 12 and 2 o'clock, they are closer to the window of the cage. But, in comparison to the distances observed in the other malonates with the matryoshka, these ones are larger. In the case of the dibenzyl and diethyl we had distances smaller than 2 Å, but here they are practically above 4 Å. This indicates that the C_{70} is not place at the center of the cage, but it is a little bit out of the cavity through the window where the addend is place. Maybe, it has not explored all the orientations possible for this mono-adduct. Simulating longer times or starting from different initial points may lead to reactive orientations, in which the positions are more accessible. Observing the last criteria C with the [10]CPP, it seems that the position that it is more blocked by the [10]CPP is the 12 o'clock. So, the 12 o'clock would seem the most accessible bond with the criteria A and B, but it is blocked by the [10]CPP, and for this reason it is formed experimentally in less amount.

The final bis-adducts have been also simulated inside the matryoshka assembly (Figure S79B). For the 5 o'clock, the [10]CPP is placed vertical respect to the porphyrin planes in 4 replicates, indicating that this bis-adduct is not so stable inside the cage. The 2 o'clock adopts a stable conformation in all the replicates performed. The 12 o'clock also adopts a stable conformation except in one replicate, in which the [10]CPP adopts the vertical state. With all of this, it seems that the 2 o'clock bis-adduct is the most stable one, corroborating the slightly higher yield.

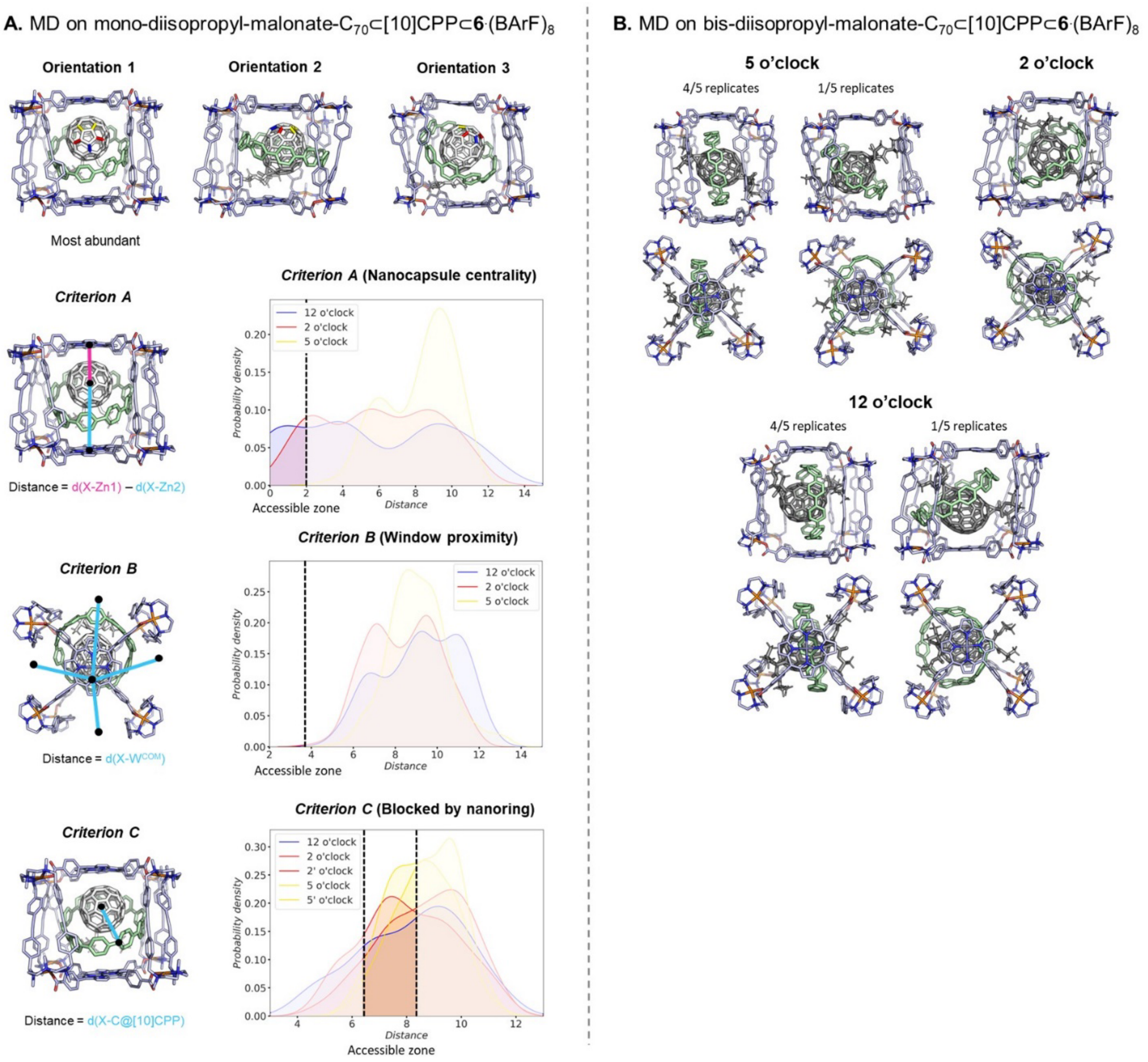

Figure S79. A) MD studies on mono-diisopropyl-malonate-C_{70}⊂[10]CPP⊂**6**·$(BArF)_8$ and accessibility analysis using Criteria A and B. B) MD studies on bis-diisopropyl-malonate-C_{70}⊂[10]CPP⊂**6**·$(BArF)_8$.

6.9. MD simulations of mono- and bis-C_{70}⊂[10]CPP⊂6·(BArF)$_8$ with ditertbutyl-bromomalonate

The mono-ditertbutylmalonate-C_{70} adduct inside the matryoshka assembly have been simulated (Figure S80A). The vertical state is observed in 4 replicates, indicating that the mono-adduct is not so stable inside the cage. In the other replicate it is seen a conformation in which the positions are pointing towards one of the porphyrins. So, this conformation is not a reactive one since there is not the required space for the second addition to take place.

The final bis-adducts have been also simulated (Figure S80B). In the 5 o'clock, the C_{70} adopts a stable orientation in 2 replicates and in the other 3 the [10]CPP is placed vertical. In the 2 o'clock, the vertical state is observed in 4 replicates and also for the 12 o'clock. So, all the bis-adducts with the ditertbutyl bromomalonate are not quite stable inside the cage, due to the bulkiness of the addends.

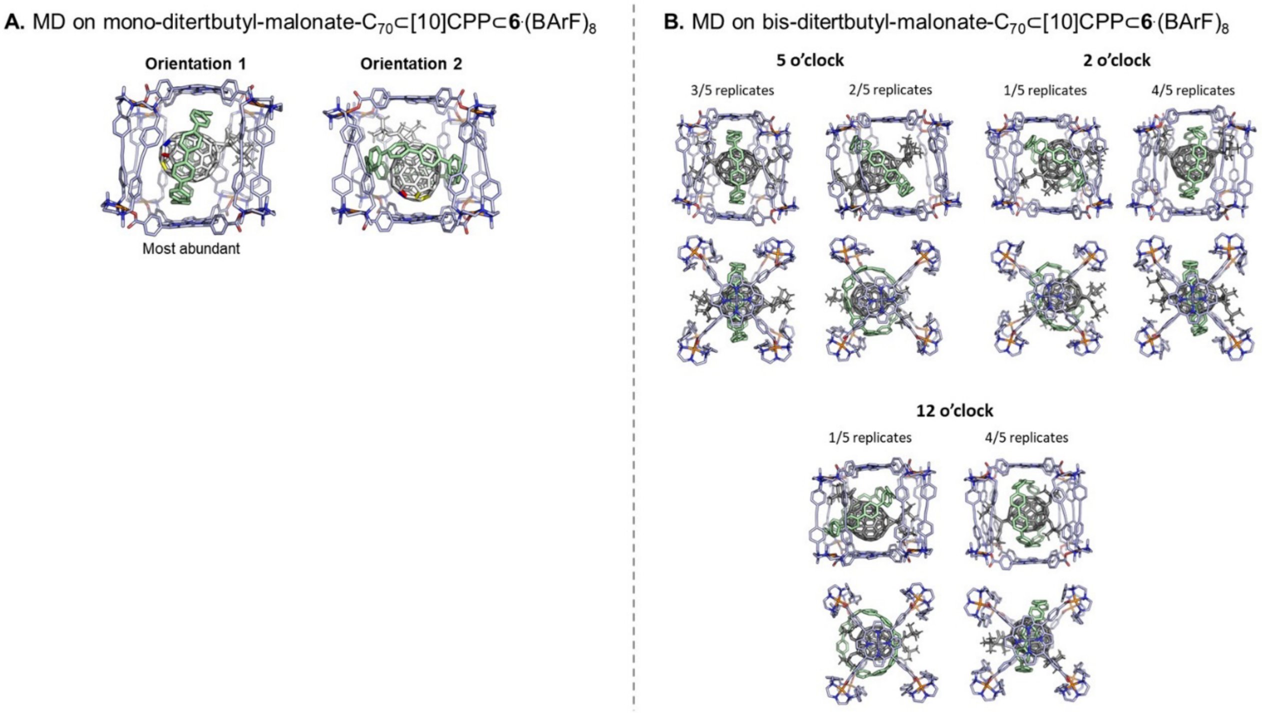

Figure S80. A) MD studies on mono-ditertbutyl-malonate-C_{70}⊂[10]CPP⊂**6**·(BArF)$_8$. B) MD studies on bis-ditertbutyl-malonate-C_{70}⊂[10]CPP ⊂**6**·(BArF)$_8$.

7. Supporting Videos

Video S1. Monodibenzyl-C_{70}⊂**4**·$(BArF)_8$ (Reactive orientation 1)

Video S2. Monodibenzyl-C_{70}⊂**4**·$(BArF)_8$ (Reactive orientation 2 - most abundant)

Video S3. Monodibenzyl-C_{70}⊂[10]CPP⊂**6**·$(BArF)_8$ (Reactive orientation 1 - most abundant)

Video S4. Monodibenzyl-C_{70}⊂[10]CPP⊂**6**·$(BArF)_8$ (Reactive orientation 2)

Video S5. MD simulation of Bis-dibenzyl-C_{70}⊂[10]CPP⊂**6**·$(BArF)_8$ (2 o'clock regio-isomer)

8. References

1. García-Simón, C.; Garcia-Borràs, M.; Gómez, L.; Parella, T.; Osuna, S.; Juanhuix, J.; Imaz, I.; Maspoch, D.; Costas, M.; Ribas, X., Sponge-like molecular cage for purification of fullerenes. *Nat. Commun.* **2014,** *5*, 5557.
2. Ubasart, E.; Borodin, O.; Fuertes-Espinosa, C.; Xu, Y.; García-Simón, C.; Gómez, L.; Juanhuix, J.; Gándara, F.; Imaz, I.; Maspoch, D.; von Delius, M.; Ribas, X., A three-shell supramolecular complex enables the symmetry-mismatched chemo- and regioselective bis-functionalization of C60. *Nat. Chem.* **2021,** *13*, 420-427.
3. He, D.; Du, X.; Xiao, Z.; Ding, L., Methanofullerenes, C60(CH2)n (n = 1, 2, 3), as Building Blocks for High-Performance Acceptors Used in Organic Solar Cells. *Org. Lett.* **2014,** *16*, 612-615.
4. Salomon-Ferrer, R.; Götz, A. W.; Poole, D.; Le Grand, S.; Walker, R. C., Routine Microsecond Molecular Dynamics Simulations with AMBER on GPUs. 2. Explicit Solvent Particle Mesh Ewald. *J. Chem. Theory Comput.* **2013,** *9*, 3878-3888.
5. Wang, J.; Wolf, R. M.; Caldwell, J. W.; Kollman, P. A.; Case, D. A., Development and testing of a general amber force field. *J. Comput. Chem.* **2004,** *25*, 1157-1174.
6. Juanhuix, J.; Gil-Ortiz, F.; G. Cuní; Colldelram, C.; Nicolás, J.; Lidón, J.; Boter, E.; Ruget, C.; Ferrer, S.; Benach, J., Developments in optics and performance at BL13-XALOC, the macromolecular crystallography beamline at the Alba Synchrotron. *J. Synchrotron Rad.* **2014,** *21*, 679-689.
7. Kabsch, W., XDS. *Acta Crystal. D* **2010,** *66*, 125-132.
8. Sheldrick, G., Crystal structure refinement with SHELXL. *Acta Cryst. C* **2015,** *71*, 3-8.
9. Farrugia, L. J., WinGX and ORTEP for Windows: an update. *J. Appl. Cryst.* **2012,** *45*, 849-854.
10. Dolomanov, O. V.; Bourhis, L. J.; Gildea, R. J.; Howard, J. A. K.; Puschmann, H., OLEX2: a complete structure solution, refinement and analysis program. *J. Appl. Cryst.* **2009,** *42*, 339-341.
11. Spek, A., Single-crystal structure validation with the program PLATON. *J. Appl. Cryst.* **2003,** *36*, 7-13.
12. García-Simón, C.; Colomban, C.; Çetin, Y. A.; Gimeno, A.; Pujals, M.; Ubasart, E.; Fuertes-Espinosa, C.; Asad, K.; Chronakis, N.; Costas, M.; Jiménez-Barbero, J.; Feixas, F.; Ribas, X., Complete Dynamic Reconstruction of C60, C70, and (C59N)2 Encapsulation into an Adaptable Supramolecular Nanocapsule. *J. Am. Chem. Soc.* **2020,** *142*, 16051-16063.
13. Li, P.; Merz, K. M., Jr., MCPB.py: A Python Based Metal Center Parameter Builder. *J. Chem. Inf. Model.* **2016,** *56*, 599-604.
14. Bayly, C. I.; Cieplak, P.; Cornell, W.; Kollman, P. A., A well-behaved electrostatic potential based method using charge restraints for deriving atomic charges: the RESP model. *J. Chem. Phys.* **1993,** *97*, 10269-10280.
15. Besler, B. H.; Merz Jr, K. M.; Kollman, P. A., Atomic charges derived from semiempirical methods. *J. Comput. Chem.* **1990,** *11*, 431-439.
16. Frisch, M. J. T., G. W.; Schlegel, H. B.; Scuseria, G. E.; Robb, M. A.; Cheeseman, J. R.; Scalmani, G.; Barone, V.; Petersson, G. A.; Nakatsuji, H.; Li, X.; Caricato, M.; Marenich, A. V.; Bloino, J.; Janesko, B. G.; Gomperts, R.; Mennucci, B.; Hratchian, H. P.; Ortiz, J. V.; Izmaylov, A. F.; Sonnenberg, J. L.; Williams-Young, D.; Ding, F.; Lipparini, F.; Egidi, F.; Goings, J.; Peng, B.; Petrone, A.; Henderson, T.; Ranasinghe, D.; Zakrzewski, V. G.; Gao, J.; Rega, N.; Zheng, G.; Liang, W.; Hada, M.; Ehara, M.; Toyota, K.; Fukuda, R.; Hasegawa, J.; Ishida, M.; Nakajima, T.; Honda, Y.; Kitao, O.; Nakai, H.; Vreven, T.; Throssell, K.; Montgomery, J. A., Jr.; Peralta, J. E.; Ogliaro, F.; Bearpark, M. J.; Heyd, J. J.; Brothers, E. N.; Kudin, K. N.; Staroverov, V. N.; Keith, T. A.; Kobayashi, R.; Normand, J.; Raghavachari, K.; Rendell, A. P.; Burant, J. C.; Iyengar, S. S.; Tomasi, J.; Cossi, M.; Millam, J. M.; Klene, M.; Adamo, C.; Cammi, R.; Ochterski, J. W.; Martin, R. L.; Morokuma, K.; Farkas, O.; Foresman, J. B.; Fox, D. J. *Gaussian 16, Revision A.03*, Gaussian Inc.: Wallingford CT, 2016.
17. D.A. Case; T.E. Cheatham, I.; T.A. Darden; R.E. Duke; T.J. Giese; H. Gohlke; A.W. Goetz; D. Greene; N. Homeyer; S. Izadi; A. Kovalenko; T.S. Lee; S. LeGrand; P. Li; C. Lin; J. Liu; T. Luchko; R. Luo; D. Mermelstein; K.M. Merz; G. Monard; H. Nguyen; I. Omelyan; A. Onufriev; F. Pan; R. Qi; D.R. Roe; A. Roitberg; C. Sagui; C.L. Simmerling; W.M. Botello-Smith; J. Swails; R.C. Walker; J. Wang; R.M. Wolf; X. Wu; L. Xiao; D.M. York; Kollman, P. A., AMBER 2017. *University of California, San Francisco* **2017**.
18. Darden, T.; York, D.; Pedersen, L., Particle mesh Ewald: An N·log(N) method for Ewald sums in large systems. *J. Chem. Phys.* **1993,** *98*, 10089-10092.
